# Plant Secondary Metabolites for the Control of Plant Diseases: Antiviral, Antifungal, and Antibacterial Activities and Their Biotechnological Applications

**DOI:** 10.3390/life16101646

**Published:** 2026-09-30

**Authors:** Ece Basak, Bikem Ulusal, Ayse Betul Bingol, Musa Turker, Cem Bulent Ustundag, Ilknur Yilmaz

**Affiliations:** 1 Department of Bioengineering, Faculty of Chemistry and Metallurgy, Yildiz Technical University, Istanbul 34220, Turkey; ecebasakbio@gmail.com (E.B.); bikem.ulusal@std.yildiz.edu.tr (B.U.); betul.bingol@std.yildiz.edu.tr (A.B.B.); mturker@yildiz.edu.tr (M.T.); cbustun@yildiz.edu.tr (C.B.U.); 2 Health Biotechnology Joint Research and Application Center of Excellence, Yildiz Technical University, Istanbul 34220, Turkey; 3 Department of Molecular Biology and Genetics, Faculty of Engineering and Natural Sciences, İstanbul Atlas University, Istanbul 34408, Turkey

**Keywords:** plant secondary metabolites, phytopathogens, botanical biopesticides, antimicrobial activity, nanoformulations, sustainable plant disease management

## Abstract

Viral, fungal, and bacterial pathogens substantially reduce crop productivity, postharvest quality, and agricultural sustainability. Although synthetic pesticides remain widely used, concerns over environmental persistence, residues, and pathogen resistance have increased interest in safer alternatives. Plant secondary metabolites (PSMs) are promising candidates, but their application is often limited by poor water solubility, volatility, rapid degradation, and inconsistent field performance. This review evaluates the antiviral, antifungal, and antibacterial activities of major PSM classes, including phenolics, terpenoids, nitrogen-containing compounds, and sulfur-containing metabolites, and discusses their biotechnological applications in plant disease management. Recent studies were assessed with emphasis on metabolite sources, target pathogens, mode of action, formulation strategy, and biological efficacy. Evidence indicates that PSMs act through multiple mechanisms, including membrane disruption, inhibition of viral replication, suppression of fungal growth, interference with bacterial metabolism, and activation of plant defense responses. Nanoformulations, encapsulation systems, controlled-release carriers, and green-synthesized nanoparticles can improve stability, delivery, and bioefficacy. Despite this potential, broader agricultural implementation requires further field validation, formulation standardization, long-term stability assessment, and regulatory harmonization.

## 1. Introduction

Plant diseases continue to be a major limitation to global crop production, particularly under intensive cultivation practices and the increasing movement of plant materials between regions. Viral, fungal, and bacterial pathogens can reduce yield, deteriorate postharvest quality, and force growers to rely heavily on synthetic pesticides [1]. Though these chemicals have been the backbone of modern agriculture for decades, their constant and often excessive use has sparked serious concerns regarding environmental persistence, toxicity to non-target organisms, residue accumulation and development of resistance in pathogen populations. Therefore, the plant-derived bioactive compounds are not merely seen as natural defense molecules but are increasingly perceived as practical candidates for sustainable disease management [2].

PSMs are a chemically diverse group of compounds, produced by specialized metabolic pathways. The phenolics, terpenoids, nitrogen-containing compounds and sulfur-containing metabolites are the major classes of compounds involved in plant defense. They do not have a unique mode of antimicrobial activity. Some of the compounds destroy the membranes of pathogens directly, inhibit viral replication, damage fungal hyphae or interfere with bacterial metabolism. Others act more indirectly by inducing plant defense responses, including systemic acquired resistance, antioxidant enzyme activity and pathogenesis-related gene expression. This multi-target nature is especially important because it can reduce the chances of resistance developing compared to synthetic pesticides that target only one site [3,4]. But the road from lab potency to field performance is not easy. Many PSMs are volatile, poorly soluble in water, chemically unstable or rapidly degraded by UV light, fluctuating temperatures and variable soil or leaf surface conditions. Such limitations reduce their persistence and practical reliability [5]. Thus, recent biotechnological approaches such as nanoformulations, encapsulation systems, controlled-release carriers and plant-mediated green synthesis of nanoparticles have received considerable attention. Strategies can improve solubility, protect active compounds, prolong release, and promote interactions on the plant or pathogen surface [6,7].

This review focuses on the antiviral, antifungal and antibacterial activities of PSMs and evaluates their emerging biotechnological applications for plant disease control. Special emphasis is placed on mechanisms of action, formulation-based improvements, current limitations and the remaining gap between successful controlled experiments and real agricultural implementation.

Previous reviews have usually considered plant secondary metabolites either from a phytochemical point of view or in relation to particular pathogen groups. However, there are fewer studies that have combined evidence of antiviral, antifungal and antibacterial activity with formulation-based strategies to see if these compounds can be translated into practical tools for plant protection. This review fills this gap by critically reviewing the antimicrobial properties of PSMs, nanoformulation, encapsulation, controlled release and green nanoparticle-based ways that may increase their stability, delivery and field relevance.

## 2. Plant Secondary Metabolites: Chemical Classes and Mechanistic Relevance

PSMs are products of specialized metabolism and often share metabolic precursors and regulatory networks with primary metabolites or signaling molecules [8,9]. However, for the purpose of plant disease management, they can be classified into four general chemical classes: phenolics, terpenoids, nitrogen-containing metabolites and sulfur-containing metabolites. These classes differ in chemical reactivity and thus in the mechanism by which individual compounds can affect phytopathogens or host defense responses. Lipophilic terpenoids frequently interact with membrane organization and cellular homeostasis, while phenolics often engage in redox regulation and protein or membrane interactions. Nitrogen-containing metabolites are chemically diverse alkaloids, amines, exhibiting distinct, target-specific inhibitory effects on enzymatic machinery, membranes, and nucleic acid-associated processes. Sulfur-containing metabolites including organosulfur compounds and derivatives of glucosinolates can produce reactive species that modify proteins, enzymes, and microbial membranes [4,10,11,12].

**Figure 1:**
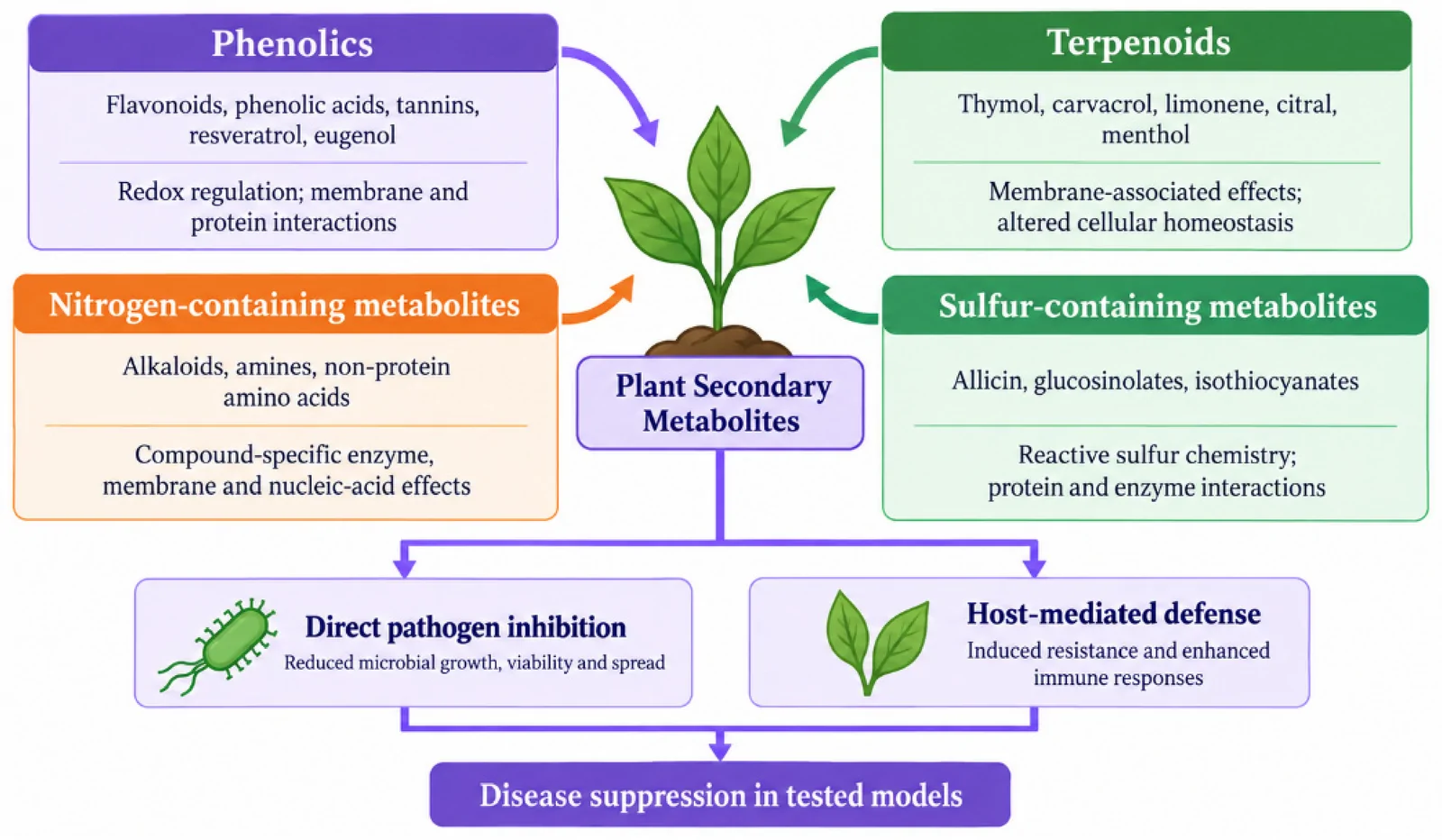
Principal chemical classes of plant secondary metabolites and their dual-mode phytoprotective mechanisms. Phenolics, terpenoids, nitrogen-containing metabolites, and sulfur-containing metabolites differ in chemical properties and biological targets. Depending on the compound and experimental system, their effects may result from direct activity against pathogens or virions, host-mediated defense responses, or a combination of both. Representative compounds and mechanisms are shown; individual metabolites may display overlapping activities. (Created using generative AI).

The biological activities of these phytochemical classes are inherently dynamic rather than mutually exclusive. According to the compound identity, concentration, pathogen and experimental model, single PSMs may act directly on pathogens or virions, induce defense responses in the host plant or both. This mechanistic framework is summarized in Figure 1.

## 3. Antiviral Effects of Plant Secondary Metabolites

**Figure 2:**
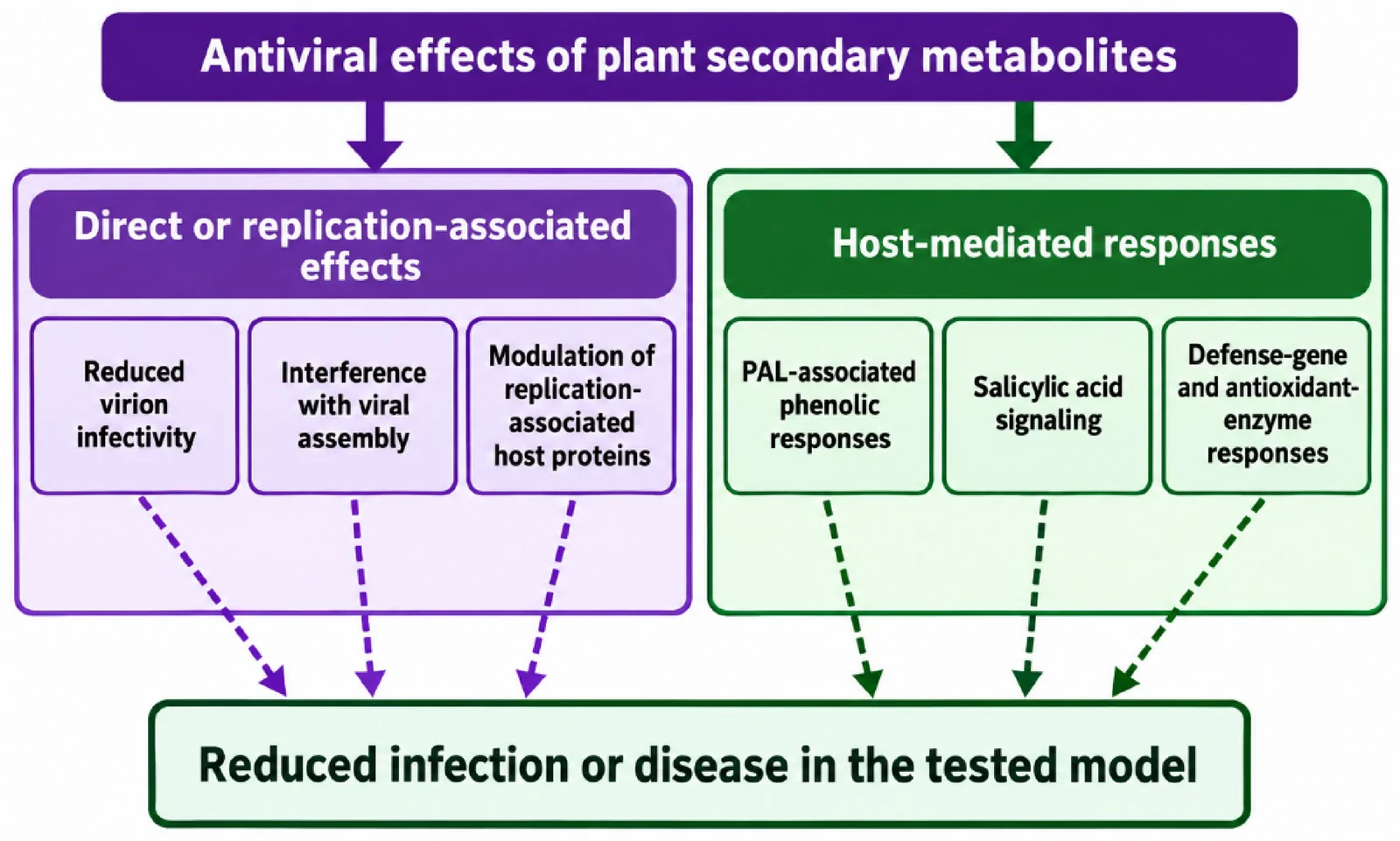
Antiviral effects of plant secondary metabolites through direct, replication-associated, and host-mediated mechanisms. Reported antiviral effects of PSMs include reduced virion infectivity, interference with viral assembly, and modulation of host proteins associated with viral replication. Host-mediated responses may involve PAL-associated phenolic metabolism, salicylic acid signaling, defense-related gene expression, and antioxidant-enzyme responses. These mechanisms can contribute to reduced viral infection or disease severity in experimental models; however, their relative contribution is compound-, host-, virus-, and study-dependent, and changes in defense markers alone should not be interpreted as definitive evidence of systemic acquired resistance. (Created using generative AI).

Plant secondary metabolites can act through several experimentally distinct pathways to exert antiviral activity. Some compounds directly attack viral particles or processes involved in viral replication, while other compounds indirectly decrease infection by activating host defense pathways. Proposed mechanisms include disruption of the virion, interference with coat protein assembly, suppression of host factors required for viral replication, induction of phenylalanine ammonia-lyase (PAL) and salicylic acid (SA)-mediated signaling, as well as the restriction of vector-borne transmission. Local-lesion inhibition, reduced viral accumulation, host-defense activation, and field disease control represent different levels of biological evidence and should not be considered as equivalent outcomes. Figure 2 summarizes the main direct and host-mediated antiviral mechanisms discussed in this section.

### 3.1. Terpenoids

In the control of plant viral diseases, essential oils can be multifaceted natural alternatives. Thanks to their low molecular weight and highly volatile terpenoid compounds, they stand out in this field. In a study conducted by Al-Askar et al. (2025) [13] he antiviral efficacy of the essential oil extracted from rosemary (*Salvia rosmarinus*), against Tobacco mosaic virus (TMV) was investigated using in vivo and in silico methods. GC-MS analysis shows that the chemical backbone was composed of α-terpineol (19.3%), isoborneol (14.1%), and camphor (13.2%). In greenhouse experiments done on *Nicotiana glutinosa* plants, the application of the essential oil at a concentration of 300 µg/mL achieved a high viral inhibition rate of 76.3%, particularly during the therapeutic stage. The protective and direct inactivation effects were determined to be 70.7% and 62.8%, respectively. This high efficacy of the essential oil was linked to the multi-target synergetic effects of its constituents [13].

The potent role of terpenoids in plant viral cycles is not limited to essential oil forms. It is also strongly exhibited in vivo via hydrophilic hydrosols, which are hydrodistillation byproducts of these essential oils [14]. In a study by Taglienti et al. [14], the time-dependent effects of essential oils and hydrosol forms obtained from *Origanum vulgare*, *Thymus vulgaris*, and *Rosmarinus officinalis* were monitored against Zucchini yellow mosaic virus (ZYMV) and Tomato leaf curl New Delhi virus (ToLCNDV) in zucchini plants (*Cucurbita pepo* L.). GC-MS data indicated that *O. vulgare* and *T. vulgaris* extracts contained potent phenolic monoterpenes, specifically carvacrol (88.64–91.59%) and thymol (68.02–57.35%), respectively. Conversely, the *R. officinalis* hydrosol was rich in oxygenated monoterpenes, such as verbenone (33.26%) and camphor (20.92%). While the tested extracts showed no efficacy against ZYMV, they induced a remarkable reduction in viral load against ToLCNDV during both the protective and therapeutic stages. Protective applications conducted simultaneously with inoculation demonstrated that *O. vulgare* essential oil and hydrosol suppressed viral infection. In therapeutic applications targeting post-infection stages, rosemary hydrosol exhibited high success, supporting symptom-free plant development. Investigation into the molecular background of this antiviral success revealed that the therapeutic agents stimulated phenylalanine ammonia-lyase (PAL) gene expression, which regulates PSMs synthesis and biotic stress tolerance, on the 5th day immediately following application. This study demonstrates that terpenoids, particularly hydrosols, can be applied into agricultural protection as potent bio-agents that trigger plant immunity against systemic DNA virus infections when deployed in appropriate formulations [14].

Another virus affecting the *Cucurbitaceae* family is Cucumber mosaic virus (CMV). In a study by Mostafaee et al. [15]., the effects of an essential oil nanoemulsion (EON) from yarrow (*Achillea millefolium*), containing 140.34 mg/g chlorogenic acid and 25.35% 1,8-cineole, were evaluated against CMV. In tests conducted on the host cowpea (*Vigna unguiculata*) plant, a 4% EON formulation applied 2 h post-infection inhibited local lesions by 59.82%. In pumpkin (*Cucurbita pepo*), it reduced CMV accumulation by 63.68%. Furthermore, the nanoemulsion was associated with systemic resistance-related responses, including increased PAL activity, total phenolic content, and antioxidant defense systems such as APX, POD, and CAT [15].

In addition to essential oils and nanoemulsions, limonoids, a triterpenoid subgroup, also provide dual protection against viral infections by triggering plant defense mechanisms, as reported by Yan et al. [16] This study investigated the efficacy of three prieurianin-type limonoids isolated from *Munronia henryi* against TMV. In protective applications, the limonoids tested at a dose of 100 µg/mL exhibited strong inhibition ranging from 70.5% to 81.3%, displaying higher efficacy than the commercial control ningnanmycin (55.6%). Particularly, the compound munronin T increased the salicylic acid content of the plant and strongly stimulated SAR by activating the PAL enzyme. Concurrently, all three compounds downregulated host heat shock protein genes, thereby preventing virus coat protein formation. This study stands out as the first report of an antiviral terpenoid operating simultaneously through two distinct mechanisms: SAR induction and Hsp70 suppression [16].

Thyme, another important essential oil source for developing defense mechanisms and nanotechnological systems, also possesses a terpene-rich molecular profile. In a study conducted by Ekemen and Ulubaş Serçe (2026) [17], thyme essential oil-loaded chitosan-lecithin nanoparticles were synthesized against the Potato virus Y (PVY) pathogen. In aphid transmission assays, this formulation blocked virus transmission to healthy plants by 50.0%, while pure thyme essential oil (TEO) suppressed the virus acquisition capability of aphids by 57.5% [17].

### 3.2. Phenolic Compounds

Natural phytoalexins with phenolic structures, such as resveratrol isolated from *Polygonum cuspidatum* and long used in traditional Chinese medicine, are among the most powerful antiviral agents identified in plants. However, the hydroxyl groups in resveratrol’s phenolic architecture are prone to oxidation, resulting in low chemical stability that limits its practical field application [18]. To overcome this limitation, ester-modified resveratrol derivatives were synthesized and evaluated in vivo against TMV; these derivatives exhibited markedly higher inactivation and protective efficacy than the commercial antiviral ribavirin, representing an important step toward plant-based antiviral solutions [18].

Another way to optimize the antiviral potential of the resveratrol is the integration of sulfonate functional groups into the molecular backbone. In a study conducted by Xia et al. [19]., a series of novel sulfonate derivatives were synthesized to enhance the stability and bioactivity of resveratrol. Among these derivatives tested in vivo for anti-TMV efficacy, compounds I-2, I-5, I-10, II-2, and II-4 outperformed the commercial antiviral ribavirin. Particularly, compound II-4, bearing a cyclopropyl sulfonate structure (59% inactivation, 56% therapeutic, 52% protection), and compound II-2, bearing an ethyl sulfonate structure (55% inactivation, 51% therapeutic, 58% protection), achieved success comparable to ningnanmycin, which was used as the agricultural antiviral comparator in the cited study, and were introduced into the literature as novel antiviral pilot compounds [19].

Another molecule whose field applications are limited due to its natural structure is quercetin, a phenolic flavonol compound found in a wide variety of plants. Although quercetin holds high potential in controlling plant viral diseases, it fails to meet the desired efficiency in applications due to its poor solubility and rapid hydrolysis under natural conditions. To enhance the efficacy and stability of this biofunctional phenolic, Wang et al. developed 117 nm quercetin nanoliposomes (H-TQ-NPs) using biodegradable materials. Mechanism studies determined that quercetin targets host heat shock protein 70 genes (*Nbhsp70er-1* and *Nbhsp70c-A*), which are critical for TMV replication. Compared to free quercetin, the nanoformulation increased the suppression rates of these target genes and related proteins by 33.6% and 42%, respectively. Concurrently, it reduced virus coat protein accumulation by 42.1%. Achieving a protective efficacy of 73.35% under laboratory conditions, the nanoformulation exhibited a 38% higher disease control efficiency than conventional quercetin in field trials, reaching an overall success rate of 68.08% [20].

Although TMV is a widespread pathogen in plants, the antiviral defense mechanisms of phenolic compounds also play a critical role in controlling aggressive, resistance-breaking viruses such as Tomato brown rugose fruit virus (ToBRFV). In a study conducted by Iobbi et al., a methanol extract of *Combretum micranthum* leaves was subjected to high-temperature alkaline autoxidation; catechinic acid derived from the main polyphenols and 4-hydroxybenzoic acid, a thermolytic degradation product, were identified as the primary active components. In in vitro surface contamination and in vivo mechanical inoculation trials, these compounds completely inactivated ToBRFV particles within an exposure time of only 60 s, even at a concentration as low as 32 ppm. No disease symptoms were observed in the treated tomato plants during a 35-day monitoring period. Furthermore, molecular analysis indicated that the viral RNA was completely eliminated. Catechinic acid, in particular, displayed the highest binding energy through its strong interaction network, outperforming the commercial virus inhibitor ribavirin, and stood out as a potential environmental virucide candidate by disrupting the coat protein integrity of the virus [21].

### 3.3. Nitrogen-Containing Compounds

Alkaloids, which are among the most dynamic and chemically diverse members of the plant defense system, stand out as effective bioactive agents in agricultural virology studies by directly disrupting the life cycle of pathogens. *Dendrobium findlayanum*, an important source of alkaloids, possesses a rich molecular profile in addition to its medicinal value. In a study carried out by Song et al., the efficacy of two diterpenoid alkaloids isolated from the plant, findlayine A and dendrofindline B, was evaluated against TMV. In half-leaf assays, dendrofindline B blocked viral infection by 38.6%, exhibiting an efficacy close to that of the commercial control agent ningnanmycin (43.1%) [22].

Greater celandine (*Chelidonium majus*), another important source from the alkaloid class, contains potent isoquinoline alkaloids. In a study carried out by Guo et al., the antiviral effects of chelerythrine, chelidonine, and sanguinarine alkaloids isolated from this plant were applied against TMV. In bioactivity tests, chelerythrine showed virucidal effect at a concentration of 0.5 mg/mL, providing 72.67% inactivation and 77.52% proliferation inhibition. It has structurally fragmented the virus particles. In contrast, although chelidonine did not cause direct destruction of the virus, it provided 64.45% inhibition through an induced-resistance mode of action in tobacco plants at a dose as low as 0.1 mg/mL. This study shows that compounds isolated from the same plant can control TMV, with chelerythrine acting as a direct virus-disrupting agent and chelidonine serving as a potent plant immunity inducer [23].

Within the category of nitrogen-containing compounds, the tobacco plant itself stands out as a rich source of secondary metabolites. In a study conducted by Hu et al., four novel isoindolinone-type alkaloids, designated as nicoisoindole A-D and bearing a rare cyclopenta[f]isoindol-1-one backbone, were isolated from plant stalks. In half-leaf assays, nicoisoindole D exhibited a high antiviral inhibition rate of 67.8% at a concentration of 20 µM. It outperformed the commercial control agent ningnanmycin. In protective assays, nicoisoindole D displayed a protective efficacy of 62.6% on the host plant, demonstrating that tobacco agricultural waste can be utilized in eco-friendly biopesticide designs [24].

These specific alkaloid secondary metabolites not only target the viral coat structure but also establish a broad-spectrum antiviral protective shield by stimulating host anti-stress mechanisms at the genomic level. In a study conducted by Zhao et al., the effects and mechanism of action of berberine molecule, an isoquinoline alkaloid isolated from *Corydalis saxicola* Bunting, were characterized for the first time against Tomato spotted wilt orthotospovirus (TSWV), one of the most destructive pathogens in the plant kingdom. In in vivo trials, the protective (62.00%) and therapeutic (50.00%) efficacy of berberine at a dose of 100 µg/mL surpassed that of the commercial antiviral ningnanmycin, while it blocked viral infection by over 76.00% at a concentration of 200 µg/mL [25].

### 3.4. Sulfur-Containing Compounds

Garlic-derived organosulfur compounds, including alliin, exhibit a broad spectrum of efficacy in plant defense, extending even to viroid-level infections. In a study carried out by Kang and Jeong [26], the inhibitory effects of an extract obtained from garlic (*Allium sativum*) were evaluated under in vitro micropropagation conditions against Hop stunt viroid (HSVd), which causes growth stunting and severe economic losses in grapevines (*Vitis vinifera*). According to dPCR and RT-PCR analyses, the garlic extract applied at a 1000-fold dilution in the tissue culture medium reduced HSVd accumulation by 74.45%, whereas higher concentrations led to contamination and plant damage in the medium [26].

Investigating the field efficacy and defense mechanism of garlic extract, Wang et al. explored the antiviral potential of this natural formulation against a broad spectrum of grapevine viruses in different grapevine cultivars. When applied at a 1:100 dilution under in vitro conditions and tissue cultures, the garlic extract strongly inhibited viral replication, reducing Grapevine leafroll-associated virus 2 and Grapevine fleck virus RNA accumulation to 22% and 20% compared to the controls, respectively. In field trials, this formulation successfully decreased the viral loads of GLRaV-2, GFkV, Grapevine virus A (GVA), Grapevine fanleaf virus (GFLV), and Grapevine rupestris stem pitting-associated virus (GRSPaV) to levels between 15% and 42% relative to the controls [27].

The protective effects of these broad-spectrum organosulfur extracts on viroids and helical RNA viruses are further reinforced by the direct mechanical fragmentation of the coat proteins of resistance-breaking viruses by sulfur-based metabolites. In a study conducted by Iobbi et al., the sulfur profiles of the local Italian garlic ecotype “Aglio di Vessalico” and different species were compared, and their antiviral potentials against ToBRFV were examined. Metabolic analyses confirmed that stable sulfur molecules accumulated at high rates in the extracts depending on the storage process. In the trials, all garlic extracts completely eliminated ToBRFV infectivity even at a concentration as low as 64 ppm, preventing symptom development in the plants [28].

Similar to the mechanical disruptive effect of organosulfur compounds on viral coat structures, sulfur-containing protective allelochemicals of the plant defense system also prevent infection by blocking the self-assembly processes of virus particles. In a study conducted by Liao et al., the sulfur-containing indolium-backbone camalexin phytoalexin, a natural defense metabolite of Brassicaceae family plants such as *Arabidopsis thaliana* and *Camelina sativa*, was selected as a lead compound, and a series of its derivatives were synthesized against TMV. In in vivo antiviral tests, camalexin derivatives achieved a higher inhibition result than the commercial control agent ningnanmycin at a concentration of 500 µg/mL [29]. A mechanistic synthesis of the major PSM classes evaluated against plant viruses is presented in Table 1.

## 4. Antifungal Effects of Plant Secondary Metabolites

PSMs offer a viable alternative to synthetic chemical pesticides in modern crop protection. Synthesized as defense responses to biotic and abiotic stress, these natural compounds show diverse bioactivities, ranging from direct antimicrobial action to triggering systemic resistance pathways within host plants. However, their functional efficacy varies significantly according to applied concentration, target phytopathogen species, and extraction methods, a limitation that highlights the need for standardized, species-specific biopesticide formulations.

### 4.1. Phenolic Compounds

**Figure 3:**
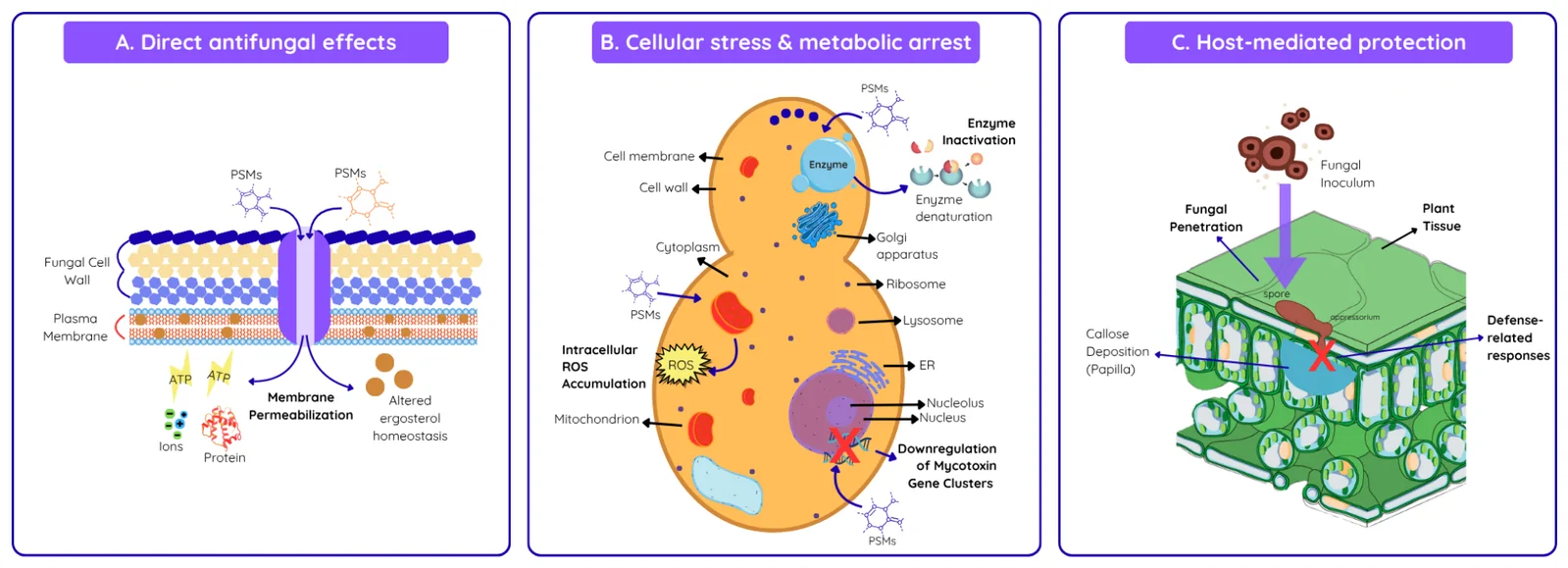
Major antifungal mechanisms associated with plant secondary metabolites across subcellular and tissue scales. (**A**) Direct antifungal effects on fungal cell boundaries, highlighting cell wall disruption, membrane permeabilization, altered ergosterol homeostasis, and unchecked ion/protein leakage culminating in osmotic collapse. (**B**) Intracellular cellular stress and metabolic arrest, depicting mitochondrial dysfunction, intracellular ROS accumulation, enzyme denaturation/inactivation, and the transcriptional downregulation of mycotoxin gene clusters. (**C**) Host-mediated structural protection, illustrating physical barrier reinforcement via callose deposition (papillae formation) at the host–pathogen interface, thereby arresting appressorial penetration and hyphal colonization. Arrows indicate the direction of the depicted mechanistic sequence within each panel. (Created by the authors).

Phenolic compounds represent a major metabolite class in plant defense mechanisms, contributing to both direct pathogen toxicity and structural barrier reinforcement. Extracts of clove (*Syzygium aromaticum*), rich in eugenol (52.88%) and eugenyl acetate (21.95%), achieved complete (100%) growth inhibition against *Colletotrichum acutatum* and *Botrytis cinerea* [30]. Applying clove essential oil at 100 µL/mL fully suppressed brown rot severity on apple fruits (0% disease severity), matching the performance of commercial synthetic fungicides [31]. In marigold cultivation, clove oil completely halted the mycelial growth of both *Fusarium solani* and *Sclerotinia sclerotiorum*; treated plants exhibited significant accumulation of total phenolics and chlorophyll, confirming host defense reinforcement [32]. Although primary bioassays against *C. acutatum* established phenotypic efficacy without profiling specific molecular targets [30], the pronounced lipophilicity of eugenol strongly correlates with membrane destabilization and functional perturbation of lipid metabolism (Figure 3A), reflecting a mechanistic disruption of the fungal envelope rather than an isolated fungistatic arrest [33].

### 4.2. Terpenoids

Terpenoids function as lipophilic agents that penetrate fungal membranes, disrupt lipid organization, and induce intracellular oxidative stress (Figure 3A,B). The isomeric monoterpenes thymol and carvacrol target fungal cell membranes. Carvacrol- and thymol-rich essential oils from Greek oregano (*Origanum heracleoticum*) and common thyme (*Thymus vulgaris*) caused extensive plasmolysis, membrane permeabilization, and nucleic acid leakage (Figure 3A) in *Botrytis cinerea hyphae*. When evaluated in vivo on harvested strawberries, these treatments reduced disease severity by 50% and limited fruit weight loss by up to 40% [34]. Against *Cercospora beticola*, thyme essential oil exhibited low minimum inhibitory concentrations (MIC: 0.313 mL/L), while two-year field evaluations confirmed a marked decrease in leaf spot symptoms relative to untreated controls [35]. Applied as a seed dressing in spinach, thyme oil lowered *Fusarium oxysporum* incidence by 54%, outperforming oregano essential oil [36].

Vapor-phase application of dittany (*Origanum dictamnus*) oil reduced lesion area on peppers by up to 44.7%, demonstrating a residual protective effect after pre-exposure [37]. Similarly, oregano essential oil increased PAL, chitinase (CHI), and beta-1,3-glucanase (GLU) activities in blueberry fruits, with some defense-enzyme activities increasing by up to 3.8-fold [38]. Lemongrass (*Cymbopogon citratus*), through its principal constituent citral, completely arrested the growth of *Botrytis cinerea* and *Alternaria linariae*, outperforming commercial fungicide controls [39,40]. In in vivo bean assays, lemongrass essential oil reduced anthracnose severity by 29% while significantly elevating CHI and PPO activities [41].

The efficacy of thyme and lemongrass essential oils against *Alternaria linariae* has been linked to the reinforcement of structural barriers. It was demonstrated for the first time that applying thyme essential oil induces callose deposition extending from the leaf epidermis to the mesophyll, thereby blocking the penetration of fungal attachment structures (Figure 3C) [40]. Formulating the monoterpenoid geraniol into nanocarriers stabilized malondialdehyde (MDA) levels in cucumber seedlings, priming host tissues against subsequent oxidative stress [42].

The broad-spectrum antifungal capacity of terpenoids is further supported by oregano essential oil, which causes irreversible structural damage to the hyphae of *Rhizoctonia solani* and halts sclerotia formation at 50 µg/mL [43]. Applied to cucumber seedlings, celery seed oil (*Apium graveolens*) rich in D-limonene and beta-selinene stimulated a 71% increase in endogenous phenolic content, reducing disease severity to 41.25% [44]. Volatile constituents such as myrcene and humulene from hop essential oil (*Humulus lupulus*) alter fungal membrane fluidity and disrupt lipid organization by stripping ergosterol molecules via desorption processes (altered ergosterol homeostasis) (Figure 3A) [45].

The biological activity of citrus-derived monoterpenes like limonene varies by target species. Grapefruit oil (*Citrus grandis*) showed limited efficacy against *Cercospora beticola*, whereas vapor-phase treatments of cinnamon bark (*Cinnamomum verum*) and oregano achieved complete (100%) inhibition of *Penicillium digitatum* mycelial growth and sporulation [35,46]. In addition, intra-genus phytochemical variations within *Mentha* dictate antifungal outcomes: peppermint (*Mentha piperita*) essential oil, dominated by germacrene D, exhibits strong activity against *Botrytis cinerea*, whereas wild mint (*Mentha longifolia*) shows minimal efficacy against the same pathogen due to its contrasting chemical profile [47].

### 4.3. Nitrogen-Containing Compounds (Alkaloids)

Nitrogen-containing compounds, particularly alkaloids, can disrupt membrane function and cellular homeostasis, although their mechanisms vary substantially by compound (Figure 3). Leaf extracts of large-leaved lupine (*Lupinus polyphyllus*), rich in quinolizidine alkaloids, suppressed the mycelial growth of *Fusarium oxysporum* by over 95%, matching the performance of commercial fungicides [48]. Similarly, ultrasound-assisted extraction (UAE) fractions from *Lupinus mirabilis* demonstrated high antifungal activity, a response attributed to synergistic interactions between sparteine and lupanine [49].

Piperine from black pepper (*Piper nigrum*) and capsaicin from habanero pepper (*Capsicum chinense*) inhibit *Aspergillus parasiticus* growth and aflatoxin biosynthesis while modulating fungal antioxidant responses. Piperine exposure increased catalase (CAT) activity by 65.5%, and both compounds downregulated genes involved in aflatoxin biosynthesis (Figure 3B) [50].

### 4.4. Sulfur-Containing Compounds

Sulfur-containing metabolites are particularly effective in soil-borne pathogen control due to their volatile “bio-fumigation” potential. Propyl-propane-thiosulfinate (PTS) and propyl-propane-thiosulfonate (PTSO) derived from onion (*Allium cepa*) extracts demonstrated strong vapor-phase fungicidal activity against *Verticillium dahliae* [51]. When applied to soil, these organosulfur derivatives reduced pathogen populations by 99.99%, an efficacy level matching methyl bromide, and lowered in vivo disease severity from 100% to 20% [51].

More generally, dose calibration is also critical across volatile PSM classes, including terpenoid-rich essential oils. When evaluating thyme (*Thymus vulgaris*) and sage (*Salvia officinalis*) essential oils against *Fusarium culmorum* and *Fusarium fujikuroi*, low concentrations provided effective fungal inhibition, whereas elevated doses impaired maize and wheat seed germination [52]. Conversely, higher dosages (10 mL) of coriander (*Coriandrum sativum*) and geranium (*Pelargonium graveolens*) essential oils achieved complete (100%) mycelial inhibition across all tested fungal strains, illustrating the broad-spectrum biopesticidal potential of these botanical extracts when applied within safe physiological limits [52].

In summary, the antifungal action of PSMs extends beyond direct toxicity to include host defense activation (Figure 3C). These compounds consistently stimulate key defense-related enzymes (PAL, POX, PPO, GLU, CHI) and enhance antioxidant system activity (SOD, CAT, APX) in host tissues [32,40,44]. Transitioning away from synthetic chemical pesticides requires a clear understanding of the in vivo stability and multi-target mechanisms of these bioactives. Future research should focus on advanced nano-encapsulation techniques to improve the persistence of volatile constituents and optimize species-specific biopesticide formulations. A mechanistic synthesis of the major PSM classes evaluated against phytopathogenic fungi is presented in Table 2.

## 5. Antibacterial Effects of Plant Secondary Metabolites

PSMs represent sustainable, eco-friendly, and biodegradable alternatives for managing phytopathogenic bacteria that cause substantial economic losses in crop production. The continuous rise in bacterial resistance to synthetic pesticides, coupled with the environmental hazards of chemical residues, has driven agricultural research toward natural, biotechnological solutions. Because many PSMs act through multiple cellular targets, they may reduce the likelihood of single-step resistance compared with compounds acting at a single site; however, resistance evolution has rarely been evaluated directly in phytopathogenic bacteria [3,4]. In some systems, plant extracts can directly suppress pathogen growth while also influencing host defense responses [4].

### 5.1. Phenolic Compounds

**Figure 4:**
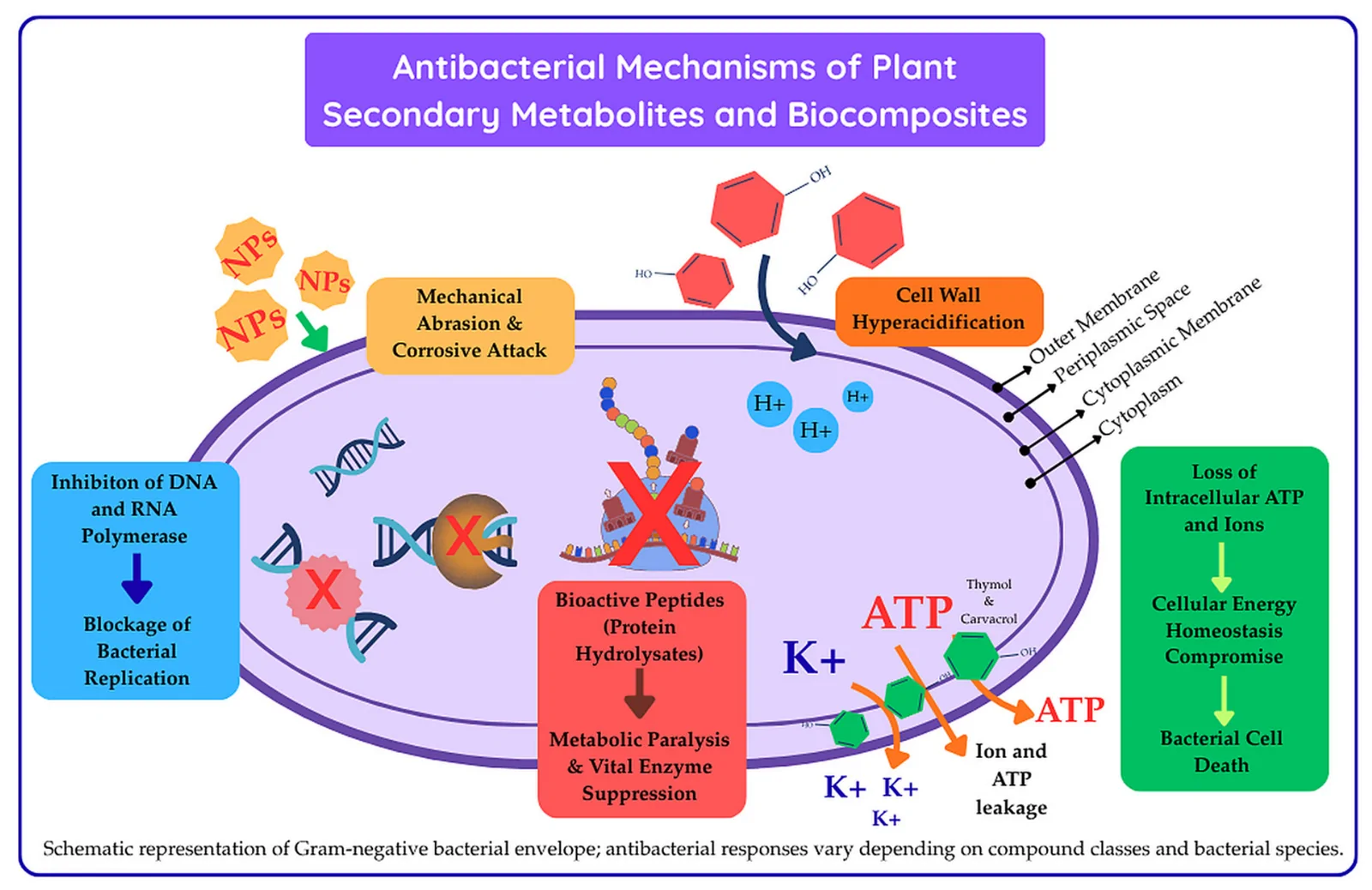
Major antibacterial mechanisms associated with plant secondary metabolites. Reported antibacterial effects include disruption of the bacterial cell envelope, increased membrane permeability and leakage of intracellular components such as ATP and K^+^, interference with respiratory and metabolic processes, inhibition of enzymes and nucleic-acid-associated processes, and suppression of biofilm formation or virulence-related behavior. Some plant-derived formulations may additionally produce carrier- or nanoparticle-dependent effects that should be distinguished from the intrinsic activity of the secondary metabolite itself. The relative contribution of these mechanisms varies according to the compound, bacterial species, formulation, and experimental model. Arrows indicate process direction and ion/ATP leakage, whereas red crosses denote inhibition or blockade; colors group mechanistic categories and do not indicate effect magnitude. (Created by the authors).

Phenolic compounds, including thymol, carvacrol, eugenol, and hydroxytyrosol, represent some of the most effective plant-derived antibacterial agents. Carvacrol and thymol, abundant in thyme (*Thymus* spp.) essential oils, are widely associated with membrane permeabilization and disruption of cellular homeostasis (Figure 4) [54,55,56]. Extracts from creosote bush (*Larrea tridentata*), rich in polyphenolics, have shown strong bactericidal activity against multidrug-resistant phytopathogens, including *Clavibacter michiganensis* and *Xanthomonas campestris* [57]. In in vivo trials, maidenhair tree (*Ginkgo biloba*) leaf extracts-enriched with catechol derivatives-fully protected tomato plants against *C. michiganensis* and pea plants against *Pseudomonas syringae* without inducing phytotoxic symptoms [58]. Similarly, thymol-rich essential oils from ajwain (*Carum copticum*) achieved high bactericidal efficacy against *P. syringae* [59]. Hydroxytyrosol isolated from olive mill wastewater efficiently suppressed Gram-negative pathogens such as *Salmonella enterica* and *Escherichia coli*, with its antibacterial potency significantly enhanced in low-pH environments [60,61]. Additionally, while the antibacterial activity of sage (*Salvia* spp.) extracts correlates with their rosmarinic acid content, their overall performance is largely driven by synergistic interactions within their complex phenolic profiles [62]. Phenolic compounds induce cell wall hyperacidification through proton accumulation in the periplasmic space, which destabilizes envelope integrity and disrupts cellular homeostasis (Figure 4) [63].

### 5.2. Terpenoids

Terpenoids encompass a wide spectrum, including monoterpenes, sesquiterpenes, and diterpenes, and play a critical role in plant defense. Terpinen-4-ol obtained from tea tree (*Melaleuca alternifolia*) oil and 1,8-cineole in eucalyptus (*Eucalyptus obliqua*) oil inhibit cellular respiration by increasing bacterial cytoplasmic membrane permeability, triggering rapid loss of intracellular ATP and K^+^ ions and subsequent collapse of cellular energy homeostasis [64,65]. Lemongrass (*Cymbopogon citratus*) oil completely inhibited *Xanthomonas oryzae* within 75 min, while citral was the most active major component in bactericidal assays [66]. Terpenoid-rich essential oils from species like rockrose (*Cistus ladaniferus*) and apple mint (*Mentha suaveolens*) showed strong suppression against Gram-positive pathogens such as *Clavibacter michiganensis* with very low MIC values [67]. Conversely, nepetalactone within catnip (*Nepeta* spp.) offers a genetically ideal chemotype for biopesticide production [68]. Peppermint (*Mentha piperita*) essential oil completely prevented *Agrobacterium tumefaciens* infections under greenhouse conditions, providing plant protection by suppressing tumor formation [69]. The efficacy of terpenoids is frequently dependent on the synergistic interactions these compounds establish with one another or with other phenolics [64,70].

### 5.3. Nitrogen-Containing Metabolites and Peptide-Based Bioactives

Nitrogen-containing metabolites and peptide-based bioactives can affect intracellular metabolic pathways through distinct mode-of-action profiles. For instance, low-molecular-weight protein hydrolysates (specifically the PQLAVF and MDRFL peptides) isolated from agricultural waste induced metabolic paralysis and vital enzyme suppression in phytopathogens such as *Xanthomonas oryzae* and *Pectobacterium carotovorum* by suppressing vital metabolic processes without causing primary cell membrane lysis (Figure 4) [71]. *A chemically distinct example is pomegranate (Punica granatum) peel extract incorporated into bacterial cellulose, which showed antibacterial activity against Staphylococcus aureus and Escherichia coli, producing 100% and 50% growth reductions, respectively; however, this was a biomedical composite model rather than a phytopathogen system* [72]. Polyphenol-rich plant extracts and specialized nitrogenous compounds directly impair essential enzymatic machinery, leading to the inhibition of DNA and RNA polymerases and the arrest of bacterial replication (Figure 4) [73]. The architectural structures formed by alkaloids in combination with other bioactive compounds also significantly restrict the biofilm formation and swarming motility that enable plant pathogens to invade the vascular system [73].

### 5.4. Sulfur-Containing Compounds

Organosulfur compounds derived from garlic (*Allium sativum*) have shown antibacterial activity against several phytopathogenic bacteria, including *Pectobacterium carotovorum* [74]. By contrast, the antibacterial activity reported for peanut shell-derived CNC/ZnO composites [75] should primarily be attributed to the ZnO nanoparticle and material-surface components—acting via mechanical abrasion and surface destabilization on the outer membrane (Figure 4)—rather than interpreted as evidence of sulfur-containing PSM activity. This example is retained here as a boundary case and is discussed further in Section 6 as a nanoparticle/material effect.

In conclusion, PSMs show strong potential as biopesticide candidates capable of controlling a broad spectrum of phytopathogenic bacteria while minimizing the risk of resistance development. However, the variable sensitivity observed across different bacterial strains underscores the need for precise dose optimization in practical applications. Future research should focus on developing species-specific biopesticide formulations tailored to target plant-pathogen systems and rigorously evaluating their environmental stability and performance in field trials. A mechanistic synthesis of the major PSM classes evaluated against phytopathogenic bacteria is presented in Table 3.

## 6. Biotechnological Applications of Plant Secondary Metabolites

The practical use of PSMs in plant disease management depends not only on their intrinsic antiviral, antifungal, or antibacterial activity but also on their stability, delivery, retention, and persistence under agricultural conditions. Many PSMs are poorly soluble in water, volatile, sensitive to oxidation or ultraviolet radiation, and susceptible to rapid loss from plant surfaces. Consequently, strong activity observed under controlled laboratory conditions does not necessarily translate into reliable performance under greenhouse, postharvest, or open-field conditions [5,76].

**Figure 5:**
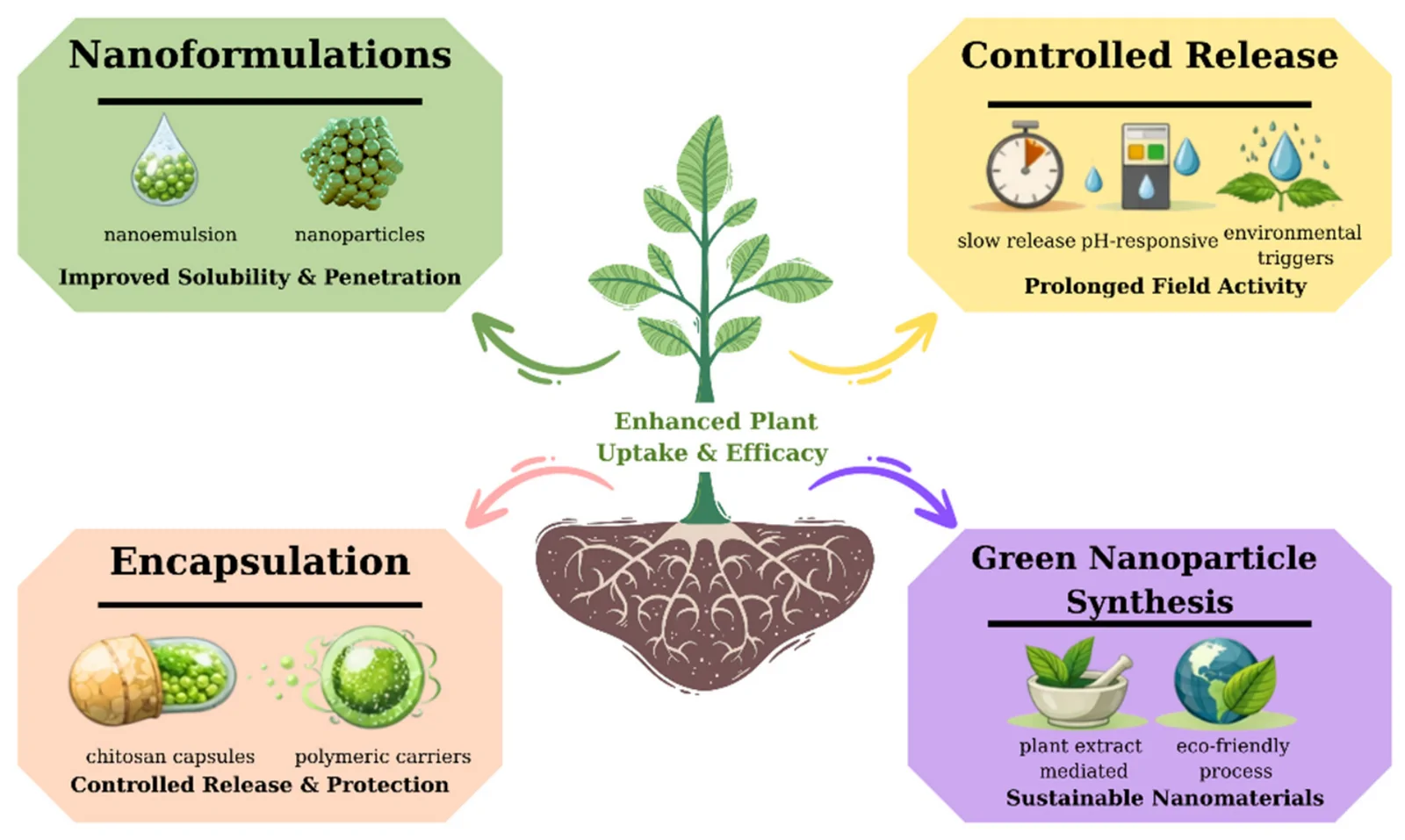
Principal biotechnological strategies used to improve the formulation and delivery of plant secondary metabolites for plant disease management. Nanoformulations can improve dispersion and surface interaction; encapsulation can protect unstable or volatile compounds; controlled-release systems regulate the temporal availability of active ingredients; and plant-mediated green synthesis uses phytochemicals in nanoparticle formation and stabilization. These approaches may overlap within a single formulation and can influence stability, retention, delivery, and biological performance. Labels describing prolonged field activity or sustainability represent intended formulation objectives; they should not be interpreted as evidence that every system has demonstrated prolonged field persistence or environmental sustainability. (Created by the authors).

Biotechnological formulation strategies have therefore been developed to improve the physicochemical and biological performance of PSMs. The approaches most frequently investigated include nanoformulations, encapsulation technologies, controlled-release systems, and plant-mediated green synthesis of nanoparticles [77]. These categories are not mutually exclusive. An encapsulated essential oil may also provide controlled release, while a green-synthesized nanoparticle can possess intrinsic antimicrobial activity in addition to carrying plant-derived surface constituents. Figure 5 summarizes the relationship among these formulation strategies.

Importantly, improved disease control after formulation should not automatically be attributed to enhanced intrinsic activity of the PSM itself. Nano- or microcarriers may improve dispersion, adhesion, surface contact, retention, or release, whereas some carrier materials, such as chitosan, may additionally influence plant defense responses [78]. Similarly, in green-synthesized metallic nanoparticles, antimicrobial activity may arise primarily from the inorganic core, with phytochemicals contributing to reduction, stabilization, surface chemistry, or additional biological interactions [79]. Distinguishing these contributions is necessary when interpreting formulation-dependent improvements in efficacy.

The following sections therefore evaluate these four technological approaches according to their formulation function, biological contribution, experimental evidence, and current limitations.

### 6.1. Nanoformulations

Polymeric nanoparticles and nanoemulsions are among the most studied nanoformulation strategies to improve the delivery of plant-derived bioactive compounds for plant protection [80,81]. Evidence available suggests that nanoformulation can significantly enhance the transport and functional availability of hydrophobic PSMs, especially those with low aqueous solubility and poor distribution on plant surfaces [82]. Due to their small size and high surface area, nanoscale carriers can improve dispersion, increase contact with biological interfaces, and promote penetration or retention of poorly water-soluble metabolites on leaves, fruits, and pathogen-associated surfaces [83]. In this regard, the use of botanical nanoformulations has been increasingly explored as a way to boost the efficacy, stability, solubility and release profile of plant-derived pesticides. Moreover, solid nanoparticles and nanoemulsions may improve wetting, adhesion to plant tissues and resistance to wash-off, critical parameters for spray-based agricultural applications [84]. The functional advantage of nanoformulations is closely related to their ability to modify the physicochemical characteristics of the active compound. Hydrophobic molecules, particularly some of the essential oil constituents (thymol, carvacrol, eugenol, citral and cinnamaldehyde), are prone to uneven distribution when applied as free forms. This limitation can be overcome by nanoscale carriers, which can stabilize the active phase in aqueous systems and increase the interfacial area between the bioactive compound and the target surface. This is especially relevant for foliar and postharvest applications where the formulation must be in contact with waxy cuticles, fruit epidermal layers, fungal hyphae, bacterial membranes or biofilm structures long enough to express biological activity.

In addition, current formulation studies further suggest that nanoformulations can be tailored to impart additional functional qualities beyond simple dispersion. For instance, Zhang et al. reported the co-assembly of nanoparticles with a plant phenolic molecule and azadirachtin, a neem pesticide. The obtained nanosystem had tiny particle size and high ζ potential, which increased the wettability, adhesion, rainfastness and photostability of the active ingredient. The formulation was also developed to be pH-responsive, stable under certain environmental conditions and disassembled in acidic or alkaline microenvironments associated with target pests. These release behaviors led to increased in vivo insecticidal efficacy compared to a traditional azadirachtin formulation [85]. This example is mainly pest-oriented but illustrates the wider formulation logic equally essential for PSM-based plant disease management. Design at the nanoscale may improve not just delivery but also environmental responsiveness and retention of the active ingredient.

Nanoformulations may also include polymeric or metallic components that can have biological activity either alone or in combination. The biogenic zinc, silver, copper and copper oxide nanoparticles produced using plant extracts have been shown to show increased antibacterial efficacy against a range of plant diseases and pest associated systems [79]. In these platforms, the nanoparticle may play more than a passive carrier. In metallic systems, it can contribute to antibacterial mechanisms such as reactive oxygen species formation, membrane rupture and enzyme inactivation, boost penetration through cuticular or microbial barriers, and improve contact with the plant surface or pathogen interface [81]. In the case of PSMs loaded, encapsulated within, or surface-associated with nanoscale carriers, the formulation may combine the intrinsic bioactivity of the metabolite with the physicochemical features of the nanocarrier [83]. Of the experimental research examined in the period 2018–2026, two formulation architectures seem particularly prevalent: nanoemulsions of essential oils and biopolymer nanoparticles. Systems based on chitosan are particularly prevalent, partly because chitosan is processable under very mild conditions and suitable with plant protection applications. Essential oil nanoemulsions, conversely, are extensively utilized for volatile and hydrophobic substances, since they enhance water dispersion and promote more interaction with plant surfaces. The majority of examined studies combined in vitro antimicrobial testing with controlled greenhouse, postharvest or seed/fruit protection assessments [86,87,88,89,90,91,92,93,94,95,96]. Under these controlled settings, nanoformulated PSMs frequently showed substantial inhibition of pathogen growth, reduced disease severity, or enhanced protection of host tissues.

Despite these encouraging results, some limitations are clear. Most of the evidence available is still from laboratory, postharvest or greenhouse systems, while full-scale field demonstrations with yield, quality, residue and long-term performance objectives are still rather scarce. This leads to a distinct translational gap between controlled experimental efficacy and real agricultural implementation. Other problems are formulation stability in storage, repeatability of particle size and surface charge, cost-effective scale-up, rainfastness in the field, and assessment of non-target safety and regulatory compatibility [87]. These challenges are especially critical since small variations in formulation chemistry can have a big impact on biological performance. Technically, ionic gelation utilizing sodium tripolyphosphate (TPP) remains one of the most prevalent ways for creating chitosan nanoparticles, while Tween-series surfactants like Tween 20 and Tween 80 are often utilized in nanoemulsions of essential oils. Reported particle or droplet sizes vary substantially with formulation composition and processing conditions, including emulsification, sonication, homogenization, and polymer-to-active ratios [86,88,95,96]. Thus, parameters such as particle size, polydispersity index, ζ-potential, encapsulation efficiency and storage stability are important to compare nanoformulations between research. However, these factors are not always given in a uniform manner across all publications, making direct comparison problematic. Nanoformulations generally offer a technically sound approach to enhancing the solubility, dispersion, adhesion and bioefficacy of PSMs. Their key contribution is the conversion of poorly soluble or unstable natural chemicals into more useful delivery systems for plant protection. However, the area is still somewhat in the formulation development and proof of concept stage. Before these technologies can be turned into reliable agricultural products, greater attention will be needed on uniform physicochemical characterisation, field-relevant testing, and performance endpoints such as disease reduction, crop quality and formulation durability. Table 4 is a summary of representative studies.

Taken together, the reviewed nanoformulation studies suggest that two recurring mechanisms largely explain the improved performance of PSM-based nanosystems: physicochemical optimization and carrier-mediated biological functionality.

#### 6.1.1. Physicochemical Optimization

The first mechanism is related to improvements in dispersion, colloidal stability, and interfacial contact. By reducing particle or droplet size, controlling the polydispersity index, and optimizing surface charge, nanoformulations can substantially modify the way hydrophobic metabolites interact with plant tissues and pathogen surfaces. This is particularly relevant for essential oil-based systems, where active compounds often show poor water compatibility and irregular distribution when applied in free form. Surfactant-stabilized nanoemulsions, including Tween-based systems, can improve wetting behavior, surface coverage, and resistance to phase separation. Similar advantages are observed in polymeric or biopolymeric nanoparticles, where the carrier structure helps retain the active compound and improves its local availability. In studies involving thymol or garlic oil nanoemulsions, these physicochemical changes have been associated with improved persistence and stronger interaction with both the plant surface and the target pathogen [86,88]. Therefore, the enhanced activity of nanoformulated PSMs cannot be explained only by the chemical nature of the metabolite; it is also strongly shaped by particle size, interfacial behavior, and formulation stability.

#### 6.1.2. Carrier-Mediated Delivery and Defense-Priming Effects

The second mechanism is more closely associated with the biological role of the carrier matrix. Biopolymer nanocarriers, particularly chitosan nanoparticles prepared through TPP-mediated ionic gelation, rarely function as passive delivery vehicles alone. In several studies, the carrier itself appears to contribute to the final biological response by improving adhesion, prolonging metabolite availability, and interacting with plant defense pathways. Chitosan is especially important in this context because it can act as an elicitor of plant immunity, partly by resembling structural fragments of fungal cell walls. As a result, chitosan-based systems may stimulate defense-related responses such as increased phenolic accumulation, activation of pathogenesis-related proteins, regulation of antioxidant enzymes, and changes in defense-associated gene expression. This carrier-mediated contribution has been reported in formulations where essential oil-loaded chitosan nanoparticles enhanced host defense responses in addition to suppressing pathogen development [97]. Thus, the performance of biopolymer-based nanoformulations should be interpreted as the outcome of both metabolite delivery and carrier-induced defense modulation, rather than as a simple consequence of nanoscale transport alone.

### 6.2. Encapsulation Technologies

Micro- and nanoencapsulation technologies constitute a major formulation strategy for improving the practical applicability of PSMs in plant disease management. These systems are closely related to nanoformulations, but their primary function is somewhat different: encapsulation is mainly designed to protect the active compound from premature degradation and to improve its retention, dispersion, and biological availability after application [84]. In encapsulated systems, the PSM is physically entrapped, surrounded, or complexed within a carrier matrix, which can reduce its direct exposure to air, moisture, ultraviolet radiation, rain, and other environmental factors that accelerate degradation or leaching [84,98]. This protective effect is particularly important for volatile essential oils, phenolic compounds, and other chemically sensitive metabolites that rapidly lose activity when applied in free form.

The reviewed experimental literature from 2018 to 2026 indicates that encapsulation has become one of the most widely used approaches for addressing the main formulation-related limitations of PSMs, including high volatility, poor aqueous solubility, and rapid environmental degradation [98,99]. By placing the bioactive compound within a defined carrier structure, encapsulation can improve aqueous compatibility and provide a more homogeneous distribution on plant tissues. This is a critical requirement for practical disease control because uneven spreading or rapid loss of the active molecule may reduce pathogen contact and weaken biological efficacy. In many cases, encapsulation also contributes to more gradual release of the active compound, although the degree of release control depends strongly on the carrier type, matrix structure, loading efficiency, and environmental conditions [99,100].

Several carrier systems have been investigated for PSM encapsulation. Chitosan nanoparticles are among the most frequently reported examples, particularly for the encapsulation of essential oils. These systems are commonly prepared through ionic gelation, often using sodium TPP as a crosslinking agent. The popularity of chitosan is related not only to its biocompatibility and film-forming capacity, but also to its biological role in plant defense activation. For example, chitosan-based encapsulation has been used to preserve essential oils and improve their performance under greenhouse conditions, including studies targeting powdery mildew in cucumber [101,102]. Such systems are relevant because they combine physical protection of the volatile active compound with the possibility of carrier-mediated defense stimulation.

Cyclodextrin inclusion complexes represent another important encapsulation approach, especially in postharvest disease management. These cyclic oligosaccharides can host hydrophobic molecules within their internal cavity, thereby improving the apparent solubility and stability of volatile phenolics, aldehydes, and essential oil constituents. In postharvest applications, this is particularly valuable because the active compound must remain available during storage without causing excessive volatilization, sensory deterioration, or rapid degradation. Cinnamaldehyde-cyclodextrin systems, for instance, have been evaluated for controlling citrus postharvest pathogens and improving the practical handling of otherwise unstable bioactives [103]. Compared with free essential oil constituents, cyclodextrin-based encapsulation offers a more controlled physicochemical environment for the active compound.

In addition to chitosan and cyclodextrins, recent studies indicate a gradual shift toward biopolymer-based and biologically derived carrier matrices such as zein, lignin, tannin-lignin hybrid systems, and related natural polymers. These materials are attractive because they are generally biodegradable, can be obtained from renewable sources, and may be compatible with sustainability-oriented agricultural formulations. Zein-based nanoparticles have been used to encapsulate essential oils or phenolic compounds [104,105], while lignin and tannin-lignin carriers have been explored for improving the stability and persistence of botanical oils [106,107,108]. In some systems, release behavior has been described using diffusion-based models, including Fickian diffusion or the Korsmeyer-Peppas equation, particularly when sustained release properties are experimentally evaluated. However, detailed release kinetics are not consistently reported across all encapsulation studies, which remains a limitation for comparing carrier performance.

The biological role of encapsulation should also be considered beyond simple protection of the active ingredient. Increasing evidence suggests that encapsulated PSM systems may act through both direct antimicrobial activity and indirect stimulation of plant defense responses. This is especially evident in chitosan-based formulations, where the carrier matrix may influence defense-related enzyme activity, phenolic accumulation, and expression of plant defense genes [101,102]. Therefore, some encapsulation systems can be described as dual-functional platforms: they stabilize and deliver the metabolite while also contributing to host defense modulation. This feature is important for plant disease management because effective protection may depend not only on suppressing pathogen growth, but also on improving the plant’s physiological resistance to infection.

Despite these advantages, encapsulation technologies are not yet fully translated into routine agricultural practice. Most available studies remain limited to in vitro assays, postharvest models, or controlled greenhouse experiments. Field-scale validation is still relatively scarce, particularly under variable environmental conditions such as rainfall, fluctuating temperature, ultraviolet exposure, and diverse soil or phyllosphere microbiota. Long-term storage stability, reproducibility of carrier preparation, cost-effective scale-up, residue behavior, and non-target safety also require further investigation before these systems can be widely commercialized. These gaps are especially relevant for regulatory assessment, since complex botanical formulations and biopolymeric carriers may require detailed characterization of composition, degradation behavior, and biological impact [96,102].

Overall, encapsulation technologies provide a strong formulation platform for improving the stability, retention, and delivery of PSMs in plant disease control. Chitosan nanoparticles, cyclodextrin inclusion complexes, zein nanoparticles, and lignin- or tannin-based carriers each address different aspects of PSM instability and application performance. While the reviewed evidence supports their potential, broader field validation and more standardized reporting of formulation parameters are still needed. Representative studies are summarized in Table 5.

Collectively, these results imply that the selection of carriers should be based on the unique formulation goal and the environment of biological application. Chitosan nanoparticle systems have a special place among the discussed encapsulation platforms. In the majority of these formulations, nanoparticles are generated by ionic gelation employing TPP as a crosslinker. Surfactants like Tween 80 are often used to stabilize the loading of volatile essential oils. Representative chitosan-based systems generally produced nanoscale particles in the order of about 100–120 nm and negative ζ-potential values; these measurements can support colloidal-stability assessment but should be interpreted together with particle size, polydispersity, medium composition, and storage behavior. In the study of Soleimani et al., the best fit for the release behavior was the Korsmeyer-Peppas model with an *n*-value of the release exponent < 0.45, which corresponds to a Fickian diffusion-controlled mechanism [101]. This is an important finding since it indicates that the carrier matrix not only retains the active ingredient but also is able to control its slow dispersion into the surrounding medium.

Another significant carrier group is the cyclodextrin inclusion complexes including β-CD, HP-β-CD and γ-CD systems, notably for postharvest disease control. Their key advantage is their capacity to increase the apparent solubility and stability of volatile aldehydes, phenolics and other hydrophobic PSMs. Cyclodextrins can create host–guest inclusion structures to decrease volatilization and to aid in the management of compounds that would otherwise be difficult to apply in aqueous systems. The complexes have shown promising efficacy in standardized postharvest storage models, in some cases reaching or exceeding the performance of traditional synthetic fungicides [110]. This makes them particularly suitable for protecting fruit and vegetables where disease control needs to be balanced against concerns about residues, sensory quality and storage stability.

Recent studies also show increasing interest in lignin- and tannin-lignin-based carriers, largely because these materials are biodegradable, renewable, and compatible with circular-economy-oriented formulation strategies. These biologically derived matrices can provide dose-reduction potential and sustained release behavior, especially for volatile botanical oils. However, their performance may depend on the interaction between the carrier and the target pathogen. For instance, carrier degradability can become a limitation when lignin-based systems are applied against fungi capable of metabolizing or disrupting lignin structures [107]. Therefore, the selection of lignin or tannin-lignin carriers should be evaluated according to both the active compound and the biological characteristics of the target pathogen.

Zein-based carriers have also shown strong potential in postharvest applications. As a plant-derived protein, zein can form stable nanoparticles and surface-deposited films that improve the retention of volatile phenolics on fruit surfaces. Their efficacy is probably associated with improved deposition, coating behavior, and prolonged local availability of the active compound at the pathogen-host interface. Controlled circumstances showed significant improvement in antifungal effectiveness due to better surface coverage and retention of thymol-loaded zein nanoparticles [105]. These results indicate that protein-based carriers may be particularly useful in situations where surface-associated protection is needed, for example in postharvest fruit systems.

Biopolymer-based encapsulation methods often provide a significant benefit over alternatives based on metal nanoparticles as they can help to mitigate some of the environmental and regulatory difficulties associated with inorganic nanomaterials. However, there is currently a lack of substantial multiseasonal and field-scale confirmation in the existing research. Most research available is limited to in vitro, postharvest or greenhouse level assessments, with only a few including agronomically important goals such as yield, crop quality, residue persistence or long-term disease suppression. This generates a substantial knowledge gap concerning actual performance. Therefore, future research should focus more on formulation stability, field stability, safety to non-targets, and reproducibility under different agricultural settings [111].

### 6.3. Controlled Release Systems

The structural instability of many PSMs has long hindered their use in agricultural disease management. Free essential oils, terpenes, phenolics and other similar lipophilic metabolites are easily altered by external stress factors such as UV light, high temperature, oxidation and changes in soil or leaf-surface pH. These conditions favor volatilization, chemical degradation and loss of biological activity upon application. Moreover, the limited water solubility of many PSMs not only limits their uniform distribution on plant surfaces but also hampers their capacity to maintain intimate contact with the target pathogen. Thus, the practical restriction is not only the instability of molecules but also the difficulty of maintaining an effective concentration of the active substance at the site of infection for a sufficient time [5,98].

Encapsulation and controlled release techniques are particularly crucial for the protection of complex lipophilic PSMs, such as essential oils, oleoresins, terpenoid-rich extracts, and volatile phenolic components. Micro- and nanoscale encapsulation may reduce evaporation of bioactive fractions, enhance chemical stability, and reduce premature degradation when exposed to the environment [112,113]. Free PSMs in traditional uses might give a rapid initial concentration when sprayed, dipped or applied to soil, but then fall rapidly due to volatilization, leaching, photodegradation, or diffusion away from the target site. Even if the chemical has shown potent activity initially, the suppression of pathogens may be suboptimal if the local concentration is below the MIC. To overcome this constraint, controlled release systems have been developed to regulate the time-dependent availability of the active cargo, prolonging plant protection and minimizing the danger of phytotoxicity due to excessive initial doses [114,115].

Generally, controlled-release systems can release active compounds through four primary mechanisms: diffusion, matrix degradation, swelling, and stimuli-responsive triggering [113,116,117]. Diffusion controlled systems involve penetration of solvent molecules into the polymeric matrix, dissolution of the active chemical, and its outward migration along a concentration gradient. This form of release is usually described by the Higuchi model which was first created for planar and homogeneous matrices as a function of the square root of time [118]. The model is simplistic, but nonetheless useful for evaluating systems where diffusion is the dominating transport process.

More complex release characteristics are generally seen in swelling, dissolving or structurally heterogeneous matrices. In such systems, the rate of release can be governed not only by diffusion but also by polymer relaxation, hydration, erosion or partial destruction of the carrier. This behavior is often described by the Korsmeyer-Peppas model, especially when the release mechanism cannot be attributed to one transport method. In this model the release exponent, *n*, indicates if the system follows Fickian diffusion, non-Fickian or anomalous transport, or more complex release combining both diffusion and matrix relaxation [119,120]. This distinction is crucial for PSM-based agricultural formulations, since the same bioactive ingredient might react quite differently depending on the carrier structure, polymer composition and environmental circumstances.

For swelling-controlled systems, hydration of the carriers leads to the relaxation of the polymer chains and the increase in the mobility of the molecule to be encapsulated. The release of cargo can be slowed down or sped up by this process, depending on the degree of swelling and the physical structure of the matrix. In degradation-controlled systems, biodegradable carriers like lignin, chitosan, alginate, or similar natural polymers are slowly degraded by microbial activity, enzymatic action, or environmental erosion [121,122]. These bio-based matrices are chosen more due to their renewable origin, biodegradability and general compatibility with sustainable agriculture principles [123]. However, their performance should be cautiously evaluated, as the release profile could be influenced by the degradation rate, soil conditions, microbial composition and pathogen type.

Stimuli-responsive systems allow for a more sophisticated, controlled release. These formulations are designed to release the active ingredients in response to specific environmental or biological stimuli, including pH changes, enzymes derived from pathogens, temperature changes, moisture, or localized infection-associated microenvironmental signals [6,124]. These systems are of particular interest in precision agriculture to avoid superfluous inputs and to increase the availability of the active ingredient at the time and site of pathogen pressure. Stimuli-responsive carriers may reduce chemical runoff and contamination of adjacent soil or water systems by minimizing premature release and reducing off-target losses. Also, controlled release of PSMs could increase bioavailability and in some cases elicit host defense or systemic resistance responses upon pathogen challenge [125].

Experimental studies published during 2018–2026 show that two main formulation approaches are dominating the controlled release strategies for PSM-based plant protection. The first one is the polymeric matrices such as chitosan, zein, lignin, alginate and related biopolymer systems. These carriers create a physical barrier around the active compound that allows gradual diffusion, swelling-mediated release or degradation-dependent liberation. The main benefits of encapsulation are increased stability, increased availability and possible reduction of dose relative to free bioactives. The second one is inclusion complexes, especially cyclodextrin based systems. Cyclodextrins are particularly useful for volatile and hydrophobic compounds. They can stabilize active molecules in host–guest structures and provide a reservoir-like release behavior. This helps to ensure availability and reduces immediate volatilization and/or degradation.

In controlled laboratory, postharvest and greenhouse conditions, controlled release systems can often suppress pathogens as well as, or better than, unencapsulated PSMs, but at reduced applied doses. However, several limitations to their commercial translation remain. Release kinetics are not always reported in sufficient detail, and many studies lack standardized parameters such as release model fit, encapsulation efficiency, loading capacity, ζ-potential, storage stability, rainfastness or ultraviolet durability. There is also little field-level evidence. In particular, there is a dearth of multiseason and multilocation trials with agronomic endpoints such as disease severity, yield, crop quality and residue behaviour. These gaps make it difficult to predict the behaviour of controlled release formulations under real agricultural conditions where temperature, rainfall, sunlight, soil chemistry and microbial communities vary greatly [109]. Table 6 provides examples of representative studies.

Table 6 shows the results of controlled release studies that suggest that the major formulation advantage of these systems in general is sustained availability and not just increased initial potency. Chitosan-, zein-, lignin- and cyclodextrin-based carriers can help to postpone premature loss of volatile or poorly soluble PSMs and sustain biologically relevant concentrations for extended periods. This is particularly the case for postharvest infections, soilborne diseases and vascular pathosystems where suppression of the pathogen generally requires prolonged exposure rather than a short-term antimicrobial peak. However, the evidence reviewed also indicates that controlled release is not always adequately characterized. Several studies describe release behavior only in general terms and do not fully model kinetics, conduct long-term stability testing, or validate under field-relevant stress conditions such as rainfall, UV exposure, and fluctuating temperature. Therefore, future controlled release studies should report release kinetics as well as biological efficacy, phytotoxicity thresholds and durability under realistic agricultural conditions.

### 6.4. Green Synthesis of Nanoparticles

The fast progress of nanotechnology-based solutions in agricultural phytopathology has led to a growing concern about the environmental load and toxicity hazards of traditional physical and chemical nanoparticle synthesis methods [129]. Many of the conventional syntheses involve the use of strong reducing agents, organic solvents, stabilizers or high energy processing conditions that may be incompatible with sustainable agricultural systems. In this context, green synthesis, using plant extracts and PSMs as reducing, capping and stabilizing agents during nanoparticle formation has emerged as a more eco-friendly bioengineering strategy [130]. Plants contain a wide variety of metabolites, such as flavonoids, phenolic acids, terpenoids, alkaloids, sugars, proteins and organic acids, which can reduce and stabilize metal ions under relatively mild conditions (room temperature, near neutral pH and ambient pressure). Nanoparticles synthesized through these routes, especially silver (Ag), copper oxide (CuO) and zinc oxide (ZnO) nanoparticles, are being studied more and more for their antimicrobial and antifungal activities against agricultural phytopathogens [129,131,132].

The green synthesis process is usually initiated by bioreduction of metal ions, where ionic metal species, usually called M*^n+^*, are reduced to lower oxidation states or zero-valent metallic forms, depending on the nanoparticle system to be synthesized [130,133]. Metabolites derived from plants can facilitate this process by electron donation, coordination interaction, and redox-active functional groups in phenolics, flavonoids, terpenoids, and proteins [130,134]. The reduced atoms or molecular clusters are nucleated and subsequently grown into more stable nanoscale structures. The growth stage is strongly dependent on the composition of extract, concentration of precursor, pH, temperature, time of reaction and competition between nucleation and aggregation. Hence, the physicochemical properties of green-synthesized nanoparticles such as particle size, morphology, surface charge and colloidal stability are highly related to the biochemical profile of the plant extract used in the synthesis.

After nucleation and growth, phytochemical constituents are attached to the high-energy surfaces of the nanoparticle core through coordinate bonding, electrostatic interaction, hydrogen bonding or other weak intermolecular interactions. This is often referred to as capping or stabilization. It creates an organic layer from the plant to the nanoparticle surface to avoid excessive aggregation and helps in colloidal stability [133]. In addition, this phytochemical layer associated with the surface may also influence biological interactions with plant tissues and microbial cells. Therefore, the surface chemistry and bio-functional behavior of green synthesized particles might be different from nanoparticles synthesized using conventional chemical stabilizers. Nevertheless, this benefit should be used with care as the biological performance of such nanoparticles depends not only on the green synthesis pathway but also on the particle size, surface charge, crystallinity, extract composition, and dose [135,136].

The antimicrobial activity of green-synthesized nanoparticles is generally attributed to the interaction between the inorganic nanoparticle core and the plant-derived coating on its surface. The high surface to volume ratio of the nanoparticle core provides close contact with pathogen cell walls and membranes. This interaction in metal and metal oxide nanoparticles can facilitate the generation of reactive oxygen species (ROS), disruption of membranes, leakage of intracellular components, protein oxidation, enzyme inhibition, and damage to DNA or other critical cellular components [137]. These mechanisms are particularly important for phytopathogenic bacteria and fungi, in which the loss of membrane integrity and oxidative stress can quickly decrease the viability of the pathogen. Under suitable environmental conditions, photocatalytic and catalytic properties may also be involved in microbial inhibition of CuO and ZnO nanoparticles.

The capping layer formed from plants may add additional biological functions. At the same time, the phytochemicals on the surface of the nanoparticle can affect the interaction of the nanoparticle with the pathogen and in some circumstances directly contribute to the antimicrobial activity. Some studies have also shown that green-synthesized nanoparticles can impact plant defensive responses. This effect is associated with increased activity of defense-related enzymes, such as phenylalanine ammonia-lyase (PAL) and peroxidase (POD), and altered expression of systemic acquired resistance (SAR)-related genes [125,138]. In this context, green-synthesized nanoparticles can act as direct antibacterial agents and as plant defence-priming agents. Their effects on seed germination, seedling vigor and stress tolerance further suggest their potential function in integrated plant protection; however, dose optimization is needed to minimize phytotoxicity.

In addition, the green synthesis of nanoparticles is also important in the postharvest disease management and protective packaging applications. Their use in smart nanocoatings, edible films or postharvest protective coatings could be useful to manage gas permeability, delaying the ethylene build up, lowering the microbial deterioration and extending the shelf life. Such applications are especially significant in fruits and vegetables where disease management must be performed without loss of food quality, consumer safety or sensory qualities. Green nanoparticle-based coatings can assist in the development of safer postharvest management systems by minimizing the need for conventional chemical preservatives and synthetic fungicides [139]. However, their actual application demands careful examination of migratory behavior, residual permanence, safety for food contact and long-term environmental fate.

PSMs in various systems play a closely related dual functional role. They are primarily involved in bioreduction, which converts metal ions into nanoparticle nuclei and/or lower valence state metal species through the action of redox-active functional groups. Secondly, they are involved in capping and stabilization by producing a protective organic coating around the nanoparticle core, limiting aggregation and modulating interactions with phytopathogens. This dual role sets plant-mediated synthesis apart from many conventional chemical approaches and also accounts for the fact that the biological activity of green-synthesized nanoparticles cannot be completely attributed to the inorganic core. Rather their performance is a joint contribution of nanoparticle physicochemistry, phytochemical surface composition and pathogen specific susceptibility.

Overall, green synthesis is a potential approach to produce antimicrobial nanoparticles using plant-derived metabolites and very mild reaction conditions. Plant-mediated synthesis of silver, copper oxide and zinc oxide nanoparticles have shown significant promise against fungal and bacterial phytopathogens. However, substantial translational obstacles remain in the discipline. Open issues include standardization of plant extract content, reproducibility of nanoparticle properties, dose-dependent phytotoxicity, non-target effects, environmental persistence and regulatory classification. Thus, green-synthesized nanoparticles, although a viable platform for sustainable plant disease management, should be supplemented by thorough physicochemical characterization, field-relevant efficacy testing, and safety assessment for agricultural adoption. Representative studies are summarized in Table 7.

Studies suggest that green-synthesized nanoparticles can have antibacterial action via nanoparticle-mediated effects and plant-derived surface chemistry as shown in Table 7. Plant extracts are mostly used as reducing and stabilizing agents and the obtained Ag, CuO or ZnO nanoparticles suppress the pathogen through disturbing the membrane, causing oxidative stress and interfering with some important cell activities. This dual contribution makes green synthesis interesting for the sustainable management of plant diseases. However, more uniform reporting is still required in the field. The data on particle size, morphology, ζ-potential and polydispersity, extract composition and dose-dependent phytotoxicity are not always reported in a uniform manner between investigations. Also, whereas many systems exhibit excellent activity in vitro, greenhouse or postharvest testing, their environmental fate, non-target impacts and long-term field performance are yet weakly defined. Therefore, green synthesis should not only be considered as a sustainable production pathway but also as a formulation method that needs comprehensive physicochemical, biological and safety evaluation.

## 7. Challenges and Limitations

The main advantage of PSM-based disease control is mechanistic breadth combined with renewable sourcing. The main disadvantages are chemical variability, rapid environmental loss, uncertain dose–response relationships, and limited evidence from open-field conditions. These constraints should be treated as linked development problems rather than as a generic list of limitations.

### 7.1. Low Physicochemical Stability and Environmental Degradation

A primary technical constraint limiting the commercialization of botanical biopesticides is the inherent low stability of their bioactive components. Unlike synthetic counterparts, molecules such as volatile terpenes, essential oils, and phenolic compounds are highly susceptible to rapid oxidation, thermal decomposition, and volatilization [148,149]. When exposed to open agricultural environments, solar UV radiation and fluctuations in ambient temperature drastically accelerate the degradation of these active ingredients, significantly shortening their residual activity on plant surfaces [148,150]. This rapid environmental dissipation necessitates high-frequency reapplications, which diminishes economic viability and limits long-term protection against persistent phytopathogens [151]. Formulation should therefore be justified by a measurable increase in stability or protection time, rather than only by higher initial inhibition under in vitro conditions.

### 7.2. Field Application and Delivery Dynamics

The transition from in vitro efficacy to in vivo field success represents a major hurdle in bio-agrochemical engineering. Many potent antimicrobial PSMs possess low aqueous solubility and limited persistence on plant surfaces, which can reduce effective exposure at infection sites [152]. Furthermore, standard agricultural spraying equipment often fails to deliver these hydrophobic and sensitive formulations uniformly across dense crop canopies without inducing rapid wash-off during rain events [151,153]. Although advanced biotechnological strategies such as nanoencapsulation, polymeric nanoparticles, and nanoemulsions have emerged to shield metabolites from premature degradation and improve foliar adhesion, scaling up these green nanotechnologies to industrial production capacities remains cost-prohibitive for broad-acre farming [149,153]. Although nanoencapsulation and polymeric carriers may improve adhesion and persistence, the additional manufacturing cost and potential carrier-related environmental effects should be evaluated against the magnitude and duration of the biological benefit achieved. Field studies should therefore report application efficiency and formulation durability alongside disease suppression.

### 7.3. Chemical Standardization Inconsistencies

The structural complexity and multi-component nature of plant extracts pose a severe challenge to quality control and standardization. The qualitative and quantitative profile of secondary metabolites within a single plant species is highly dynamic, heavily influenced by geographical origin, climate conditions, soil microbiomes, harvesting seasons, and extraction methodologies [150,154]. This inherent biochemical variability makes it exceptionally difficult to guarantee a uniform concentration of the active marker compounds across different production batches [154,155]. Consequently, inconsistent field efficacy is frequently observed, undermining farmers’ trust in botanicals compared to the predictable performance of synthetic agrochemicals [155]. Without marker-compound specifications and batch-level chemical analysis, variation in efficacy cannot be reliably separated from true differences in pathogen susceptibility. Minimum reporting should therefore include botanical identity, extraction conditions, chemical fingerprint, active-marker range, and stability during storage.

### 7.4. Regulatory Frameworks and Market Approval Hurdles

Even when technical and formulation barriers are overcome, the commercialization of PSM-based plant protectants is severely bottlenecked by stringent and often mismatched regulatory pathways. Current authorization frameworks globally (such as EFSA in Europe and EPA in the United States) were historically designed for single-molecule synthetic chemicals rather than complex, multi-component botanical mixtures [156]. Regulatory bodies demand exhaustive toxicological, ecotoxicological, and compositional profiling for every single major constituent within an extract. Achieving this level of characterization for complex essential oils or crude extracts requires substantial financial investment and prolonged registration timelines, which often acts as a major barrier for small- and medium-sized biotechnology enterprises seeking market entry [157]. A more realistic route toward registration is therefore to narrow extract composition where possible, define active markers and relevant impurities, establish reproducible manufacturing specifications, and ensure that efficacy claims remain proportional to the biological evidence generated.

## 8. Future Perspectives

### 8.1. Priority 1: Field Validation and Comparable Performance Endpoints

As emphasized in Section 6, the fact that nano-biopesticides exhibit near 100% efficacy under controlled laboratory conditions does not imply that they will yield the same results in the field. To translate successful laboratory-scale demonstrations into reliable field products, fundamental knowledge gaps-such as decoupling matrix-dependent environmental behavior from the effects of carrier-induced hazards on non-target organisms must be bridged [158]. In the future, closing this gap will pave the way for large-scale and multicenter open-field trials. At this point, artificial intelligence-supported simulation models have critical roles. Predicting site-specific dynamic variables, such as wind dispersion, rain erosion, and soil microbiota interactions, will provide the opportunity to foresee the extent to which the efficacy observed under laboratory conditions can be transferred to real field conditions.

Future studies should prioritize replicated field trials using comparable endpoints, including disease severity, yield, crop quality, formulation persistence, and phytotoxicity. Without these standardized outcomes, improvements observed under controlled conditions will remain difficult to compare across formulations and crops.

### 8.2. Priority 2: Dual-Action Formulations Combining Delivery and Host Defense

As demonstrated in the case of chitosan, carrier matrices should no longer be viewed merely as passive coatings. It has been shown that when chitosan is applied as a foliar spray or soil amendment, it triggers systemic acquired resistance in the plant and regulates the expression of phenylalanine ammonia-lyase and pathogenesis-related genes [159]. Future smart formulations can be designed as multifunctional by building upon this principle. The efficacies of PSMs, stabilized through encapsulation, will be rendered more sustainable. Since low molecular weight chitosan is known to be particularly effective in triggering plant immune responses, dual-action systems will achieve high synergy, encompassing both direct biocidal and indirect immune-based defence [160]. The value of such dual-action systems should, however, be demonstrated by appropriate controls that separate the contribution of the PSM from that of the carrier. This distinction is necessary to determine whether improved disease control results from enhanced delivery, host-defense activation, direct antimicrobial activity, or a combination of these effects.

### 8.3. Priority 3: Triggered Release Systems with Agronomic Relevance

Smart release systems triggered by pathogen enzymes and pH constitute an agronomically critical area of application. The pH fluctuations arising from various pathogens in the production environment offer a natural initiator for designs that can precisely control the release kinetics of the active ingredient [100]. Research on these stimulus-responsive carriers has also expanded in recent years into the fields of material designs sensitive to environmental variables such as light, temperature, pH, and enzymes [6]. Looking to the future, integration of these nanoformulations with Internet of Things (IoT)-based agricultural sensor systems provides a useful area for investigation. Volatile organic compounds released by plants at an early stage against pathogen or pest attacks constitute a class of non-invasive biomarkers. This capability will enable the spraying of micro-dosage triggering agents onto that specific area upon the first chemical signal detected by sensors, and the just-in-time, pinpoint release of the metabolites within the carrier. Priority should be given to trigger mechanisms that correspond to measurable infection-associated changes and provide a demonstrable reduction in active-ingredient use or extension of protection compared with conventional release systems. Technological complexity alone should not be considered an agronomic advantage.

Taken together, the immediate research priority is not the development of increasingly complex formulations, but the generation of reproducible evidence linking formulation design to measurable agricultural benefit. Field validation, mechanistic separation of carrier and metabolite effects, and release systems with demonstrable agronomic value should therefore precede further increases in technological complexity.

## 9. Conclusions

PSMs offer multiple avenues for controlling viral, fungal and bacterial diseases of plants. Their protective effects may result from direct activity against pathogens or virions, modulation of host defense responses, or both. However, the use of these compounds in practice is limited because of their volatility, poor water solubility, chemical instability, variable extract composition and inconsistent performance under field conditions. Some of these limitations can be overcome by nanoformulations, encapsulation technologies, controlled-release systems and green-synthesized nanoparticles that enhance stability, retention, delivery or local availability of active compounds. However, formulation-dependent improvements should be viewed with caution as carriers and nanoparticle components may also contribute directly to the biological response observed. To date the evidence is largely based on laboratory, post-harvest and greenhouse studies. Thus, further progress relies on standardized chemical characterization, mechanistic separation of metabolite and carrier effects, replicated field validation, dose optimization, ecotoxicological assessment and comparison using agronomically relevant endpoints. The chances of PSM-based systems becoming reliable components of sustainable plant protection are greatest when biological activity is coupled with reproducible formulation performance and evidence generated under realistic agricultural conditions.

## Figures and Tables

**Table 1:** Mechanistic synthesis of the major PSM classes evaluated against plant viruses. Study-level dose and assay details were condensed to emphasize direct antiviral effects, host-mediated responses, and biological validation.

PSM Class	Representative Compounds or Sources	Representative Targets	Mechanistic Synthesis and Biological Evidence	References
Terpenoids	Rosemary, thyme, oregano, yarrow EO; limonoids	TMV, ToLCNDV, CMV, PVY	Direct inactivation or replication interference; PAL/SA-associated host-defense responses; reduced vector acquisition or transmission	[13,14,15,16,17]
Phenolics	Resveratrol derivatives, quercetin, catechinic acid	TMV, ToBRFV	Structure-activity optimization; suppression of Hsp70-associated viral processes; coat-protein disruption and loss of viral infectivity	[18,19,20,21]
Nitrogen-containing compounds	Diterpenoid and isoquinoline alkaloids, nicoisoindoles, berberine	TMV, TSWV	Virus-particle disruption, replication inhibition, and host-defense activation; evidence derived mainly from controlled in planta assays	[22,23,24,25]
Sulfur-containing compounds	Garlic extracts, camalexin derivatives	HSVd, grapevine viruses, ToBRFV, TMV	Suppression of viral/viroid titer; virion disassembly or genome disruption; dose-dependent efficacy and host tolerance	[26,27,28,29]

Detailed study-level information on the antiviral activity of individual PSMs is provided in Supplementary Table S1.

**Table 2:** Mechanistic synthesis of the major PSM classes evaluated against phytopathogenic fungi.

PSM Class	Representative Compounds or Sources	Representative Targets	Mechanistic Synthesis and Biological Evidence	References
Phenolic-rich compounds/extracts	Clove-derived eugenol and eugenyl acetate	*Colletotrichum*, *Botrytis*, *Monilinia* spp.	Strong inhibition of fungal growth and disease development; membrane-disruptive and lipid-metabolism effects are mechanistically plausible for eugenol-rich preparations, although these mechanisms were not directly measured in all studies. Host physiological responses have also been reported.	[30,31,32,33]
Terpenoids	Thymol, carvacrol, citral, limonene, geraniol, myrcene, humulene	*Botrytis*, *Alternaria*, *Rhizoctonia*, *Cercospora*, *Fusarium*, *Penicillium* spp.	Membrane permeabilization, hyphal damage, altered ergosterol-dependent organization, and oxidative imbalance. Some preparations also induce PAL, CHI, PPO, GLU, or callose-associated host responses.	[34,35,36,37,38,39,40,41,42,43,44,45,46,47,53]
Nitrogen-containing metabolites	Quinolizidine alkaloids, sparteine, lupanine, piperine, capsaicin	*Fusarium*, *Aspergillus* spp.	Membrane and metabolic disruption, hyphal deformation, altered oxidative responses, and suppression of secondary metabolism, including aflatoxin production.	[48,49,50]
Sulfur-containing metabolites	Onion-derived PTS and PTSO	*Verticillium dahliae* and other soil-borne fungi	Volatile fumigant activity can strongly reduce fungal populations and disease severity. Biological efficacy and crop tolerance are dose-dependent.	[51,52]

Detailed study-level information is provided in Supplementary Table S2.

**Table 3:** Mechanistic synthesis of the major plant secondary metabolite classes evaluated against phytopathogenic bacteria.

PSM Class	Representative Compounds/Sources	Representative Targets	Mechanistic Synthesis and Biological Evidence	References
**Phenolics**	Cinnamaldehyde, hydroxytyrosol, rosmarinic acid, chlorogenic acid, catechol	*Clavibacter*, *Xanthomonas*, *Pseudomonas* and related bacteria	Membrane permeabilization, ATP loss, enzyme inhibition and disruption of cellular homeostasis; selected in planta protective effects have also been reported.	[54,55,56,57,58,59,60,61,62,63]
**Terpenoids**	Thymol, carvacrol, terpinen-4-ol, 1,8-cineole, citral, menthol, nepetalactone	*Agrobacterium*, *Pseudomonas*, *Xanthomonas* and other bacteria	Membrane and respiratory disruption, metabolic interference and inhibition of virulence-related behavior; efficacy varies with chemotype and constituent interactions.	[59,64,65,66,67,68,69]
**Nitrogen-containing and peptide-based bioactives**	Antimicrobial peptides, protein hydrolysates, and alkaloid-rich extracts	*Xanthomonas*, *Pectobacterium* and other bacterial models	Metabolic inhibition, ATP-dependent replication/virulence effects, and reduced biofilm or motility; agricultural relevance depends strongly on the experimental model.	[71,72,73]
**Sulfur-containing compounds**	Garlic-derived organosulfur compounds	*Pectobacterium* and related phytopathogens	Garlic-derived organosulfur compounds show antibacterial activity through reactive sulfur chemistry; the CNC/ZnO example is a boundary case showing that material effects must be separated from intrinsic PSM activity.	[74,75]

Detailed study-level information is provided in Supplementary Table S3.

**Table 4:** Representative nanoformulation strategies for improving the delivery and bioefficacy of PSMs against phytopathogens.

PSM/Nanoformulation	Crop-Pathogen	Key Outcome	References
Thymol O/W nanoemulsion	Soybean-*Xanthomonas axonopodis* pv. *glycines*	Complete in vitro inhibition; disease control efficacy up to 95.4% in pot experiments	[86]
Garlic oil nanoemulsion	Citrus-*Penicillium italicum*	~300-fold reduction in MIC compared with free garlic oil; effective postharvest inhibition	[88]
Green tea essential oil nanoemulsion	Rice-*Magnaporthe oryzae*	Reduced rice blast lesions; performance reported as comparable to tricyclazole	[89]
Thymol nanoemulsion	Tomato-*Botrytis cinerea*	Complete mycelial inhibition at 0.05%; gray-mold lesion reduction up to 78.5%	[91]
*Nigella sativa* oil nanoemulsion	Maize-*Penicillium verrucosum*	Optimized formulations achieved complete fungal inhibition	[92]
Cinnamaldehyde-annonaceous acetogenin nanoemulsion	*Colletotrichum* sp.; *Fusarium oxysporum*	86.7% and 93.2% in vitro inhibition, respectively	[93]
Plant essential oil nanoemulsions	Cucumber-*Podosphaera fusca*	Greenhouse disease severity reduced by up to 54.1%; preventive application reached 72.4% reduction	[95]
*Sideritis serrata* extract-loaded chitosan nanoparticles	Wheat/multiple fungal pathogens	Up to 156,140-fold in vitro enhancement against A. brasiliensis relative to the free extract, with ~95% seed germination	[96]

Detailed study-level information is provided in Supplementary Table S4.

**Table 5:** Encapsulation-based delivery systems for enhancing the stability, retention, and antimicrobial performance of PSMs in plant disease control.

PSM/Encapsulation System	Crop-Pathogen	Key Outcome	References
*Cymbopogon martinii* EO-chitosan nanoparticles	*Fusarium graminearum*	Complete suppression of fungal growth and mycotoxins at 700 ppm, versus 900 ppm for free EO	[109]
Thymol-HP-β-cyclodextrin complex	Lemon-*Geotrichum citri-aurantii*	Reduced sour-rot incidence and severity; 50 mM treatment showed efficacy comparable to propiconazole	[110]
*Satureja montana* EO-zein nanoparticles	Tomato-*Xanthomonas euvesicatoria*	Substantially reduced bacterial load in plants and supported plant defense responses	[104]
*Thymus vulgaris* EO-chitosan nanoparticles	Tomato-*Fusarium solani*	~89.6% fungal growth reduction in vitro and marked suppression of disease severity in planta	[97]
Cinnamaldehyde-γ-cyclodextrin complex	Citrus-*Penicillium digitatum*	Disease incidence after 30 d decreased to ~6.7%, compared with ~22.7% in the control	[103]
*Thymus serpyllum* EO-lignin nanoparticles	Black pine-*Phytophthora cactorum*	Sustained EO release and near-complete protection against seedling mortality	[107]
Neem oil-tannin/lignin nanocapsules	Grapevine pathogens	Encapsulation enhanced antifungal activity against *Phaeoacremonium minimum* by >1–2 orders of magnitude	[106]
Thymol-zein nanoparticles	Banana-*Colletotrichum musae*	Approximately 200–300-fold enhancement of in vitro antifungal activity and delayed symptom development	[105]

Detailed study-level information is provided in Supplementary Table S5.

**Table 6:** Controlled release systems for sustained and regulated delivery of PSMs in plant protection application.

PSM/Controlled-Release System	Crop-Pathogen	Key Outcome	References
*Satureja montana* EO-zein nanoparticles	Tomato-*Xanthomonas euvesicatoria*	EO-zein nanoparticles reduced bacterial load by ~38-fold and provided stable carrier performance	[104]
*Athyrium sinense* EO-chitosan nanoparticles	Chinese cabbage-*Pectobacterium carotovorum* subsp. *carotovorum*	Fickian release behavior; preventive and therapeutic efficacy of 82.5% and 80.4%, respectively	[126]
Carvacrol-β-cyclodextrin inclusion complex	Shatangju mandarin-*Penicillium digitatum*	Reduced disease incidence and severity compared with free carvacrol under fumigation conditions	[127]
Celery seed EO-chitosan nanoparticles	Cucumber-*Podosphaera fusca*	Fickian release described by Korsmeyer-Peppas model; ~75% reduction in powdery mildew severity at 200 μg/mL	[101]
Spinach seed EO-chitosan nanoparticles	Cucumber-*Podosphaera fusca*	Controlled release combined with disease suppression and induction of host defense responses	[102]
(E)-2-Octenal-β-cyclodextrin inclusion complex	Citrus-prochloraz-resistant *Penicillium italicum*	Stabilized volatile PSM and reduced blue-mold development against a fungicide-resistant strain	[128]

Detailed study-level information is provided in Supplementary Table S6.

**Table 7:** Green synthesis of metal and metal oxide nanoparticles using PSMs for sustainable plant disease management.

Plant-Mediated Nanoparticle	Crop-Pathogen	Key Outcome	References
Tomato leaf extract-mediated AgNPs entrapped in chitosan	Tomato-*Ralstonia solanacearum*	Reduced bacterial wilt incidence in a partially resistant tomato genotype	[140]
Plant extract-mediated AgNPs	Rice-*Xanthomonas oryzae* pv. *oryzae*	Strong antibacterial activity at 20 μg/mL, with inhibition of biofilm formation and motility	[141]
*Mahonia fortunei* extract-mediated AgNPs	Chinese cabbage-*Pectobacterium carotovorum*	MIC/MBC of 8 μg/mL and effective suppression of soft rot in explants	[142]
Neem leaf extract-mediated AgNPs	Tomato-*Alternaria solani*	Disease severity and incidence reduced by ~73% and ~69%, respectively	[143]
Oak fruit exudate-mediated AgNPs	Vegetables-*Pectobacterium carotovorum* subsp. *carotovorum*	Reduced postharvest soft-rot infection across multiple vegetable hosts	[144]
Coffee extract/EGCG-mediated CuO nanoparticles	Chickpea-*Fusarium oxysporum* f. sp. *ciceris*	Low-dose treatment reduced wilt incidence by ~74.5%	[145]
*Quercus incana* leaf extract-mediated AgNPs	Tomato-*Alternaria solani*	~98% in vitro inhibition and ~93% inhibition in greenhouse leaf assays	[146]
*Zizyphus spina-christi* leaf extract-mediated CuO nanoparticles	Tomato-*Fusarium solani*	Greenhouse disease reduction of 72–88.6%, with efficacy comparable to or exceeding reference treatments	[147]

Detailed study-level information is provided in Supplementary Table S7.

## Data Availability

No new data were created or analyzed in this study. Data sharing is not applicable to this article. All information discussed in this article is available from the cited literature.

## Supplementary Materials

The following supporting information can be downloaded at: https://www.mdpi.com/article/10.3390/life16101646/s1. Table S1: Detailed study-level evidence on the antiviral activity of plant secondary metabolites against plant viruses and viroids; Table S2: Detailed study-level evidence on the antifungal activity of plant secondary metabolites against phytopathogenic fungi; Table S3: Detailed study-level evidence on the antibacterial activity of plant secondary metabolites against phytopathogenic bacteria; Table S4: Detailed characteristics and biological performance of nanoformulations incorporating plant secondary metabolites for plant disease management; Table S5: Detailed overview of encapsulation technologies for plant secondary metabolites used in plant disease management; Table S6: Detailed overview of controlled-release systems containing plant secondary metabolites for plant disease management; Table S7: Detailed overview of green-synthesized nanoparticles obtained using plant-derived reducing and stabilizing agents for plant disease management.
