# Supplementary material for: Plant Secondary Metabolites for the Control of Plant Diseases: Antiviral, Antifungal, and Antibacterial Activities and Their Biotechnological Applications
Source: Life (Basel). 2026 Sep 30;16(10):1646. doi: 10.3390/life16101646 (PMC13706413; doi:10.3390/life16101646)
Supplement: Supplementary file 1 [file life-16-01646-s001.zip › life-4547720-supplementary.pdf]

Plant Secondary Metabolites for the Control of Plant Diseases: Antiviral, Antifungal, and Antibacterial Activities and Their Biotechnological Applications

Ece Basak [1,†], Bikem Ulusal [1,†], Ayse Betul Bingol [1,†], Musa Turker [1], Cem Bulent Ustundag [1,2] and Ilknur Yilmaz [3,*]

[1] Department of Bioengineering, Faculty of Chemistry and Metallurgy, Yildiz Technical University, Istanbul 34220, Turkey; ecebasakbio@gmail.com (E.B.); bikem.ulusal@std.yildiz.edu.tr (B.U.); betul.bingol@std.yildiz.edu.tr (A.B.B.); mturker@yildiz.edu.tr (M.T.); cbustun@yildiz.edu.tr (C.B.U.)
[2] Health Biotechnology Joint Research and Application Center of Excellence, Yildiz Technical University, Istanbul 34220, Turkey
[3] Department of Molecular Biology and Genetics, Faculty of Engineering and Natural Sciences, İstanbul Atlas University, Istanbul 34408, Turkey
* Correspondence: ilknur.yilmaz@atlas.edu.tr
† These authors contributed equally to this work.

Supporting Information

This Supporting Information provides the extended datasets underlying the summary tables presented in Sections 3-6 of the main manuscript. The tables contain detailed information on antiviral, antifungal, and antibacterial activities, formulation characteristics, encapsulation approaches, controlled-release systems, and plant-mediated nanoparticle synthesis, including physicochemical parameters, biological targets, experimental conditions, and reported efficacy.

Table S1. Detailed study-level evidence on the antiviral activity of plant secondary metabolites against plant viruses and viroids.

Secondary Metabolite	Extract Source	Target Pathogen	Mode of Action and Control Efficiency	Source
Findlayine A, Dendrofindline B	*Dendrobium findlayanum*	Tobacco mosaic virus (TMV)	Alleviation of TMV-induced protoplast damage; modulation of *PAL*, *POD*, and *SOD* activities; downregulation of TMV coat-protein expression/aggregation; dendrofindline B inhibited infection by 38.6%.	[1]
Alpha-Terpineol, Isoborneol, Camphor	Rosemary (*Salvia rosmarinus*)	Tobacco mosaic virus (TMV)	Curative, protective, and direct-inactivation effects reached 76.3%, 70.7%, and 62.8%, respectively, at 300 μg/mL; molecular docking suggested interactions with TMV protein targets.	[2]
Chelerythrine, Chelidonine, Sanguinarine	Greater celandine (*Chelidonium majus*)	Tobacco mosaic virus (TMV)	Chelerythrine showed 72.67% inactivation and 77.52% proliferation inhibition at 0.5 mg/mL; chelidonine provided up to 64.45% inhibition through an induced-resistance mode of action at 0.1 mg/mL.	[3]
Nicoisoindole A-D (Nicoisoindole D)	Tobacco stalk (*Nicotiana tabacum*)	Tobacco mosaic virus (TMV)	Half-leaf anti-TMV inhibition reached 67.8% at 20 μM; nicoisoindole D showed 62.6% protective efficacy in host plants.	[4]
Carvacrol, Thymol, Verbenone	Thyme, oregano, and rosemary hydrosol	ZYMV and Tomato leaf curl New Delhi virus	O. *vulgare* and T. *vulgaris* preparations showed no efficacy against ZYMV but reduced ToLCNDV infection or viral load; selected treatments increased *PAL* expression, and rosemary hydrosol supported symptom-free development in therapeutic application.	[5]
Munronin T, Munronin U	*Munronia henryi*	Tobacco mosaic virus (TMV)	Protective inhibition ranged from 70.5% to 81.3%; the most active limonoid increased salicylic-acid-associated defense responses and suppressed host *Hsp70* and TMV coat-protein expression.	[6]
Resveratrol ester derivatives	*Polygonum cuspidatum*	Tobacco mosaic virus (TMV)	Ester-modified resveratrol derivatives showed enhanced anti-TMV inactivation and protective activity, with several derivatives outperforming ribavirin.	[7]
Chlorogenic acid, 1,8-cineole	Yarrow (*Achillea millefolium*)	Cucumber mosaic virus (CMV)	A 4% yarrow essential-oil nanoemulsion applied after infection inhibited local lesions by 59.82% and reduced CMV accumulation by 63.68%; *PAL*, total phenolics, *APX, POD*, and *CAT* responses were increased.	[8]
Thyme essential oil, Chitosan-Lecithin	Thyme (*Thymus vulgaris*)	Potato virus Y (PVY) and aphid vector	Suppression of aphid virus acquisition reached 57.5%; the chitosan-lecithin-thyme-oil formulation reduced transmission by 50.0% and enhanced antioxidant responses and *PAL-1/PR-2* expression.	[9]

Crude garlic extract (Organosulfur)	Garlic (*Allium sativum*)	GLRaV-2, GFkV, GVA, GFLV, GRSPaV	Garlic extract enhanced *VvPR1*, *VvPR4*, and *VvPR10* expression and antioxidant-enzyme responses; field viral loads were reduced to 15-42% of control levels, depending on the virus.	[10]
Fructosyl sulfur derivatives (Fru-GSAC)	Vessalico garlic (*Allium sativum*)	Tomato brown rugose fruit virus (ToBRFV)	Garlic extracts deactivated ToBRFV infectivity, consistent with coat-protein disassembly; sulfur compounds characteristic of aged extracts showed strong interactions with the ToBRFV coat-protein binding site.	[11]
Camalexin derivatives	*Arabidopsis and Camelina phytoalexin*	Tobacco mosaic virus (TMV)	Camalexin derivative 5a caused 20S coat-protein disk fusion/disintegration, interfering with TMV particle assembly; several derivatives matched or exceeded ningnanmycin in antiviral assays.	[12]
Nanoliposomal quercetin (H-TQ-NPs)	Natural flavonol	Tobacco mosaic virus (TMV)	Nanoliposomal quercetin targeted host Hsp70-associated processes, reduced TMV coat-protein accumulation, and improved field disease-control efficacy relative to free quercetin.	[13]
Resveratrol alkyl sulfonate derivatives	Synthesized phytoalexin	Tobacco mosaic virus (TMV)	Resveratrol sulfonate derivatives showed optimized anti-TMV activity; the best compounds achieved about 59% inactivation together with strong protective and curative efficacy.	[14]
Berberine	*Corydalis saxicola* Bunting	Tomato spotted wilt orthotospovirus (TSWV)	Berberine enhanced *GSTAU25*-associated resistance and suppressed viral NSs/NSm expression; protective efficacy reached 62%, and anti-TSWV activity exceeded 76% at 200 µg/mL.	[15]
Catechinic acid, 4-hydroxybenzoic acid	*Combretum micranthum (*Autoxidized*)*	Tomato brown rugose fruit virus (ToBRFV)	Autoxidized extract components rapidly deactivated ToBRFV; treated inoculum lost infectivity, no viral RNA was detected in plants 35 days later, and docking supported coat-protein interactions.	[16]
Alliin, Allicin, and Ajoene architectures	Garlic extract (*Allium sativum*)	Hop stunt viroid (HSVd)	Garlic extract reduced HSVd accumulation by 74.45%; Nanopore sequencing indicated compromised HSVd genome integrity, while higher extract concentrations caused contamination and plant damage.	[17]

Table S2. Detailed study-level evidence on the antifungal activity of plant secondary metabolites against phytopathogenic fungi.

Secondary Metabolite	Extract Source	Target Pathogen	Mode of Action and Control Efficiency	Source
Eugenol, Eugenyl acetate	Clove (*Syzygium aromaticum*)	Apple / *Monilinia spp.*	Strong *in vitro* inhibition of Monilinia spp.; at 100 µL/mL, clove essential oil reduced disease severity to 0% in inoculated apples under the tested conditions.	[18,19]
Carvacrol	Greek oregano (*Origanum heracleoticum*)	Strawberry / *Botrytis cinerea*	Plasma-membrane damage with leakage of nucleic acids, proteins, and sugars; O. *heracleoticum* vapor reduced strawberry gray-mold disease index by approximately 57.7%.	[20]
Thymol, p-Cymene	Common thyme (*Thymus vulgaris*)	Spinach / *Fusarium oxysporum*	Seed treatment with thyme essential oil reduced Fusarium wilt disease by approximately 54% under greenhouse conditions.	[21]
Dittany essential oil	Dittany (*Origanum dictamnus*)	Pepper / *Botrytis cinerea*	Vapour and dipping treatments reduced *Botrytis cinerea* lesion development; residual protection reached approximately 44.7% under the tested pre-exposure conditions.	[22]
Citral (Geranial, Neral)	Lemongrass (*Cymbopogon citratus*)	Bean / *Colletotrichum lindemuthianum*	Lemongrass essential oil reduced bean anthracnose severity by approximately 29% and increased CHI and PPO defense-related responses.	[23]
D-Limonene, Beta-Selinene	Celery seed (*Apium graveolens)*	Cucumber / Pathogenic Fungi	Increased phenolic/flavonoid and defense-enzyme responses; greenhouse disease severity was reduced by about 60%, with 41.25% severity remaining at the most effective 400 µg/mL treatment.	[24]
Thymol, Carvacrol	Bee balm *(Monarda spp.*)	*Botrytis cinerea, Fusarium solani*	Monarda essential oils strongly inhibited the tested pathogens; complete inhibition of *Fusarium solani* occurred at higher essential-oil doses, with activity varying by chemotype.	[25]
Myrcene, Humulene	Hop (*Humulus lupulus*)	Model fungal membranes	Hop terpenes altered ergosterol-containing model membranes and caused loss of monolayer material; ergosterol content strongly modulated the effects of humulene and myrcene.	[26]
Pulegone	Pennyroyal (*Mentha pulegium*)	Grape / *Botrytis cinerea*	Pulegone-rich *Mentha pulegium* essential oil reduced *Botrytis cinerea* lesion diameter by approximately 78% on grapevine leaves and 58% on berries.	[20]
Quinolizidine Alkaloids (QAs)	Lupine (*Lupinus polyphyllus*)	*Fusarium oxysporum*	Quinolizidine-rich extract produced >95% mycelial growth inhibition at the highest dose and was fungicidal; activity was associated with the combined quinolizidine-alkaloid composition.	[27]
Quinolizidine alkaloid fraction (sparteine/lupanine-rich)	Lupine (*Lupinus mirabilis*)	*Fusarium oxysporum*	All tested fractions inhibited Fusarium oxysporum (IC_{50} <100 µg/mL); the most active ultrasound-assisted fraction showed the strongest response, while biochemometric analysis implicated minor quinolizidine alkaloids as important contributors.	[28]

Piperine, Capsaicin	Black pepper / habanero pepper	*Aspergillus spp.*	Radial-growth inhibition reached approximately 68% for capsaicin and 86% for piperine; aflatoxin production was inhibited by approximately 73% and 80%, respectively, with increased antioxidant-gene and catalase responses.	[29]
Propyl-propane-thiosulfinate (PTS) / propyl-propane-thiosulfonate (PTSO)	Onion (*Allium cepa*)	*Verticillium dahliae*	Strong vapor-phase and in-soil fungicidal activity; pathogen populations and disease severity were markedly reduced *in planta*.	[30]

Table S3. Detailed study-level evidence on the antibacterial activity of plant secondary metabolites against phytopathogenic bacteria.

Secondary Metabolite	Extract Source	Target Pathogen	Mode of Action and Control Efficiency	Source
Essential-oil constituents (including thymol/carvacrol-rich profiles)	*Trachyspermum ammi* and other plant essential oils	*Xanthomonas citri pv. citri; Pectobacterium carotovorum*; other phytopathogenic bacteria	Trachyspermum ammi essential oil produced the largest inhibition zones (52.25 and 46.25 mm against X. *citri* and P. *carotovorum*, respectively); MIC/MBC and anti-biofilm activity were also demonstrated.	[31]
Tea tree essential-oil constituents	Tea tree (*Melaleuca alternifolia*)	Foodborne bacteria / *Escherichia coli, Salmonella Typhi, Citrobacter koseri*	Tea tree essential oil showed the strongest activity among the tested oils; MIC values were approximately 2.7-6.2 mg/mL across susceptible strains.	[32]
Lemongrass essential oil / citral	Lemongrass (*Cymbopogon citratus*)	Agriculture / *Staphylococcus aureus, Xanthomonas oryzae*	Lemongrass essential oil achieved 100% killing of *Staphylococcus aureus, Xanthomonas oryzae*, and *Escherichia coli* in 45, 75, and 90 min, respectively; citral was also rapidly bactericidal.	[33]
Broad panel of commercial essential oils	Garlic, cinnamon, clove, lemongrass, and other essential-oil sources	Agriculture / 10 distinct pathogens	Several essential oils were active against 7-10 agronomically important pathogens at 500-1000 ppm *in vitro*, supporting broad-spectrum activity of phenol-, aldehyde-, and organosulfur-rich oils.	[34]
Phenolic-rich olive products/by-products	Olive mill wastewater (Blanquette)	Food / *Staphylococcus aureus*, *Escherichia coli*	The most bactericidal olive mill wastewater showed an MBC of 2.5% for target microorganisms; phenolic-rich olive oil reduced the initial inoculum by more than 3 log units within 30 min.	[35]
Piperitenone oxide- and viridiflorol-rich profiles	*Cistus ladaniferus* and *Mentha suaveolens* extracts/essential oils	*Pseudomonas savastanoi; Clavibacter michiganensis*; infected tomato seeds	*In vitro* antibacterial activity was demonstrated; at 4×MIC, methanolic *Cistus ladaniferus* extract resulted in 77.77% germination of Clavibacter inoculated tomato seeds.	[36]

Hydroxytyrosol (Hy)	Olive mill wastewater (AE-2)	Food / *Salmonella Enterica, Staphylococcus aureus*	Hydroxytyrosol-rich olive mill waste extract showed pH-dependent antibacterial activity; predictive models indicated complete inhibition at approximately pH 3.8-3.9 and hydroxytyrosol concentrations around 1200-1314 ppm.	[37]
Peptide Hydrolysates	Agricultural waste proteins	Rice, Tomato / *Xanthomonas oryzae, Pectobacterium carotovorum*	PQLAVF and MDRFL showed mainly membrane-active/cell-leakage and DNA-related effects in Xanthomonas oryzae, whereas VQLMNSL produced intracellular-active effects in *Pectobacterium carotovorum* and *Agrobacterium rhizogenes*.	[38]
Rosmarinic acid and other metabolites	*Salvia/Helichrysum* distillation by-products	*Clavibacter michiganensis* subsp. *nebraskense; Pseudomonas syringae*	Five distillation by-products showed antibacterial activity against C. *michiganensis* (MIC 0.125-1 mg/mL); the study also characterized antioxidant and anti-tyrosinase activities.	[39]
ZnO Nanoparticles	Peanut shell (CNC/ZnO)	Agriculture / General Pathogens	Antibacterial activity should primarily be attributed to ZnO/composite physicochemistry rather than to an intrinsic sulfur-containing PSM effect.	[40]
Tannins, Flavonoids	Pomegranate peel (BC-PGPE)	Biomedical / *Staphylococcus aureus*	The bacterial-cellulose/pomegranate-peel-extract composite reduced *Staphylococcus aureus* growth by 100% and *Escherichia coli* growth by 50%; this was a biomedical composite model rather than a phytopathogen system.	[41]
Cinnamaldehyde, Eugenol	Cinnamon, Clove	Strawberry, Raspberry / *Escherichia coli*	Cinnamon and clove-derived essential-oil fumigation showed antimicrobial activity and supported preservation of red fruits; the study focused on food/pathogen preservation rather than phytopathogenic bacteria.	[42]
Terpenoids (VOCs)	Tree resins (*Boswellia spp.*)	Olive / *Agrobacterium tumefaciens*, *Pseudomonas savastanoi*	Terpene/terpenoid-rich resin essential oils inhibited *Agrobacterium tumefaciens* more strongly than *Pseudomonas savastanoi*; volatile-organic-compound profiles correlated positively with bacterial growth inhibition.	[43]
Thymol-chemotype Origanum vulgare essential oil	*Origanum vulgare*	*Clavibacter michiganensis, Pseudomonas syringae* pv. *phaseolicola*, *Pseudomonas savastanoi, Xanthomonas campestris*	Thymol-rich Origanum vulgare essential oil showed strong *in vitro* antibacterial activity at the highest tested dose; the same study also evaluated selective herbicidal effects on plants.	[44]

Cinnamaldehyde- and carvacrol-rich essential oils	Chinese cinnamon and oregano	Soybean / *Pseudomonas syringae* pv. *glycinea* (Psg), *Curtobacterium flaccumfaciens* pv. *flaccumfaciens* (Cff)	*In planta* leaf treatments showed biological effectiveness up to 86.9% against *Pseudomonas syringae* pv. *glycinea* and approximately 54.6% against *Curtobacterium flaccumfaciens*; cinnamon and oregano oils were the most effective non-phytotoxic treatments.	[45]
Nepetalactone- and carvacrol-rich essential oils	Catnip and oregano	Tomato / *Pseudomonas cichorii, Xanthomonas perforans*	Moderate to high *in vitro* activity was observed against *Pseudomonas cichorii, Pseudomonas syringae,* and *Xanthomonas perforans*; oregano essential oil was generally stronger.	[46]
Catechol, Organic Acids	Maidenhair tree (*Ginkgo biloba*)	Tomato, Pea / *Clavibacter michiganensis*	Ginkgo biloba leaf extracts showed bacteriostatic MICs of 500-1000 µg/mL *in vitro*; full protection was obtained in tomato against *Clavibacter michiganensis* and in pea against *Pseudomonas syringae* pv. *pisi* at higher extract concentrations.	[47]
Linalool- and carvacrol-containing essential oil	Bracken fern (*Pteridium aquilinum*)	Agriculture / *Erwinia amylovora*	Strong *in vitro* activity was observed; the MIC was 0.625 µL/mL against *Erwinia amylovora*, with higher MICs against *Pectobacterium carotovorum* and *Pseudomonas savastanoi*.	[48]
Thymol-rich essential oils	Ajwain (*Carum copticum*) and *Ziziphora clinopodioides*	Apricot / *Pseudomonas syringae*	*In vitro* antibacterial activity against *Pseudomonas syringae* pv. *syringae* was demonstrated by disc, well-diffusion, and vapor phase assays; inhibition increased with essential oil dose.	[49]
Chlorogenic Acid, Flavonoids	Moringa (*Moringa oleifera*)	Apple / *Erwinia amylovora*	All *Moringa oleifera* leaf extracts were bacteriostatic at 1 mg/mL; biofilm formation fell by 80%, swarming motility by 30-65%, and amylovoran synthesis by 63-93%, with significant *in planta* symptom reduction.	[50]
Polyphenol-rich hydroalcoholic extract	Creosote bush (*Larrea tridentata*)	Agriculture / MDR Pathogens	Hydroalcoholic extract and its organic fraction were bactericidal against *Clavibacter michiganensis*, *Pseudomonas syringae*, and *Xanthomonas campestris*; the strongest activity was against X. *campestris* (MIC 0.39 mg/mL; MBC 0.78 mg/mL).	[51]
Peppermint essential-oil constituents	Peppermint (*Mentha piperita*)	Tomato / *Agrobacterium tumefaciens*	Bactericidal MICs ranged from 0.01 to 12.5 mg/mL across *Agrobacterium strains*; 200 mg/mL peppermint essential oil completely prevented crown-gall tumor formation on tomato.	[52]

Table S4. Detailed characteristics and biological performance of nanoformulations incorporating plant secondary metabolites for plant disease management.

Crop / host	Target pathogen(s)	PSM(s)	Nanoformulation type	Detailed formulation parameters	Main quantitative results	Reported advantages	Limitations noted	Summary	Source
Soybean (*Glycine max*)	*Xanthomonas axonopodis* pv. *glycine*	Thymol (EO component)	O/W nanoemulsion	Surfactant: Quillaja saponin; thymol:saponin 6:1 (w/v); probe sonication 20 kHz, 60% amplitude, 5 s on/off pulses; 50 min sonication produced "most stable" NE; z-average 274 ± 2 nm, PDI 0.1, zeta -31 mV (stability confirmed; after 3 months: mean droplet size 293 ± 2.7 nm, PDI 0.15, zeta -32 mV). Application concentrations: 0.01-0.06% (v/v).	*In vitro*: NE (0.02-0.06% v/v) reported complete inhibition (0.0 log CFU/mL) vs control; pot: seed + foliar (0.03-0.06% v/v) lowered DS to 33.3-3.3% and increased PEDC 54.9-95.4%.	Natural surfactant; improved dispersion of hydrophobic thymol; stability evaluation; dual "disease control + growth promotion" rationale.	Primarily controlled pot experiments; field-scale validation not provided in accessible text; DLS z-average larger than many droplets seen by TEM (reported interpretation.	The saponin-stabilized thymol NE showed strong antibacterial activities against soybean bacterial pustule pathogens and improved disease parameters in pots. The paper reports the physicochemical characterization and stability of the paper and proposes thymol NE as an alternative to conventional bactericides under controlled conditions, compatible with plants.	[53]

Citrus fruit (postharvest context)	*Penicillium italicum*	Garlic oil (GO; essential oil mixture)	Nanoemulsion	Surfactant blend: Tween 80 + Span 80; optimal HLB for GO 14; Box-Behnken optimization: GO 5.5%, Smix 10%, ultrasonication 5 min, power 50%; particle size 52.27 nm; PDI, zeta: unspecified in accessible abstract.	MIC improved from 3.7% (GO) to 0.01265% (GO-NE), ~300× improvement; *in vivo* citrus assay reported successful inhibition of *P. italicum*.	Strong dose-sparing effect; mechanistic multi-modal evidence; addresses hydrophobicity/instability of bulk oil.	Full formulation fine-parameters (PDI/zeta) and detailed *in vivo* protocol endpoints are unspecified in the abstract-only view.	Garlic oil NE produced ultrasonically significantly reduced the MIC against P. italicum and was effective in a citrus postharvest context. The work is on process optimization, and general mechanistic characterization underlying membrane/biomolecule disruption.	[54]
Rice leaf assay; field comparison mentioned	*Magnaporthe oryzae*	Green tea essential oil (major actives not detailed here)	Nanoemulsion	Surfactants used: Tween 80 and Span 80; size/PDI/zeta: unspecified in accessible abstract; dose: rice leaf assay at 2% NE; field comparison against tricyclazole noted.	Rice leaf assay: 2% NE decreased lesion area; "field conditions" performance described as better than one comparator and comparable to tricyclazole.	Demonstrates controlled plant-tissue protection and suggests field relevance; mechanistic framing (membrane damage/ATP reduction).	Quantitative field yield/disease metrics and full formulation parameters are unspecified in the abstract view.	This study reports green tea essential oil NE antifungal activity against rice blast fungus as supported by the mechanistic assays and lesion reduction on rice leaves. It also suggests real-world promise in comparison to a standard fungicide, although full details of field protocol were not available in the abstract snapshot.	[55]

Cucumber (*Cucumis sativus*)	*Botrytis cinerea* (MF996363); also postharvest rot fungi mentioned	Essential oils: clove, black seed, lemon, orange (GC/MS in broader work)	Ultrasound nanoemulsions	Reported NE droplet sizes: 82.6 nm (clove), 95.9 nm (black seed), 131.9 nm (lemon), 117.4 nm (orange); formulated at 10% concentration using Tween 80 under ultrasonic emulsification; PDI/zeta: unspecified in accessible text (referenced to earlier work).	At 5000 ppm, black seed and clove NEs reduced mycelial growth; a clove:black seed (2:1 v/v) NE mix completely inhibited 100% conidia sporulation of fungal isolates; soaking cucumbers with NE mix 2.0% reported 100% suppression of postharvest fruit rot after artificial infestation.	Multi-pathogen postharvest relevance; emphasizes eco-friendly alternative with toxicity checks described (rats, *Vibrio fischeri*).	Some formulation characterization (PDI/zeta, long-term stability) not provided in the accessible excerpt; integration into real postharvest supply chain conditions remains to be validated.	This work indicates ultrasonically generated essential oil nanoemulsions which strongly suppressed B. *cinerea* and other postharvest rot fungi on cucumber including complete control in a fruit soaking protocol for a clove:black seed NE mixture. It supports nanoemulsions as practical postharvest interventions but cites some characterization to prior work.	[56]

Tomato fruit (postharvest gray mold context)	*Botrytis cinerea*	Thymol	Nanoemulsion (nano-thymol emulsion)	Droplet size: 33.45 ± 0.04 nm; zeta -19.1 ± 0.33 mV; PDI 0.339 (reported); surfactant system: unspecified in accessible excerpt; tested concentration range included 0.02-0.10% (w/v) and 0.05% for several assays.	Reported complete inhibition of mycelial growth at 0.05% (w/v); tomato gray mold "lesion length reduction" reported up to 78.5% at 0.05% (w/v) under described conditions.	Very small droplet size; emphasizes improved dispersibility and antifungal performance vs bulk thymol.	Some formulation composition details not visible in the excerpt; full postharvest handling practicality (residues, sensory impact) not specified here.	A nano-thymol emulsion with ~33 nm droplet size was found to effectively inhibit B. *cinerea* and reduce gray mold lesions on tomato fruit at a low loading of thymol. It represents a nanoemulsification that can enhance a volatile phenolic monoterpene for controlling postharvest diseases.	[57]
Maize seeds (*Zea mays*)	*Penicillium verrucosum*	*Nigella sativa* oil (black seed oil)	Nanoemulsions	Emulsifiers: Tween 20 or Tween 80; sonication times 0/5/10 min produced droplet sizes (nm): Tween20-248.1, 185.7, 168.6; Tween80-345.3, 211.6, 188.6. PDI range 0.181-0.353; zeta potentials: up to -46.74 mV (Tween20, 10 min) and -48.82 mV (Tween80, 10 min).	After 8 days: complete inhibition at 100% concentration using 10_T80 and 10_T20 formulations; other NEs showed ~93-98% inhibition at certain conditions (Table values reported).	Demonstrates how surfactant choice + sonication time modulate size/charge and antifungal efficacy; provides stability indicators (zeta, separation %).	Concentration framing ("25/50/100%") can be challenging for field translation; broader agro-ecosystem safety and scale-up remain future work.	This Frontiers study establishes a systematic link between sonication time, Tween type, nanoemulsion droplet physicochemistry and significant inhibition of P. *verrucosum* relevant to maize seed infection. The data favor formulation optimization as a lever to maximize the plant-protective bioactivity of seed-based plant-based oils.	[58]

(Host plant not clearly specified in excerpt)	*Colletotrichum* sp.; *Fusarium oxysporum*	Cinnamaldehyde (from cinnamon oil) + annonaceous acetogenins (from *Annona* seed extract)	Botanical nanoemulsion	Physicochemistry: droplet size 245.3 nm, PDI 0.499, zeta -44.2 mV; concentration tested included 0.35% NE; surfactant system: unspecified in accessible excerpt.	*In vitro*: 0.35% NE inhibited *Colletotrichum* sp. 86.7% and *F. oxysporum* 93.2% (reported).	Combines two bioactives in a single NE; strong negative zeta suggests colloidal stability; includes both *in vitro* and greenhouse-level tests.	Host plant species and detailed greenhouse protocols (timing, replication, disease scoring) are unspecified in the excerpt; scale-up and field validation not shown here.	An accessible botanical NE combining the active principles of cinnamon and annonaceous acetogenins was tested for *in vitro* inhibition of Colletotrichum and Fusarium and in greenhouse bioassays. The droplet size, PDI and zeta reported are indicative of a nanoemulsion system of moderate polydispersity but high charge.	[59]
Banana fruit (*Musa AAA*)	*Colletotrichum musae* (QB6)	Lemongrass essential oil (LEO; major actives via GC-MS not shown in excerpt)	Optimized nanoemulsion	Optimization via Box-Behnken/RSM: Tween-80 17.22 g/L, homogenization speed 10,180 rpm, ultrasonication 20 min; droplet diameter 153.2 nm, PDI 0.22, zeta -12.0 mV; stability tracked (DD <200 nm; PDI <0.3 over 120 days described).	*In vitro*: PIRG 100% (complete inhibition) for optimized LEO-NE vs 85.94% for non-nano LEO; biomass inhibition 100% vs 88.39% (pure LEO). *In vivo*: treated bananas showed no outward damage after 6 days (qualitative endpoint).	Clear optimization-to-performance chain; reports droplet size/PDI/zeta; emphasizes stability and equivalence to synthetic fungicide comparator.	Controlled lab conditions may not reflect commercial postharvest environments; biological validation scope and stability assessments described as restricted.	The work modified parameters for lemongrass EO nanoemulsion and showed total inhibition of C. *musae in vitro* with qualitative suppression of lesion in banana fruit assays. It explicitly reports key colloidal metrics (DD/PDI/zeta) and highlights the need for real-world after-harvest validation.	[60]

Tomato fruit (*Solanum lycopersicum*)	*Colletotrichum gloeosporioides*	Thyme essential oil (major thymol 58.77%)	Thyme EO nanoemulsion incorporated into chitosan coating	NE composition: phosphate buffer pH 6.68, modified lecithin 0.1% w/w, Tween80 2% v/v; dispersed phase included soybean oil 1% v/v + thyme oil 1% v/v; process: Ultra-Turrax 10,000 rpm 10 min, ultrasonication 10 min at 20°C, then high-pressure homogenization 100 MPa, 2 cycles; mean droplet size reported 60.21 ± 1.8 nm (PDI value not clearly separated in accessible text); coating: chitosan 0.8% (w/v) + Nano-(T)-EO 1% (v/v); zeta potential: unspecified (authors call for more zeta-based characterization).	*In vitro*: chitosan 0.8% inhibited radial mycelial growth 78%; Nano-(T)-EO (1% v/v) achieved complete inhibition *in vitro* (reported); Nano-(T)-EO-CS inhibited spore germination 100%; MIC for spore germination for Nano-(T)-EO reported 1.92 µL/mL; *in vivo*: disease incidence reduced 50% vs control (Sporekill® 40%) after cold storage protocol (reported).	Integrates NE into edible chitosan coating; shows postharvest disease incidence reduction and quality/defense-associated endpoints (e.g., chitinase timing).	Authors note dependence on processing conditions; release rate of actives and more colloidal characterization (including zeta) suggested for future.	A thyme-oil nanoemulsion incorporated into a chitosan coating reduced metrics of tomato anthracnose levels during freezing and shelf simulation, with strong *in vitro* suppression of C. *gloeosporioides* spore germination. The study describes a rather detailed synthesis chain (lecithin/Tween80 + high pressure homogenization) but points out remaining gaps in release and zeta-driven stability characterization.	[61]

Cucumber (*Cucumis sativus*)	*Podosphaera fusca* (powdery mildew; synonyms noted in text)	Essential oils: savory (*Satureja khuzistanica*), lemongrass (*Cymbopogon citratus*), clove (*Syzygium aromaticum*), etc. (major constituents via GC-MS)	Plant essential oil nanoemulsions (PEO-NEs)	Surfactant: Tween 80; DLS mean diameters day1/90 and distribution values reported (examples): savory 161.68 ± 16.24 nm (day1) → 310.87 ± 37.51 nm (day90); clove 187.12 ± 18.77 nm → 199.29 ± 19.54 nm; lemongrass 157.22 ± 16.11 nm → 230.38 ± 27.55 nm; PDI-like distribution values ~0.111-0.196 reported; zeta: unspecified in accessible excerpt. Application: 1-3 g/L.	*In vitro*: savory/lemon grass/clove NEs at 1-3 g/L inhibited conidia germination by 83%, 74%, 83% (as reported); greenhouse: at 3 g/L reduced disease severity by 54.05%, 40.54%, 35.13%, outperforming Topas and Stroby in that study; preventive 48 h before inoculation reduced severity by 72.41%, 55.17%, 41.3%.	Comparative performance vs synthetic fungicides; includes chemistry profiling and host phenolic response context; tracks storage-related size changes.	Authors indicate additional studies are needed for mechanism optimization and “field conditions.”	In the present study, several plant-EO nanoemulsions were screened and it was found that NEs of savory, lemongrass and clove were effective against cucumber powdery mildew in greenhouse trials and preventive application could improve the results. Reported DLS monitoring suggests differential stability among oils over 90 days, supporting formulation-dependent shelf-life considerations.	[62]

Wheat seeds (phytotoxicity); pathogens from culture	*Aspergillus niger* (ATCC16888); *Aspergillus brasiliensis* (ATCC16404); *Fusarium oxysporum*; *Penicillium citrinum*	*Sideritis serrata* extract (ethanol extract; multiple secondary metabolite classes implied)	Chitosan nanoparticles loaded with plant extract (NPCH-SID)	Ionic gelation: chitosan 0.2% in 1% acetic acid (overnight); combined with 1% Tween 80; added 0.2% TPP dropwise; full size/PDI/zeta values appear in Table 1 but are unspecified here due to non-extracted table contents; encapsulation efficiency optimized (reported).	Abstract reports up to 156,140-fold improvement in activity against *A. brasiliensis* vs free extract; phytotoxicity: ~95% germination and improved root/shoot growth.	Open-access; integrates efficacy with phytotoxicity/biostimulatory assessment; highlights stabilization/delivery advantage for plant-derived compounds.	Authors explicitly frame greenhouse/field testing as future work; particle metrics require table extraction for full reporting.	Chitosan nanoparticles loaded with a Sideritis extract showed very large reported fold-improvements in antifungal activity against a few fungi and low phytotoxicity on wheat seeds. The study suggests NPCH-SID could be a helpful tool in sustainable agriculture but adds that more greenhouse/field validation is necessary.	[63]

Table S5. Detailed overview of encapsulation technologies for plant secondary metabolites used in plant disease management.

Crop / host	Target pathogen(s)	PSM(s)	Encapsulation / carrier type	Detailed formulation parameters	Main quantitative results	Reported advantages	Limitations noted	Summary	Source
Unspecified (phytopathogen isolate; cereal pathogen context)	*Fusarium graminearum*	*Cymbopogon martinii* essential oil (EO) (major constituents: unspecified)	Chitosan nanoparticles encapsulating EO (Ce-CMEO NPs)	Size ~455-480 nm (varied by ppm); ζ ~ +37-39 mV; release over 48 h: ~97.94% at pH 7.4 vs ~43.37% at pH 1.5; antifungal comparisons used Ce-CMEO NP concentrations up to 700 ppm (free EO up to 900 ppm). PDI/EE/LC: unspecified.	700 ppm Ce-CMEO NPs: reported complete reduction of fungal growth and mycotoxins, compared with 900 ppm needed for free EO.	Dose-sparing vs free EO; pH-dependent release; improved bioactivity retention implied by NP delivery.	*In vitro* only; field/greenhouse validation and non-target safety not addressed in the extracted text.	Demonstrates that chitosan encapsulation can substantially lower the EO dose required for suppressing a major phytopathogenic fungus and associated mycotoxins under *in vitro* conditions. Release behavior suggests environment-dependent delivery that could be leveraged in applied formulations.	[64]

Citrus fruit context (sour rot); host species not specified in excerpt	*Geotrichum citri-aurantii*	Thymol	β-cyclodextrin inclusion complex (thymol-β-CD)	*In vitro* inhibition reported at 4 g/L inclusion complex comparable to 0.5 g/L thymol; additional formulation parameters (particle size, ζ, EE/LC, release profile): unspecified in the extracted abstract text.	Inclusion complex inhibited growth and was associated with decreased membrane integrity; quantitative mechanistic endpoints not specified in excerpt.	Improved aqueous handling/compatibility implied by cyclodextrin delivery; dose comparability provided vs free thymol.	Many requested formulation details not present in the abstract excerpt; confirm full methods/results via DOI.	Provides experimental evidence that cyclodextrin complexation can deliver thymol activity against citrus sour rot pathogen G. *citri-aurantii*. The abstract-level data indicate fungistatic/fungicidal activity with membrane-associated effects.	[65]
Unspecified	*Colletotrichum nymphaeae*	Thyme EO + dill EO	Nano-encapsulation with copper nanoparticles (Cu NPs)	Detailed parameters (surfactant/cross linker, size/PDI/ζ, EE/LC, release profile, applied concentration): unspecified from accessible metadata/snippets.	Main quantitative efficacy metrics: unspecified in accessible snippet.	Claimed enhancement of antifungal activity via nano-encapsulation (paper title/metadata).	Many experimental details not accessible in the retrieved snippet; verify via DOI/full text.	This study is frequently cited as an EO-CuNP nano-encapsulation approach targeting C. *nymphaeae*. It is included for completeness of the 2018-2026 experimental record, but key formulation/efficacy details should be extracted directly from the full paper.	[66]

Lemon fruit (*Citrus limon* implied)	*Geotrichum citri-aurantii*	Thymol	HP-β-cyclodextrin microencapsulation (HP-β-CD-thymol)	Encapsulation via microwave irradiation + spray drying; EE ~82.8 ± 0.6%; applied 25 mM and 50 mM; inoculum 10^5 CFU/mL (arthrospores); comparator fungicide: propiconazole 1% v/v. Particle size/PDI/ζ and quantitative release curve: unspecified in extracted lines.	HP-β-CD-thymol reduced disease incidence/severity; 50 mM reported comparable performance to propiconazole at key timepoints; treatment also associated with enhanced phenolic/antioxidant enzyme responses.	Increases practical usability of thymol (solubility/handling) and supports controlled delivery in postharvest disease management; links antimicrobial delivery to host biochemical responses.	Postharvest model (not field); some formulation metrics (size/ζ/release kinetics) not fully specified in extracted lines; scaling/regulatory not resolved.	Demonstrates a cyclodextrin encapsulation route that enables thymol deployment as a postharvest treatment against sour rot. The study is notable for combining disease metrics with host biochemical defense markers, supporting a dual "direct antifungal + induced resistance" framing.	[67]

Tomato (*Solanum lycopersicum*)	*Xanthomonas euvesicatoria* (bacterial spot)	*Satureja montana* EO (major constituents reported: carvacrol 43%, p-cymene 17%, γ-terpinene 13%, thymol 4%)	EO encapsulated in zein nanoparticles (protein nanocarrier)	Anti-solvent precipitation; NP formation efficiency ~80%; EO encapsulation efficiency ~20.58% (≈ 1 mg EO/mL nanoformulation; zein final conc. 4 mg/mL); size range during storage ~122-160 nm; ζ ~ +30 mV; plant treatment: 0.4 mg/mL EO, with EO-ZNPs containing 0.4 mg/mL EO in 1.6 mg/mL zein; infection protocol included spray inoculation (OD600 0.25), then treatment spray. PDI numeric values: unspecified in extracted lines.	Treatments reduced X. euvesicatoria load (reported range ~1.6-fold to ~202-fold reductions depending on treatment), alongside improved plant health metrics and altered defense-related pathways.	Bio-based carrier; improved EO stability/field-handling potential; integrates antimicrobial action with plant response.	Authors explicitly note the need for further field studies/long-term clarification.	Provides a strong example of a PSM delivery system evaluated directly in a plant-bacterial pathosystem, with quantified pathogen reduction and host-response readouts. The work is particularly useful for reviews discussing "nanoformulation as both antimicrobial and elicitor/primer.	[68]

Tomato (*Solanum lycopersicum*)	*Fusarium solani* (root rot)	*Thymus vulgaris* EO	Chitosan nanoparticles loaded with EO (ionic gelation)	Ionic gelation with TPP crosslinker: chitosan 0.2 g in 1% acetic acid, pH adjusted (~4.6); EO addition (~200 µL); TPP 8 mL (2 mg/mL) added dropwise (1 mL/min), stirred; TEM size ~20-80 nm; tested concentrations 0.2-1.0% *in vitro; in vivo* outcomes reported at 1%. PDI/ζ/EE/LC: unspecified in extracted lines.	*In vitro*: ~89.6% growth reduction at 1% ThE-CsNPs after 7 days; *in vivo*: disease severity ~92.4% in positive control vs ~4.3% (infected + treated) and ~2.3% (primed) reported.	Strong suppression of disease severity; nanoformulation associated with defense-related physiological changes; bio-based formulation.	No field trial reported; some formulation parameters (ζ/PDI/EE) not specified in extracted lines; broader non-target safety/regulatory not addressed.	Demonstrates a classic ionic-gelation chitosan NP route for EO delivery with strong *in vitro* and seedling-level disease suppression. The paper is useful in reviews that connect EO nano-delivery with induced resistance and gene-expression framing.	[69]

Citrus fruit (species unspecified)	*Penicillium digitatum* (green mold)	Cinnamaldehyde	γ-cyclodextrin inclusion complex (γ-CDCL)	Preparation: γ-CD 10 g/25 mL water at 45°C; cinnamaldehyde 0.4 mL in ethanol (1:4), dropwise; EE ~75.63 ± 1.04%; *in vivo* dip: 4× and 8× MFC for 60 s; prochloraz 0.025% v/v control; particle size/PDI/ζ: unspecified (solid inclusion complex).	Natural storage: after 30 d, disease incidence reported ~6.67% (8× MFC γ-CDCL) vs ~22.67% (control) and ~10.67% (prochloraz).	Improved solubility/stability of cinnamaldehyde; competitive performance to a commercial fungicide in a postharvest context; includes mechanistic endpoints.	Postharvest focus (not field); scale-up/cost and regulatory readiness not resolved.	A well-documented cyclodextrin delivery paper linking formulation (EE) to both mechanistic antifungal assays and fruit-level disease outcomes under inoculated and natural storage conditions-high utility for review tables and mechanism sections.	[70]

Black pine (*Pinus nigra*)	*Phytophthora cactorum*	*Thymus serpyllum* EO (carvacrol-rich; detailed composition in text extract)	Lignin nanoparticles loaded with EO (EO-LNPs)	Release assessed by dialysis + HPLC; cumulative release (EO-LNPs vs pure EO) over time reported (e.g., EO-LNPs ~79.7% at 24 h; ~96.0% at 48 h; ~98.7% at 72 h); particle diameter noted around ~100 nm in extracted text; drug loading efficiency >60% (reported). Applied concentration for certain assays: 135 µg/mL; full DLS/PDI/ζ not clearly specified in extracted text.	*In vitro* EC50 values reported (EO vs EO-LNPs); *in vivo* inhibition rate reported ~93% (EO) vs ~100% (EO-LNPs) in reducing seedling mortality; 100% inhibition noted at 240 µg/mL *in vitro* timepoint context.	Bio-based nanocarrier; sustained/retarded release relative to pure EO; greenhouse efficacy suggests practical promise for nursery disease contexts.	Field validation not reported; some standard nano-metrics (ζ/PDI) are not fully recoverable from the extracted text and should be confirmed from the publisher version.	A flagship example of lignin nanocarriers used beyond postharvest fruit-targeting an oomycete disease with both *in vitro* inhibitory metrics and greenhouse seedling outcomes, supported by explicit release profiling.	[71]

Grapevine pathogen set (host plant not directly assayed in extracted text)	*Botrytis cinerea*; *Phaeoacremonium minimum*; *Phaeomoniella chlamydospora*	Neem oil (*Azadirachta indica* seed oil)	Tannins-lignin mixed nanocapsules	Mean diameter ~150-300 nm, low polydispersity; negative surface charge (ζ not numerically specified in extracted lines); reported efficacy enhancement against P. minimum by >1-2 orders of magnitude; other requested parameters (EE/LC, release curve, applied concentration): unspecified in extracted text.	Enhanced activity specific to P. *minimum*; no additional activity vs P. *chlamydospora* and B. *cinerea* in extracted summary.	Demonstrates dose-sparing potential of encapsulated neem oil; highlights selective spectrum improvements via carrier design.	Full experimental details restricted in the accessible view; no plant-level trials in the extracted portion; specificity suggests formulation may need tailoring by pathogen.	Useful for reviews addressing lignin/tannin "green nanocapsules" and the reality that encapsulation may improve efficacy in a pathogen-dependent manner rather than universally.	[72]

Wood-rot model (bioprotection context)	*Trametes versicolor*; *Pleurotus ostreatus*; *Gloeophyllum trabeum*; *Poria monticola*	Thymus spp. EOs (carvacrol-/thymol-/p-cymene-rich profiles by species)	Lignin nanoparticles with entrapped EOs (EOLs)	Preparation via acetone dissolution + dialysis (OSL 300 mg + EO 100 mg); DLE/DLC reported (e.g., DLE 50-75%, DLC 32-48% depending on EO type); SEM showed broad polydispersity (100-200 nm to micrometers); EO release tracked for 7 days in fungal medium with differentiated kinetics by EO chemotype; MICs reported 0.05-0.60 mg/mL. ζ/PDI (DLS): unspecified (SEM-based size).	Encapsulation produced lower MICs for brown-rot fungi vs free EO in certain comparisons (e.g., G. trabeum, P. monticola), while effects vs white-rot fungi were less improved.	Clear loading and release data; highlights how carrier degradability (lignin-consuming fungi) affects apparent efficacy-valuable mechanistic nuance for reviews.	Application is wood/bioprotection rather than a crop field pathosystem; suggests optimization needed for white-rot fungi that can metabolize lignin carriers.	Demonstrates that lignin nanocarriers can provide multi-day EO release and dose-reduction in certain fungal classes, while also revealing a key limitation: pathogens capable of degrading lignin may reduce delivery advantage.	[73]

Banana fruit (*Musa* spp.)	*Colletotrichum musae* (banana anthracnose)	Thymol	Zein nanoparticles (protein nanoencapsulation)	High-shear emulsification; EE ~70%; DLS effective diameter ~300 nm; PDI ~0.337; ζ ~ −32 mV; 40-day storage stability: ~99% retention at 4°C and ~97% at room temperature. Applied concentration for *in vivo* spray: unspecified in extracted lines.	Reported 200-300× improvement in *in vitro* antifungal activity vs thymol; delayed symptom formation by NP film deposition on banana epidermis.	Strong stability/retention; clear physicochemical reporting; mechanistic hypothesis for surface film deposition.	Applied dose details and broader field logistics not fully recoverable from extracted lines; scale-up and regulatory steps remain.	A high-quality example of zein nanocarriers enabling stability and large apparent potency gains for a volatile phenolic PSM against a key postharvest pathogen. Particularly useful for review sections on protein nanocarriers and "retention-driven" efficacy.	[74]

Cucumber (*Cucumis sativus*)	*Podosphaera fusca*	Celery seed EO (*Apium graveolens*; major constituents unspecified in extracted lines)	Chitosan nanoparticles (ionic gelation; EO encapsulation)	Ionic gelation + Tween 80; sodium tripolyphosphate 0.04 g/10 mL; size ~113.25 nm; ζ ~ −31.35 mV; EE/LC depend on chitosan:EO ratio (max EE ~30.31% at 1:0.5; max LC ~25.36% at 1:1); release in acetate buffer pH 3; Korsmeyer-Peppas fit R^2 ~0.96945, n ~0.173857 (Fickian); greenhouse doses 50-400 µg/mL, with 400 µg/mL phytotoxic, 200 µg/mL → ~75% disease severity reduction.	Best reported greenhouse outcome in extracted lines: ~75% reduction in powdery mildew severity at 200 µg/mL; toxicity observed at higher dose (400 µg/mL).	Rich reporting of formulation (EE/LC, release model parameters) and plant defense endpoints; clear dose-response and phytotoxicity boundary.	Greenhouse only; phytotoxicity at high dose; field/scale-up/ecotox not addressed.	A highly "review-ready" encapsulation study: reports formulation chemistry, release kinetics, and greenhouse disease efficacy with phytotoxicity limits. Also explicitly links nano-delivery to host defense enzyme and gene-expression activation.	[75]

Table S6. Detailed overview of controlled-release systems containing plant secondary metabolites for plant disease management.

Crop/host	Target pathogen(s)	PSM(s)	Encapsulation / delivery system	Controlled-release mechanism type	Detailed formulation parameters	Main quantitative results	Reported advantages	Limitations noted	Summary	Source
Tomato (*Solanum lycopersicum* var. Oxheart)	*Xanthomonas euvesicatoria*	*Satureja montana* EO (carvacrol 43%, p-cymene 17%, γ-terpinene 13%, thymol 4%)	Zein nanoparticles (EO-Zein NPs)	Controlled delivery implied via stable protein matrix (explicit release model: unspecified)	Anti-solvent precipitation; zein in 70% ethanol; EO added; dispersed into water; non-encapsulated EO removed (PD-10 column). EE 20.58 ± 1.82% (≈1 mg EO/mL nanoformulation). Sizes 122-160 nm; ζ ~+30 mV; stability tracked 90 d at 4 °C. Applied: EO 0.4 mg/mL; EO-Zein NPs 0.4 mg/mL EO in 1.6 mg/mL zein; zein NPs 1.6 mg/mL	In infected plants, treatments reduced Xeu load: 38-fold (EO-Zein NPs) and up to 202-fold (zein NPs) vs infected control; free EO ~1.6-fold reduction	Strong *in planta* bacterial suppression; stable colloids; suggests carrier itself can contribute to efficacy; supports prophylactic use	No release kinetics; strong effect of empty zein NPs complicates attribution to PSM; field validation needed	Illustrates "controlled delivery as persistence + stability": zein NPs provided stable carriers (months at 4 °C) and, with or without EO, substantially reduced Xeu loads in tomato, supporting nanocarrier-based dose efficiency even when release curves are not reported.	[68]

Chinese cabbage (soft-rot context)	*Pectobacterium carotovorum* subsp. *carotovorum*	*Athyrium sinense* EO (n-hexadecanoic acid 32.18%; linoleic acid 18.28%; phytol 8.67%)	Chitosan nanoparticles via ionic gelation (AEO-CSNPs)	Fickian diffusion release (explicit "Fickian release" behavior reported)	Ion-gel method. Optimal CS:AEO ratio 1:0.25; ζ +25.7 mV; EE 48.43%; LC 6.23%. Size/PDI: unspecified	MIC 0.16 mg/mL; MBC 0.63 mg/mL. *In vivo* (72 h, 28 °C): preventive effect 82.5%, therapeutic effect 80.43%	Explicit diffusion-governed controlled-release framing; persistence via time-kill curves; improved *in vivo* performance vs free EO	Limited kinetic detail (no curve/fit parameters); short *in vivo* window; field validation, formulation robustness, and phytotoxicity thresholds not established	Demonstrates diffusion-controlled release for a bacterial phytopathosystem: the chitosan NP system improved antibacterial potency and short-term *in vivo* control of soft rot while maintaining activity over multi-day lab timescales.	[76]
Shatangju mandarin slices (fresh-cut)	*Penicillium digitatum*	Carvacrol	Carvacrol/β-cyclodextrin inclusion complex (fumigation)	Equilibrium partitioning with volatilization-buffering (fumigation); kinetic model: unspecified	Co-precipitation inclusion complex; characterized by SEM/FTIR/XRD/TGA/DTA. Particle size/PDI/ζ, EE/LC: unspecified	*In vivo* slices: incidence 75.0% and severity 46.7% (inclusion complex) vs incidence 100% and severity 69.2% (pure carvacrol)	Non-contact delivery (fumigation); improved efficacy and sensory acceptance vs free carvacrol	High residual incidence indicates partial control; no explicit release kinetics; limited to fresh-cut context	Shows inclusion-complex delivery as a practical tool for controlled availability: Carvacrol complexation with β-CD improved postharvest antifungal activity relative to free carvacrol and aided quality maintenance under a non-contact fumigation scheme.	[77]

Cucumber (*Cucumis sativus*) seedlings	Podosphaera fusca	Spinach seed EO (predominant: aromadendrene, L-limonene, trans-anethole)	Chitosan nanoparticles loaded with EO (SSEO-LCNPs)	Korsmeyer-Peppas model used to describe release (diffusion/relaxation; parameters: unspecified in abstract)	DLS size 116.2 nm; ζ -27.7 mV; spherical morphology. Highest EE/LC at chitosan:SSEO 1:1 (EE/LC values: unspecified in abstract). Applied 400 µg/mL (foliar)	Foliar 400 µg/mL significantly reduced powdery mildew severity (quantitative %: unspecified in abstract); induced phenolics/flavonoids and defense enzymes (quantitative values: unspecified in abstract)	Clear "controlled release + defense induction" framing; characterized colloid size and ζ; integrates pathogen suppression with host biochemical responses	Release parameters not fully visible from abstract; greenhouse only; need field durability (rainfastness/UV) and dose-phytotoxicity windowing	A novel chitosan-NP controlled-release biopesticide pattern: nanoencapsulation allowed an EO with known volatility/instability issues to be delivered *in planta* with reported disease suppression and host defense activation, with Korsmeyer-Peppas used for release description.	[78]
Citrus fruit	*Penicillium italicum* (prochloraz-resistant strain)	(E)-2-octenal	(E)-2-octenal/β-cyclodextrin inclusion complex (O/β-CDIC)	Inclusion complex reservoir (equilibrium partitioning); improved phase solubility/thermal stability; kinetic model: unspecified	Saturated aqueous solution method + freeze-drying. Particle size/PDI/ζ: unspecified	MIC 2.00 mg/mL; MFC 8.00 mg/mL against P. *italicum*; reduced blue-mold incidence and maintained fruit quality (quantitative incidence: unspecified in accessible abstract)	Targets fungicide-resistant pathogen; stabilizes volatile aldehyde; combines physicochemical characterization with *in vivo* fruit outcomes	Limited accessible detail on release kinetics and *in vivo* effect size; field validation and sensory impacts not resolved	Notes Points out cyclodextrin complexes as controlled-availability tools for volatile PSMs under fungicide-resistance pressure. Reports EE/LC and clear MIC/MFC endpoints. Demonstrates practical fruit protection *in vivo*.	[79]

Table S7. Detailed overview of green-synthesized nanoparticles obtained using plant-derived reducing and stabilizing agents for plant disease management.

Crop/host	Target pathogen(s)	PSM(s)	Green synthesis approach	Nanoparticle type	Characterization	Antimicrobial/ antifungal efficacy metrics	Reported advantages	Limitations noted	Summary	Source
Tomato (*Solanum lycopersicum*)	*Ralstonia solanacearum*	Tomato leaf extract phytochemicals (unspecified) + chitosan (biopolymer)	Plant-extract-mediated reduction/capping plus biopolymer entrapment (chitosan)	AgNPs entrapped in chitosan nanoparticles (CH-AgNPs)	AgNPs <87 nm; CH-AgNPs <271 nm; stability maintained up to 30 days (no major physicochemical alteration). ζ unspecified. EE/LC: not applicable (no cargo; Ag is active).	Antibacterial activity at micromolar concentrations after 48 h; CH-AgNPs at 256 µmol L^{-1} reduced bacterial wilt incidence in a partially resistant tomato genotype (not in a susceptible line).	Combines plant-based AgNP synthesis with a stable chitosan matrix; reported stability up to 30 days; disease suppression *in planta* for a resistant genotype.	Host-genotype dependence (worked in partially resistant line only); full field validation and broader host testing unspecified.	Demonstrates a "self-species" strategy (tomato extract → AgNPs) and chitosan entrapment to create relatively stable antibacterial nanomaterials that can reduce bacterial wilt incidence in a resistant background, though efficacy may be contingent on host susceptibility.	[80]

Faba bean (*Vicia faba* L. *major*)	*Botrytis cinerea*	Olive leaf extract constituents implicated: phenols, flavonoids, glycosides, proteins (specific compounds unspecified)	Plant-extract-mediated reduction/capping	ZnO nanoparticles	SEM: spherical-to-hexagonal; size 11-60 nm; XRD dominant size ~17.41 nm. DLS/PDI/ζ unspecified. EE/LC: not applicable.	*In vitro* radial growth test: strong inhibition at higher ZnO NP concentrations (400-1000 ppm) after 12 days; *in vivo* foliar spray (detached leaves and whole plants): disease severity and infection-related parameters reduced vs infected control (exact % unspecified in abstract).	Plant-extract route; small ZnO size; includes *in vivo* foliar spray evaluation (beyond plate assays).	Many formulation details (precursor concentration, ζ/PDI) and full quantitative *in planta* outcomes not fully specified in accessible abstract; large-scale/field validation unspecified.	Provides a plant-extract green synthesis route for ZnO NPs and shows both *in vitro* inhibition and *in vivo* protection in faba bean against grey mould, but key formulation parameters and detailed disease metrics should be more completely reported for reproducibility.	[81]

Rice (pathogen context: bacterial leaf blight)	*Xanthomonas oryzae* pv. *oryzae* (Xoo)	Plant extracts from *Arctium lappa* (fruit), *Solanum melongena* (leaves), *Taraxacum mongolicum* (leaves); specific PSMs unspecified	Plant-extract-mediated reduction/capping	AgNPs	TEM/SEM: spherical; average diameters ~20.18, 21.00, 40.08 nm (by extract type). DLS/PDI/ζ unspecified. EE/LC: not applicable.	Strongest bacteriostasis at 20 µg/mL vs Xoo (inhibition zones up to 16.5 mm depending on AgNP type); OD600 reduction in broth up to 72.10% at 20 µg/mL; inhibited biofilm formation and swarming motility.	Simple aqueous extraction + mild synthesis conditions; multiple botanical sources compared; includes anti-biofilm and motility endpoints.	*In planta* efficacy not reported in accessible sections; colloidal stability metrics (ζ/PDI) unspecified.	Establishes a reproducible aqueous-extract AgNP synthesis protocol and demonstrates multi-mechanistic suppression of Xoo (growth, biofilm, motility), supporting green AgNPs as bacteriostats for rice blight management, pending *in planta* validation.	[82]

Chinese cabbage (*Brassica rapa* ssp. *pekinensis*) explants	*Pectobacterium carotovorum*	*Mahonia fortunei* extract; paper notes active ingredients include terpenoids/phenols/flavonoids and mentions berberine as an antibacterial alkaloid (quantities unspecified)	Plant-extract-mediated reduction/capping	AgNPs	HR-TEM: 6.8–21.2 nm (avg 13.19 ± 3.4 nm); DLS hydrodynamic 28–68 nm (peaks ~38.14, 43.82 nm); ζ ≈ −11.5 mV. EE/LC: not applicable.	MIC = 8 µg/mL; MBC = 8 µg/mL against P. *carotovorum*; explant assay: 10 µg/mL AgNP or 60 µg/mL antibiotic prevented soft rot on petioles; combination 2 µg/mL AgNP + 5 µg/mL zhongshengmycin suppressed infection	Strong MIC/MBC; demonstrates tissue efficacy and antibiotic dose-sparing synergy; includes DLS/ζ characterization.	Some agglomeration observed and authors suggest more optimization for consistency; broader plant-level/field validation not shown.	A well-characterized plant-extract AgNP system with low MIC/MBC that also shows synergistic soft-rot suppression on cabbage explants when combined with an antibiotic-useful for reducing antibiotic usage, though nanoparticle uniformity and field realism remain gaps.	[83]

						(synergy FIC ≈ 0.174).				
Tomato (*Solanum lycopersicum*)	*Alternaria solani*	Neem leaf extract phytochemicals (specific PSMs unspecified)	Plant-extract-mediated reduction/capping	AgNPs	Particle size reported as ~22-30 nm (paper narrative); ζ/PDI unspecified. EE/LC: not applicable.	Pot experiment: disease severity index reduced ~73%; disease incidence reduced ~69% vs control; *in vitro* inhibition increased with dose (up to ~81% at 50 ppm).	Demonstrates optimization window: low doses promote growth yield; integrates plant growth yield metrics with disease suppression.	High concentrations (25-50 ppm) associated with growth penalties/unmarketable fruit; full colloidal stability characterization incomplete (ζ/PDI not reported in extracted lines).	Shows that neem-mediated AgNPs can reduce early blight severity/incidence while improving some growth/yield traits at low ppm ranges, emphasizing the importance of dose optimization to avoid phytotoxicity or yield-quality tradeoffs.	[84]

Potato tubers, zucchini & eggplant fruits, carrot roots	*Pectobacterium carotovorum* subsp. *carotovorum* (Pcc; strain 84)	Oak (*Quercus brantii*) fruit exudates (specific PSMs unspecified)	Plant-exudate-mediated reduction/capping	AgNPs ("SNPs")	DLS: size 26.58/47.08/80.07 nm (number/volume/intensity), Z-avg 70.92 nm; PDI 0.203; ζ mean -11.40 mV. EE/LC: not applicable.	MIC 150 µg/mL; MBC 200 µg/mL. Postharvest tissues: at sub-MIC, tissue maceration reduced to ~22% (potato), 19.8% (zucchini), 21.5% (carrot), 18.5% (eggplant); when inoculation after SNPs, infection incidence reduced by 74.3% (potato), 57.2% (zucchini), 48.7%	Clear postharvest applicability; reports DLS/PDI/ζ and MIC/MBC; includes multi-host validation.	Long-term residue/ecotoxicology and real storage-chain deployment not addressed; regulatory/toxicity endpoints unspecified.	Demonstrates a practical postharvest-focused green AgNP platform with quantified MIC/MBC and multi-commodity efficacy, suggesting oak-exudate AgNPs could reduce soft-rot losses in storage/handling if safety and scale-up issues are resolved.	[85]

						(carrot), 65.1% (eggplant).				

Maize (*Zea mays*)	*Bipolaris maydis*; *Rhizoctonia solani* f. sp. *sasakii*; *Fusarium verticillioides*; *Sclerotium rolfsii*; *Macrophomina phaseolina*	Clove essential oil (major: eugenol)	EO-functionalized/grafted NP (PSM used mainly for functionalization; reduction uses ascorbate/$NaBH_4$)	Copper nanoparticles (EO-grafted CuNPs; "EGC")	DLS: hydrodynamic size ~91.28 nm; ζ ≈ −21.5 mV; PDI (polydispersity) reported as 1; TEM: CuNPs avg ~30.54 nm; EO-grafted avg ~38.48 nm. EE/LC: not applicable.	*In vitro* ED50 (µg/mL): 46.9 (B. *maydis*), 43.208 (R. *solani* f.sp. *sasakii*), 51.003 (F. *verticillioides*), 55.096 (S. *rolfsii*), 56.499 (M. *phaseolina*). Net-house: seed treatment + foliar spray at 250/500 mg L^{-1} reduced percent disease index (PDI*: disease index) by ~27.116 and 25.292% across two	Multi-pathogen spectrum; includes net-house multi-season evidence; highlights dose-sparing potential and defense induction markers.	Synthesis not purely "green" (uses $NaBH_4$); phytotoxicity at higher doses; Cu bioaccumulation concerns flagged by authors.	An EO-enabled CuNP platform that combines strong *in vitro* antifungal potency (ED50 range ~43-56 µg/mL) with net-house disease reductions and host-defense activation, but requires careful dose optimization and attention to copper accumulation and "green" chemistry claims.	[86]

						seasons; 1,000 mg L⁻¹ best PDI* but noted phytotoxicity.				
Chickpea (*Cicer arietinum*)	*Fusarium oxysporum* f. sp. *ciceris*	Roasted coffee extract; EGCG identified as reducing /capping agent	Plant-product-mediated reduction/capping	CuO nanoparticles	Size ~85–100 nm; ζ ≈ −25.3 mV; PDI unspecified; morphology unspecified. EE/LC: not applicable.	Root immersion in seedlings: 10 ppm and 25 ppm reduced wilt incidence by ~74.5% and 50%, respectively; 10 ppm increased chlorophyll (~+50.08% reported) while higher doses reduced chlorophyll.	Demonstrates a low-dose “nanopriming”/root-treatment window enabling disease suppression plus growth benefits; includes ζ characterization.	Many formulation descriptors (PDI, detailed synthesis ratios) not in extracted abstract; field validation and environmental fate unspecified.	A coffee-extract/EGCG-enabled CuO NP synthesis approach shows strong dose-dependent chickpea wilt suppression at low ppm ranges, reinforcing the concept that “green” CuO NPs can act as both protective agents and growth modulators when carefully dosed.	[87]

Tomato (*Solanum lycopersicum*)	*Alternaria solani*	Quercus incana leaf extract phytochemicals (specific PSMs unspecified)	Plant-extract-mediated reduction/capping	AgNPs	AgNPs ~27 nm (mean; round); ζ/PDI unspecified. EE/LC: not applicable.	Petri assay (100 mg/L): mycelial growth inhibition 98.27±1.58% (colony diameter 1.50±2.33 mm vs control 86.67±2.22 mm). Leaflet/greenhouse spray: inhibition rate 92.79±1.33% at 100 mg/L; $AgNO_3$ control showed negligible inhibition	Strong comparative efficacy vs ionic Ag control; clearly reported *in vitro* and greenhouse leaf assay with quantified inhibition.	Full-season yield/field performance not tested; colloidal stability (ζ/PDI) not reported in extracted PDF.	Provides a straightforward aqueous-leaf protocol yielding ~27 nm AgNPs with high antifungal inhibition against A. *solani* in both Petri and greenhouse leaflet assays, supporting AgNPs as candidate nano-fungicides pending field realism and stability reporting.	[88]

						(≈1.55±0.67 %).				

Tomato (*Solanum lycopersicum*; cv. TH99806)	*Fusarium solani*	Zizyphus spina-christi leaf extract phytochemicals (specific compounds unspecified)	Plant-extract-mediated reduction/capping	CuO nanoparticles	TEM size ~13.4-30.9 nm; DLS mean ~34.1±4.379 nm; PDI/ζ unspecified. EE/LC: not applicable.	*In vitro* mycelial inhibition: ~91.17±1.28 % at 250 mg/L (vs 88.20±3.57% chemical fungicide; 77.09±5.88% Trichoderma biocide). Greenhouse: disease reduction 72.0-88.6%; at 250 mg/L disease severity ~9.17±2.89% and incidence ~4.17±3.80% (infected control severity 80.5%).	Strong greenhouse efficacy with quantified incidence/severity; includes growth/physiology benefits and benchmarks vs fungicide/biocide.	Full nanoparticle surface-charge/stability parameters not reported in extracted lines; longer-term soil/ecotoxicology beyond short-term exposure remains limited.	A well-benchmarked CuO NP system made via Zizyphus leaf extract provides strong *in vitro* and greenhouse suppression of tomato F. *solani* root rot, with competitive performance relative to standard copper fungicide/biocide controls at optimized doses.	[89]

Cabbage (*Brassica oleracea* var. *capitata*)	*Xanthomonas campestris* pv. *campestris* (Xcc; isolate Xcc 51)	Aqueous extracts of cabbage, *Arabidopsis thaliana*, neem, noni (leaf/fruit parts); specific PSMs not quantified (complex mixture)	Plant-extract-mediated reduction/capping	AgNPs	DLS/physchem: hydrodynamic diameter varies widely by extract (≈168.3–4636 nm); best-performing had HD ~192.3±1.1 nm at 64 µmol L^{-1}. PDI ranges ~0.255–0.780 (by extract). ζ range ~−11.2 to −23.7 mV. EE/LC: not applicable.	MIC screening up to 256 µmol L^{-1}; best-performing AENFP-AgNPs showed highest antibacterial activity at 64 µmol L^{-1}; cabbage leaf challenge: AENFP-AgNPs reduced injured foliar area and delayed symptoms up to 7 days after inoculation (quantified as % injured area; exact values not in extracted lines).	Links physicochemical metrics (HD/PDI/ζ) with antibacterial activity; demonstrates host defense priming (RT-qPCR marker genes) and *in planta* symptom suppression.	Protective effect described as temporary (symptom "normalization" later), implying persistence limits; wide size distributions in some suspensions; field-scale validation not shown.	By systematically varying extract sources and concentrations, this study connects AgNP colloidal features (HD/PDI/ζ) to MIC-level antibacterial activity and short-term *in planta* black-rot suppression, while highlighting persistence and formulation homogeneity as key translation bottlenecks.	[90]

References

[1] C.-G. Song *et al.*, “Anti-TMV Activity and Functional Mechanisms of Two Diterpenoid Alkaloids Isolated from *Dendrobium findlayanum*,” *J. Agric. Food Chem.*, vol. 73, no. 4, pp. 2443–2450, Jan. 2025, doi: 10.1021/acs.jafc.4c12988.

[2] A. A. Al-Askar *et al.*, “Rosemary essential oil: chemical composition, antiviral, cytotoxicity, and in silico molecular docking analysis against tobacco mosaic virus,” *Sci. Rep.*, vol. 15, no. 1, p. 40048, Nov. 2025, doi: 10.1038/s41598-025-24071-y.

[3] W. Guo, X. Lu, B. Liu, H. Yan, and J. Feng, “<scp>Anti-TMV</scp> activity and mode of action of three alkaloids isolated from <scp> *Chelidonium majus* </scp>,” *Pest Manag. Sci.*, vol. 77, no. 1, pp. 510–517, Jan. 2021, doi: 10.1002/ps.6049.

[4] Q.-F. Hu *et al.*, “Antivirus isoindolinone alkaloids with rare oxocyclopenta[f]isoindole frameworks isolated from the stems of flue cured tobacco,” *Chemical and Biological Technologies in Agriculture*, vol. 9, no. 1, p. 88, Nov. 2022, doi: 10.1186/s40538-022-00339-7.

[5] A. Taglienti *et al.*, “In vivo Antiphytoviral Activity of Essential Oils and Hydrosols From Origanum vulgare, Thymus vulgaris, and Rosmarinus officinalis to Control Zucchini Yellow Mosaic Virus and Tomato Leaf Curl New Delhi Virus in Cucurbita pepo L.,” *Front. Microbiol.*, vol. 13, Apr. 2022, doi: 10.3389/fmicb.2022.840893.

[6] Y. Yan *et al.*, “Anti-TMV activity and effects of three prieurianin-type limonoids from Munronia henryi,” *Pestic. Biochem. Physiol.*, vol. 184, p. 105108, Jun. 2022, doi: 10.1016/j.pestbp.2022.105108.

[7] P. Song, X. Yu, W. Yang, and Q. Wang, “Natural phytoalexin stilbene compound resveratrol and its derivatives as anti-tobacco mosaic virus and anti-phytopathogenic fungus agents,” *Sci. Rep.*, vol. 11, no. 1, p. 16509, Aug. 2021, doi: 10.1038/s41598-021-96069-1.

[8] S. Mostafaee, F. Rakhshandehroo, H. Rezadoost, and M. Farzaneh, “Anti-Phytoviral Activity of Hydroalcoholic Extract and Essential Oil Nanoemulsion of Yarrow (*Achillea millefolium* L.) Against Cucumber Mosaic Virus,” *Journal of Phytopathology*, vol. 173, no. 6, Nov. 2025, doi: 10.1111/jph.70187.

[9] M. Ekemen and Ç. Ulubaş Serçe, “A comprehensive investigation of the effect of thyme oil-loaded chitosan nanoparticles on aphid-mediated transmission of potato virus Y,” *Pest Manag. Sci.*, vol. 82, no. 4, pp. 3978–3992, Apr. 2026, doi: 10.1002/ps.70530.

[10] X. Y. Wang *et al.*, “Crude garlic extract significantly inhibits replication of grapevine viruses,” *Plant Pathol.*, vol. 69, no. 1, pp. 149–158, Jan. 2020, doi: 10.1111/ppa.13103.

[11] V. Iobbi *et al.*, “Characterization of sulfur compounds and antiviral activity against Tomato brown rugose fruit virus (ToBRFV) of Italian ‘Vessalico’ garlic compared to other cultivars and landrace,” *LWT*, vol. 174, p. 114411, Jan. 2023, doi: 10.1016/j.lwt.2022.114411.

[12] A. Liao, L. Li, T. Wang, A. Lu, Z. Wang, and Q. Wang, “Discovery of Phytoalexin Camalexin and Its Derivatives as Novel Antiviral and Antiphytopathogenic-Fungus Agents,” *J. Agric. Food Chem.*, vol. 70, no. 8, pp. 2554–2563, Mar. 2022, doi: 10.1021/acs.jafc.1c07805.

[13] J. Wang *et al.*, “Field application of nanoliposomes delivered quercetin by inhibiting specific hsp70 gene expression against plant virus disease,” *J. Nanobiotechnology*, vol. 20, no. 1, p. 16, Dec. 2022, doi: 10.1186/s12951-021-01223-6.

[14] Z. Xia *et al.*, “Discovery of Resveratrol and its Derivatives as Novel Antiviral and Anti- Phytopathogenic-Fungus Agents,” *Curr. Org. Synth.*, vol. 20, no. 3, pp. 351–359, May 2023, doi: 10.2174/1570179420666221005124445.

[15] L. Zhao *et al.*, “Berberine from Corydalis saxicola Bunting reveals anti-tomato spotted wilt orthotospovirus activity with a novel mechanism,” *Plant Physiology and Biochemistry*, vol. 227, p. 110119, Oct. 2025, doi: 10.1016/j.plaphy.2025.110119.

[16] V. Iobbi *et al.*, “Autoxidation Products of the Methanolic Extract of the Leaves of Combretum micranthum Exert Antiviral Activity against Tomato Brown Rugose Fruit Virus (ToBRFV),” *Molecules*, vol. 27, no. 3, p. 760, Jan. 2022, doi: 10.3390/molecules27030760.

[17] C.-M. Kang and R.-D. Jeong, "Inhibitory Effects of Garlic Extract on Hop Stunt Viroid in Micropropagated Grapevine Plantlets," *Plant Pathol. J.*, vol. 41, no. 1, pp. 51–63, Feb. 2025, doi: 10.5423/PPJ.OA.10.2024.0169.

[18] V. Bunevičius, A. Morkeliūnė, J. Griauzdaitė, A. Valiuškaitė, and N. Rasiukevičiūtė, "The Use of Clove and Rosemary Plant Extracts Against Colletotrichum acutatum and Botrytis cinerea," Agronomy, vol. 15, no. 7, p. 1728, Jul. 2025, doi: 10.3390/agronomy15071728.https://doi.org/10.3390/agronomy15071728

[19] A. El Khetabi et al., "In Vitro and In Vivo Antifungal Activities of Nine Commercial Essential Oils against Brown Rot in Apples," Horticulturae, vol. 7, no. 12, p. 545, Dec. 2021, doi: 10.3390/horticulturae7120545.

[20] J. Yan, H. Wu, K. Chen, J. Feng, and Y. Zhang, "Antifungal Activities and Mode of Action of Cymbopogon citratus, Thymus vulgraris, and Origanum heracleoticum Essential Oil Vapors against Botrytis cinerea and Their Potential Application to Control Postharvest Strawberry Gray Mold," *Foods*, vol. 10, no. 10, p. 2451, Oct. 2021, doi: 10.3390/foods10102451.

[21] M. Mirmajlessi, N. Najdabbasi, L. Sigillo, and G. Haesaert, "An implementation framework for evaluating the biocidal potential of essential oils in controlling Fusarium wilt in spinach: from in vitro to in planta," *Front. Plant Sci.*, vol. 15, Aug. 2024, doi: 10.3389/fpls.2024.1444195.

[22] N. Tzortzakis, "Origanum dictamnus Essential Oil in Vapour or Aqueous Solution Application for Pepper Fruit Preservation against Botrytis cinerea," *Agronomy*, vol. 14, no. 2, p. 257, Jan. 2024, doi: 10.3390/agronomy14020257.

[23] J. M. A. Hoyos et al., "Antifungal activity of essential oils in Colletotrichum lindemuthianum and alternative control of bean anthracnose," Eur. J. Plant Pathol., vol. 172, no. 2, pp. 373-389, Jun. 2025, doi: 10.1007/s10658-025-03010-7.

[24] H. Soleimani, R. Mostowfizadeh-Ghalamfarsa, M. Ghanadian, A. Karami, and S. O. Cacciola, "Defense Mechanisms Induced by Celery Seed Essential Oil against Powdery Mildew Incited by Podosphaera fusca in Cucumber," Journal of Fungi, vol. 10, no. 1, p. 17, Dec. 2023, doi: 10.3390/jof10010017.

[25] P. K. Christova, A. M. Dobreva, A. G. Dzhurmanski, I. N. Dincheva, S. D. Dimkova, and N. G. Zapryanova, "The Impact of Plant Essential Oils on the Growth of the Pathogens Botrytis cinerea, Fusarium solani, and Phytophthora pseudocryptogea," Life, vol. 14, no. 7, p. 817, Jun. 2024, doi: 10.3390/life14070817.

[26] K. Połeć *et al.*, "The influence of ergosterol on the action of the hop oil and its major terpenes on model fungi membranes. Towards understanding the mechanism of action of phytocompounds for food and plant protection," *Chem. Phys. Lipids*, vol. 238, p. 105092, Aug. 2021, doi: 10.1016/j.chemphyslip.2021.105092.

[27] W. Cely-Veloza, D. Quiroga, and E. Coy-Barrera, "Quinolizidine-Based Variations and Antifungal Activity of Eight Lupinus Species Grown under Greenhouse Conditions," *Molecules*, vol. 27, no. 1, p. 305, Jan. 2022, doi: 10.3390/molecules27010305.

[28] F. A. Bernal and E. Coy-Barrera, "Composition and Antifungal Activity of the Alkaloidal Fraction of Lupinus mirabilis Leaves: A Biochemometrics-Based Exploration," *Molecules*, vol. 27, no. 9, p. 2832, Apr. 2022, doi: 10.3390/molecules27092832.

[29] G. V. Buitimea-Cantúa *et al.*, "The aflatoxin inhibitors capsaicin and piperine from *Capsicum chinense* and *Piper nigrum* fruits modulate the antioxidant system in *Aspergillus parasiticus*," *Journal of Environmental Science and Health, Part B*, vol. 57, no. 5, pp. 358–368, May 2022, doi: 10.1080/03601234.2022.2060029.

[30] A. Falcón-Piñeiro *et al.*, "Antifungal Activity of Propyl-Propane-Thiosulfinate (PTS) and Propyl-Propane-Thiosulfonate (PTSO) from Allium cepa against Verticillium dahliae: In Vitro and in Planta Assays," *Journal of Fungi*, vol. 7, no. 9, p. 736, Sep. 2021, doi: 10.3390/jof7090736.

[31] N. Jamshidi, M. Darvishnia, E. Bazgir, H. Mirzaei Najafgholi, M. H. Ghodoum Parizipour, and M. Aeini, "Antibacterial activity of some plant-derived essential oils against plant pathogenic bacteria," *Journal of Plant Diseases and Protection*, vol. 130, no. 4, pp. 853–865, Aug. 2023, doi: 10.1007/s41348-023-00765-1.

[32] N. Puvača *et al.*, "Antimicrobial Activity of Selected Essential Oils against Selected Pathogenic Bacteria: In Vitro Study," *Antibiotics*, vol. 10, no. 5, p. 546, May 2021, doi: 10.3390/antibiotics10050546.

[33] N. P. Mangalagiri, S. K. Panditi, and N. L. L. Jeevigunta, "Antimicrobial activity of essential plant oils and their major components," *Heliyon*, vol. 7, no. 4, p. e06835, Apr. 2021, doi: 10.1016/j.heliyon.2021.e06835.

[34] C. De Clerck, S. D. Maso, O. Parisi, F. Dresen, A. Zhiri, and M. H. Jijakli, “Screening of Antifungal and Antibacterial Activity of 90 Commercial Essential Oils against 10 Pathogens of Agronomical Importance,” *Foods*, vol. 9, no. 10, p. 1418, Oct. 2020, doi: 10.3390/foods9101418.

[35] W. Yakhlef, R. Arhab, C. Romero, M. Brenes, A. de Castro, and E. Medina, “Phenolic composition and antimicrobial activity of Algerian olive products and by-products,” *LWT*, vol. 93, pp. 323–328, Jul. 2018, doi: 10.1016/j.lwt.2018.03.044.

[36] T. Benali *et al.*, “Chemical composition and antibacterial activity of the essential oil and extracts of Cistus ladaniferus subsp. ladanifer and Mentha suaveolens against phytopathogenic bacteria and their ecofriendly management of phytopathogenic bacteria,” *Biocatal. Agric. Biotechnol.*, vol. 28, p. 101696, Sep. 2020, doi: 10.1016/j.bcab.2020.101696.

[37] B. Caballero-Guerrero *et al.*, “Antimicrobial effects of treated olive mill waste on foodborne pathogens,” *LWT*, vol. 164, p. 113628, Jul. 2022, doi: 10.1016/j.lwt.2022.113628.

[38] T. Ditsawanon, S. Roytrakul, N. Phaonakrop, S. Charoenlappanit, S. Thaisakun, and N. Parinthawong, “Novel Small Antimicrobial Peptides Extracted from Agricultural Wastes Act against Phytopathogens but Not Rhizobacteria,” *Agronomy*, vol. 12, no. 8, p. 1841, Aug. 2022, doi: 10.3390/agronomy12081841.

[39] I. Chiocchio, M. Mandrone, M. Tacchini, A. Guerrini, and F. Poli, “Phytochemical Profile and In Vitro Bioactivities of Plant-Based By-Products in View of a Potential Reuse and Valorization,” *Plants*, vol. 12, no. 4, p. 795, Feb. 2023, doi: 10.3390/plants12040795.

[40] H. Terea, D. Selloum, A. Rebiai, A. Bouafia, and O. Ben Mya, “Preparation and characterization of cellulose/ZnO nanoparticles extracted from peanut shells: effects on antibacterial and antifungal activities,” *Biomass Convers. Biorefin.*, vol. 14, no. 16, pp. 19489–19500, Aug. 2024, doi: 10.1007/s13399-023-03959-7.

[41] M. Ul-Islam *et al.*, “Development of low-cost bacterial cellulose-pomegranate peel extract-based antibacterial composite for potential biomedical applications,” *Int. J. Biol. Macromol.*, vol. 231, p. 123269, Mar. 2023, doi: 10.1016/j.ijbiomac.2023.123269.

[42] Z. Najmi *et al.*, “Screening of Different Essential Oils Based on Their Physicochemical and Microbiological Properties to Preserve Red Fruits and Improve Their Shelf Life,” *Foods*, vol. 12, no. 2, p. 332, Jan. 2023, doi: 10.3390/foods12020332.

[43] S. Caparrotta *et al.*, “Correlation between VOC fingerprinting and antimicrobial activity of several essential oils extracted by plant resins against *A. tumefaciens* and *P. savastanoi*,” *Flavour Fragr. J.*, vol. 34, no. 5, pp. 377–387, Sep. 2019, doi: 10.1002/ffj.3518.

[44] D. Gruľová *et al.*, “Thymol Chemotype Origanum vulgare L. Essential Oil as a Potential Selective Bio-Based Herbicide on Monocot Plant Species,” *Molecules*, vol. 25, no. 3, p. 595, Jan. 2020, doi: 10.3390/molecules25030595.

[45] R. I. Tarakanov and F. S.-U. Dzhalilov, “Using of Essential Oils and Plant Extracts against Pseudomonas savastanoi pv. glycinea and Curtobacterium flaccumfaciens pv. flaccumfaciens on Soybean,” *Plants*, vol. 11, no. 21, p. 2989, Nov. 2022, doi: 10.3390/plants11212989.

[46] H. K. Patel *et al.*, “Volatile metabolites from new cultivars of catnip and oregano as potential antibacterial and insect repellent agents,” *Front. Plant Sci.*, vol. 14, Feb. 2023, doi: 10.3389/fpls.2023.1124305.

[47] E. Sánchez-Hernández *et al.*, “Antibacterial Activity of Ginkgo biloba Extracts against Clavibacter michiganensis subsp. michiganensis, Pseudomonas spp., and Xanthomonas vesicatoria,” *Horticulturae*, vol. 9, no. 4, p. 461, Apr. 2023, doi: 10.3390/horticulturae9040461.

[48] C. Bouchekouk, F. Z. Kara, G. Tail, F. Saidi, and T. Benabdelkader, “Essential oil composition and antibacterial activity of *Pteridium aquilinum* (L.) Kuhn,” *Biol. Futur.*, vol. 70, no. 1, pp. 56–61, Mar. 2019, doi: 10.1556/019.70.2019.07.

[49] H. Feizi, V. Tahan, and K. Kariman, “*In vitro* antibacterial activity of essential oils from *Carum copticum* and *Ziziphora clinopodioides* plants against the phytopathogen *Pseudomonas syringae pv. syringae*,” *Plant Biosystems - An International Journal Dealing with all Aspects of Plant Biology*, vol. 157, no. 2, pp. 487–492, Mar. 2023, doi: 10.1080/11263504.2023.2165564.

[50] R. Fontana *et al.*, “Control of Erwinia amylovora Growth by Moringa oleifera Leaf Extracts: In Vitro and in Planta Effects,” *Plants*, vol. 11, no. 7, p. 957, Mar. 2022, doi: 10.3390/plants11070957.

[51] A. L. Morales-Ubaldo *et al.*, “Bactericidal Activity of Larrea tridentata Hydroalcoholic Extract against Phytopathogenic Bacteria,” *Agronomy*, vol. 11, no. 5, p. 957, May 2021, doi: 10.3390/agronomy11050957.

[52] A. Ben Hsouna, N. Touj, I. Hammami, K. Dridi, A. S. Al-Ayed, and N. Hamdi, “Chemical Composition and in vivo Efficacy of the Essential Oil of Mentha piperita L. in the Suppression of Crown Gall Disease on Tomato Plants,” J. Oleo Sci., vol. 68, no. 5, pp. 419–426, 2019, doi: 10.5650/jos.ess18261.

[53] S. Kumari *et al.*, “Thymol nanoemulsion exhibits potential antibacterial activity against bacterial pustule disease and growth promotory effect on soybean,” *Sci. Rep.*, vol. 8, no. 1, p. 6650, Apr. 2018, doi: 10.1038/s41598-018-24871-5.

[54] Y. Long, W. Huang, Q. Wang, and G. Yang, “Green synthesis of garlic oil nanoemulsion using ultrasonication technique and its mechanism of antifungal action against Penicillium italicum,” *Ultrason. Sonochem.*, vol. 64, p. 104970, Jun. 2020, doi: 10.1016/J.ULTSONCH.2020.104970.

[55] A. B. Perumal, X. Li, Z. Su, and Y. He, “Preparation and characterization of a novel green tea essential oil nanoemulsion and its antifungal mechanism of action against Magnaporthae oryzae,” *Ultrason. Sonochem.*, vol. 76, p. 105649, Aug. 2021, doi: 10.1016/J.ULTSONCH.2021.105649.

[56] E.-S. H. E. Ziedan, M. M. Saad, A. A. El-Kafrawy, A. F. Sahab, and A.-T. H. Mossa, “Evaluation of essential oils nanoemulsions formulations on Botrytis cinerea growth, pathology and grey mould incidence on cucumber fruits,” *Bulletin of the National Research Centre 2022 46:1*, vol. 46, no. 1, pp. 88-, Apr. 2022, doi: 10.1186/S42269-022-00765-5.

[57] J. Zhang *et al.*, “Nano-Thymol Emulsion Inhibits Botrytis cinerea to Control Postharvest Gray Mold on Tomato Fruit,” *Agronomy 2022, Vol. 12, Page 2973*, vol. 12, no. 12, p. 2973, Nov. 2022, doi: 10.3390/AGRONOMY12122973.

[58] M. A. Mosa, K. Youssef, S. F. Hamed, and A. F. Hashim, “Antifungal activity of eco-safe nanoemulsions based on Nigella sativa oil against Penicillium verrucosum infecting maize seeds: Biochemical and physiological traits,” *Front. Microbiol.*, vol. 13, p. 1108733, Jan. 2023, doi: 10.3389/FMICB.2022.1108733/TEXT.

[59] Q. Le Dang *et al.*, “A botanical nanoemulsion against phytopathogenic fungi Colletotrichum sp. and Fusarium oxysporum: Preparation, in vitro and in vivo bioassay,” *Journal of Natural Pesticide Research*, vol. 10, p. 100099, Dec. 2024, doi: 10.1016/J.NAPERE.2024.100099.

[60] A.-Q. Dao *et al.*, “Optimization of lemongrass essential oil nanoemulsion formulation and antifungal activity against Colletotrichum musae QB6 causing anthracnose in bananas,” *J. King Saud Univ. Sci.*, vol. 37, no. 10, Dec. 2025, doi: 10.25259/JKSUS_1151_2025.

[61] S. Gumede, S. Mpai, S. Kesavan Pillai, and D. Sivakumar, “Nano Emulsion of Essential Oils Loaded in Chitosan Coating for Controlling Anthracnose in Tomatoes (Solanum lycopersicum) During Storage,” *Foods 2025, Vol. 14, Page 3038*, vol. 14, no. 17, p. 3038, Aug. 2025, doi: 10.3390/FOODS14173038.

[62] A. T. Abbasi, L. Ebrahimi, and M. Farzaneh, “Antifungal efficacy of plant essential oil nanoemulsions against cucumber powdery mildew,” *Sci. Rep.*, vol. 15, no. 1, Dec. 2025, doi: 10.1038/S41598-025-24039-Y.

[63] M. Mondéjar-López *et al.*, “Chitosan Nanoparticles Loaded with Sideritis serrata Lag. Extract: A New Eco-Friendly Antifungal Agent for Sustainable Agriculture,” *Plants*, vol. 14, no. 24, p. 3757, Dec. 2025, doi: 10.3390/PLANTS14243757.

[64] N. K. Kalagatur, O. S. Nirmal Ghosh, N. Sundararaj, and V. Mudili, “Antifungal activity of chitosan nanoparticles encapsulated with Cymbopogon martinii essential oil on plant pathogenic fungi Fusarium graminearum,” *Front. Pharmacol.*, vol. 9, no. JUN, p. 356156, Jun. 2018, doi: 10.3389/FPHAR.2018.00610/TEXT.

[65] S. Dou, Q. Ouyang, K. You, J. Qian, and N. Tao, “An inclusion complex of thymol into β-cyclodextrin and its antifungal activity against Geotrichum citri-aurantii,” *Postharvest Biol. Technol.*, vol. 138, pp. 31–36, Apr. 2018, doi: 10.1016/J.POSTHARVBIO.2017.12.011.

[66] W. Weisany, S. Samadi, J. Amini, S. Hossaini, S. Yousefi, and F. Maggi, “Enhancement of the antifungal activity of thyme and dill essential oils against Colletotrichum nymphaeae by nano-encapsulation with copper NPs,” *Ind. Crops Prod.*, vol. 132, pp. 213–225, Jun. 2019, doi: 10.1016/J.INDCROP.2019.02.031.

[67] V. Serna-Escolano *et al.*, "Thymol Encapsulated into HP-β-Cyclodextrin as an Alternative to Synthetic Fungicides to Induce Lemon Resistance against Sour Rot Decay," *Molecules 2020, Vol. 25, Page 4348*, vol. 25, no. 18, p. 4348, Sep. 2020, doi: 10.3390/MOLECULES25184348.

[68] P. R. Oliveira-Pinto *et al.*, "Satureja montana Essential Oil, Zein Nanoparticles and Their Combination as a Biocontrol Strategy to Reduce Bacterial Spot Disease on Tomato Plants," *Horticulturae 2021, Vol. 7, Page 584*, vol. 7, no. 12, p. 584, Dec. 2021, doi: 10.3390/HORTICULTURAE7120584.

[69] S. Abd-Ellatif *et al.*, "Green Synthesized of Thymus vulgaris Chitosan Nanoparticles Induce Relative WRKY-Genes Expression in Solanum lycopersicum against Fusarium solani, the Causal Agent of Root Rot Disease," *Plants*, vol. 11, no. 22, p. 3129, Nov. 2022, doi: 10.3390/PLANTS11223129/S1.

[70] Y. Zhang, Y. Tan, O. O. Reymick, Q. Ouyang, and N. Tao, "γ-Cyclodextrin-Encapsulated Cinnamaldehyde for Citrus Preservation and Its Potential Mechanisms against Penicillium digitatum," *Journal of Fungi 2022, Vol. 8, Page 1199*, vol. 8, no. 11, p. 1199, Nov. 2022, doi: 10.3390/JOF8111199.

[71] A. M. Vettraino, F. Zikeli, G. Scarascia Mugnozza, V. Vinciguerra, D. Tabet, and M. Romagnoli, "Lignin nanoparticles containing essential oils for controlling Phytophthora cactorum diseases," *For. Pathol.*, vol. 52, no. 2, p. e12739, Apr. 2022, doi: 10.1111/EFP.12739;ISSUE:ISSUE:DOI.

[72] S. Falsini *et al.*, "Tannins-lignin mixed nanoformulations for improving the potential of neem oil as fungicide agent," *Environ. Sci. Pollut. Res. Int.*, vol. 30, no. 13, pp. 39131–39141, Mar. 2023, doi: 10.1007/S11356-022-24991-6.

[73] F. Zikeli *et al.*, "Lignin Nanoparticles with Entrapped Thymus spp. Essential Oils for the Control of Wood-Rot Fungi," *Polymers 2023, Vol. 15, Page 2713*, vol. 15, no. 12, p. 2713, Jun. 2023, doi: 10.3390/POLYM15122713.

[74] D. C. Punelas–Villanueva, R. A. Almeda, M. S. S. Chua, R. F. Tabor, M. L. P. Vidallon, and E. B. Rodriguez, "Zein nanoencapsulation enhances the antifungal activity of thymol for postharvest decay control in bananas," *Emergent Materials 2024 8:3*, vol. 8, no. 3, pp. 1577–1590, Sep. 2024, doi: 10.1007/S42247-024-00851-6.

[75] H. Soleimani, R. Mostowfizadeh-Ghalamfarsa, and A. Zarei, "Chitosan nanoparticle encapsulation of celery seed essential oil: Antifungal activity and defense mechanisms against cucumber powdery mildew," *Carbohydrate Polymer Technologies and Applications*, vol. 8, p. 100531, Dec. 2024, doi: 10.1016/J.CARPTA.2024.100531.

[76] J. Cai, D. Yang, and Q. Wang, "Preparation and characterization of chitosan nanoparticles loaded with Athyrium sinense essential oil with antibacterial properties against Pectobacterium carotovorum subsp. carotovorum," *Ind. Crops Prod.*, vol. 195, p. 116382, May 2023, doi: 10.1016/J.INDCROP.2023.116382.

[77] Y. Liu, L. Weng, Y. Lin, D. Lin, L. Xie, and T. Zhong, "Carvacrol/β-cyclodextrin inclusion complex as a fumigant to control decay caused by Penicillium digitatum on Shatangju mandarin slices," *Heliyon*, vol. 9, no. 8, p. e18804, Aug. 2023, doi: 10.1016/J.HELIYON.2023.E18804.

[78] H. Soleimani, R. Mostowfizadeh-Ghalamfarsa, and M. Ghanadian, "Characterization, biochemical defense mechanisms, and antifungal activities of chitosan nanoparticle-encapsulated spinach seed essential oil," *J. Agric. Food Res.*, vol. 22, p. 102016, Aug. 2025, doi: 10.1016/J.JAFR.2025.102016.

[79] Y. Luo, Y. Zhang, X. Chen, J. Che, and N. Tao, "Preparation, characterization, and antifungal activity of (E)-2-octenal/β-cyclodextrin inclusion complex against the blue mold in citrus fruit caused by a prochloraz-resistant Penicillium italicum strain," *J. Appl. Microbiol.*, vol. 136, no. 5, p. 115, May 2025, doi: 10.1093/JAMBIO/LXAF115.

[80] T. R. Santiago *et al.*, "Green synthesis of silver nanoparticles using tomato leaf extract and their entrapment in chitosan nanoparticles to control bacterial wilt," *J. Sci. Food Agric.*, vol. 99, no. 9, pp. 4248–4259, Jul. 2019, doi: 10.1002/JSFA.9656.

[81] N. Issam, D. Naceur, G. Nechi, S. Maatalah, K. Zribi, and H. Mhadhbi, "Green synthesised ZnO nanoparticles mediated by Olea europaea leaf extract and their antifungal activity against Botrytis cinerea infecting faba bean plants," *Archives of Phytopathology and Plant Protection*, vol. 54, no. 15–16, pp. 1083–1105, 2021, doi: 10.1080/03235408.2021.1889859.

[82] Y. Tian *et al.*, “Bioinspired Green Synthesis of Silver Nanoparticles Using Three Plant Extracts and Their Antibacterial Activity against Rice Bacterial Leaf Blight Pathogen Xanthomonas oryzae pv. oryzae,” *Plants 2022, Vol. 11, Page 2892*, vol. 11, no. 21, p. 2892, Oct. 2022, doi: 10.3390/PLANTS11212892.

[83] Z. Wei, S. Xu, H. Jia, and H. Zhang, “Green synthesis of silver nanoparticles from Mahonia fortunei extracts and characterization of its inhibitory effect on Chinese cabbage soft rot pathogen,” *Front. Microbiol.*, vol. 13, p. 1030261, Oct. 2022, doi: 10.3389/FMICB.2022.1030261/TEXT.

[84] M. Ansari *et al.*, “Green Synthesized Silver Nanoparticles: A Novel Approach for the Enhanced Growth and Yield of Tomato against Early Blight Disease,” *Microorganisms 2023, Vol. 11, Page 886*, vol. 11, no. 4, p. 886, Mar. 2023, doi: 10.3390/MICROORGANISMS11040886.

[85] M. Soltani Nejad *et al.*, “Application of Biosynthesized Silver Nanoparticles from Oak Fruit Exudates against Pectobacterium carotovorum subsp. carotovorum Causing Postharvest Soft Rot Disease in Vegetables,” *Agronomy 2023, Vol. 13, Page 1624*, vol. 13, no. 6, p. 1624, Jun. 2023, doi: 10.3390/AGRONOMY13061624.

[86] L. Dorjee *et al.*, “Essential oil-grafted copper nanoparticles as a potential next-generation fungicide for holistic disease management in maize,” *Front. Microbiol.*, vol. 14, p. 1204512, Jul. 2023, doi: 10.3389/FMICB.2023.1204512/FULL.

[87] V. Tiwari *et al.*, “Application of green synthesized copper oxide nanoparticles for effective mitigation of Fusarium wilt disease in roots of Cicer arietinum,” *Physiol. Mol. Plant Pathol.*, vol. 131, p. 102244, May 2024, doi: 10.1016/J.PMPP.2024.102244.

[88] J. Khatoon *et al.*, “Green-fabricated silver nanoparticles from Quercus incana leaf extract to control the early blight of tomatoes caused by Alternaria solani,” *BMC Plant Biology 2024 24:1*, vol. 24, no. 1, pp. 302-, Apr. 2024, doi: 10.1186/S12870-024-05008-5.

[89] S. E. El-Abeid, M. A. Mosa, M. A. M. El-Tabakh, A. M. Saleh, M. A. El-Khateeb, and M. S. A. Haridy, “Antifungal activity of copper oxide nanoparticles derived from Zizyphus spina leaf extract against Fusarium root rot disease in tomato plants,” *Journal of Nanobiotechnology 2024 22:1*, vol. 22, no. 1, pp. 28-, Jan. 2024, doi: 10.1186/S12951-023-02281-8.

[90] I. R. Santos, F. T. P. K. Tavora, E. A. F. Severo, O. B. Oliveira-Neto, A. Mehta, and L. P. Silva, “Green synthesis of silver nanoparticles and their potential to enhance defense in cabbage crop against Xanthomonas campestris pv. campestris,” *Discover Plants 2025 2:1*, vol. 2, no. 1, pp. 105-, Apr. 2025, doi: 10.1007/S44372-025-00190-8.
